# COVID-19’s U.S. Temperature Response Profile

**DOI:** 10.1007/s10640-021-00603-8

**Published:** 2021-09-20

**Authors:** Richard T. Carson, Samuel L. Carson, Thayne K. Dye, Samuel A. Mayfield, Daniel C. Moyer, Chu A. Yu

**Affiliations:** 1 Department of Economics, University of California, San Diego, La Jolla, CA USA; 2 Independent Consultant, Minneapolis, MN USA; 3 Independent Consultant, Riverside, CA USA; 4 Computer Science and Artificial Intelligence Laboratory, Massachusetts Institute of Technology, Cambridge, MA USA; 5 Department of Economics, Wake Forest University, Winston Salem, NC USA

**Keywords:** Data temporal alignment, Epidemiology, Forecasting, Temperature sensitivity

## Abstract

We estimate the U.S. temperature response profile (TRP) for COVID-19 and show it is highly sensitive to temperature variation. Replacing the erratic daily death counts U.S. states initially reported with counts based on death certificate date, we build a week-ahead statistical forecasting model that explains most of their daily variation (R^2^ = 0.97) and isolates COVID-19’s TRP (*p* < 0.001). These counts, normalized at 31 °C (U.S. mid-summer average), scale up to 160% at 5 °C in the static case where the infection pool is held constant. Positive case counts are substantially more temperature sensitive. When temperatures are declining, dynamic feedback through a growing infection pool can substantially amplify these temperature effects. Our estimated TRP can be incorporated into COVID-related planning exercises and used as an input to SEIR models employed for longer run forecasting. For the former, we show how our TRP is predictive of the realized pattern of growth rates in per capita positive cases across states five months after the end of our sample period. For the latter, we show the variation in herd immunity levels implied by temperature-driven, time-varying R_0_ series for the Alpha and Delta variants of COVID-19 for several representative states.

**Supplementary Information:**

The online version contains supplementary material available at 10.1007/s10640-021-00603-8.

## Introduction

Since the early days of the pandemic, researchers have questioned whether or not COVID-19 exhibits pronounced temperature sensitivity. This question has remained important and largely unanswered (National Academies of Sciences, Engineering, Medicine 2020; Kanzawa et al. 2020; Kissler et al. 2020; Kerr et al. 2021). Kissler et al. (2020) examines medium and long-term management of the pandemic and assigns a prominent role to understanding how the temperature response profile (hereafter “TRP”) of COVID-19 might influence the pandemic’s progression in the United States (Carlson et al. 2020). This follows conventional wisdom regarding the strong seasonal pattern of influenza (Shaman et al. 2011; Barreca and Shimshack 2012), which helped mask COVID-19’s early U.S. ascent (Silverman et al. 2020). The rise in COVID-19 activity across the United States during the summer of 2020 quashed hopes that higher temperatures would dramatically reduce its transmission and reduced fears that colder fall temperatures would sharply increase it. Nevertheless, some modeling groups such as the University of Washington’s Institute for Health Metrics (UW-IHME), correctly assumed COVID-19 activity would increase as temperatures declined in the fall of 2020. In doing so, they used information from the U.S. influenza monitoring network (UW-IHME 2020,2021), designed to detect changes in respiratory ailments that had been previously shown to be predictive of the pandemic’s spring 2020 path (Silverman et al. 2020).

General problems with the U.S. COVID-19 statistical collection effort have often been noted (e.g., Schneider 2020) without directly touching on the question of the role of temperature. Daily state reports routinely bear little resemblance to the number of COVID-19-related deaths actually occurring on that date. This temporal data misalignment creates a substantial impediment to recovering any relationships with critical dependence on event timing. By reconstructing the death counts states reported daily – largely by substituting in retroactive corrections based on death certificate dates – we are able to reliably estimate the TRP for COVID-19 deaths.

We assemble over 4,500 state-level daily observations from April 16–July 15, 2020, after COVID-19 became reasonably well-established across the United States. We follow the environmental economics literature on estimating pollution and temperature related impacts on a range of health and other outcomes (Graff Zivin and Neidell 2013). As a novel virus, SARS-CoV-2 makes full implementation of the standard approach infeasible because multiple years of both spatially and temporally delineated data are not available. Importantly, we estimate the joint, not separate, effect of the temperature driven biological response by the virus and the behavioral response by its human hosts. While some might think that what we model here is almost exclusively a behavior reaction (e.g., spending more time indoors in closer contact with others in cold weather), that is unlikely to be a correct assumption. Influenza is known to be quite biologically responsive to temperature (and other weather variables) from animal studies and lab experiments that eliminate the social contact path (Lowen and Steel 2014; Moriyama et al. 2020). These mechanisms involve the interaction of temperature with the membrane of the virus, which influences its durability in the air and its ability to penetrate key cells in the nose. We take steps to ensure that some behavioral responses, such as the mandated and endogenous reductions in contact rates and use of physical measures to reduce transmission that helped blunt the first wave of the U.S. COVID-19 pandemic, have not been inadvertently incorporated into the estimate of the TRP. Our sample period was specifically chosen to be after the initial set of large-scale changes in these factors (often quite different across states) but before they later started to exhibit large state-specific time-varying differences in them.

We see the primary use of our TRP as providing a reliable estimate of the temperature transmission sensitivity function that can be imported into SEIR models and for COVID-related planning exercises. In mechanistic “forward” models of contagion (e.g., the SEIR family of models), a contagion’s amortized rate of transmission often collapses both biological and social factors into a single scalar parameter. The SEIR approach, originally put forward by Kermack and McKendrick (1927), is comprised of four differential equations which describe the motion of four groups known as compartments: Susceptible (can be infected), Exposed (to a carrier of the infection), Infected (can currently transmit the disease), and Removed (recovered from infection and now immune, dead, or vaccinated). SEIR models are often expressed in terms of population fractions, with Infected and Exposed compartments sometimes combined for simplification in theoretical work and expanded along demographic and spatial lines in empirical applications. Avery et al. (2020) provide an overview of this class of models from an economic perspective.

The single β_0_ parameter in the stylized SEIR model driving the amortized infection rate is very tightly linked to the well-known R_0_ measure of the expected number of Susceptibles a newly introduced infected individual is expected to transmit the disease to. The conceptual experiment defining R_0_ is straightforward—infect one randomly chosen individual in specific location (e.g., Chicago), without their knowledge, track and test all their contacts, and observe the number of other individuals this original index case directly infects. Repeating the experiment on statistically equivalent samples allows estimation of the expected number of people a randomly chosen index case infects, the formal definition of R_0_. Obviously, this experiment cannot and should not be performed in the current context. A variety of less reliable methods to estimate R_0_ from less than ideal data are employed instead (Anderson et al. 2020).

This conceptual experiment is ill-defined with respect to COVID-19 without a reference temperature. Our results suggest that if it were run at different temperatures, each temperature used would yield a systematically different R_0_. Our contribution here is not recognition that the reproduction rate of an organism can be temperature dependent (Anderson and May 1992) nor that initial estimates of R_0_ for COVID-19 in different contexts (i.e., Chinese cities) appear temperature related (Kahn et al. 2021). Rather, it is in showing how to reliably estimate the temperature responsiveness in R_0_ as one moves away from a reference temperature level, without decades of seasonal roundtrip data, and then operationally use that estimate to predict future patterns of this component of infection risk.

R_0_ is the ratio of the actual amortized rate of transmission, β_0_, divided by the inverse of the mean infection period, γ_0_. γ_0_ is often regarded as a biological constant, estimable independent of the usual approach of using the rate of exponential growth to determine R_0_. Its value leads to the recommended quarantine period of ten days for COVID-19, with infectiousness concentrated away from the edges of this period. Estimates of R_0_ (and equivalently β_0_ given an estimate for γ_0_) have tended to increase over time relative to the original Wuhan and Diamond Princess estimates due to reanalysis using better data, more sophisticated statistical models, alternative contexts such as prisons, and new variants (e.g., Huang et al. 2021; KhudaBukhsh 2021; Davies et al. 2021).

While β_0_ is often treated as a constant, this causes irreconcilable qualitative errors in model predictions. Assuming no reinfection and a well-mixed population, a SEIR model generates a single peak—a stylized fact that is at odds with this and several other pandemics. In contrast, a time varying version of β_0_, β_t_, can generate wavelike patterns. Shifts in β_t_ may be caused by endogenous or policy induced changes in effective contact rates. More infectious variants increase β_t_. Biological and social factors influenced by temperature and other environmental conditions are amortized into the β_t_ parameter.

With all these factors influencing the same deep SEIR model parameter, it is not surprising that researchers have found it difficult to reliably separate them. While the natural inclination of modelers is to try to consistently estimate all of the relevant parameters together in one logically coherent model, in the context of SEIR (and many other complex) models, this practice should be avoided where possible. To take a concrete example, there is no reason to estimate the average length of the infectious period, γ_0_, in a model when an estimate for that parameter can be reliably obtained from clinical studies where random samples of the population of interest are swabbed and tested daily. One of our main contributions is pinning down one of these parameters, the TRP, with sufficiently high precision that it can then be used to help identify the role played by other factors. Our modeling approach and the choices made with respect to it were guided by this objective.

Normalized by the estimate of γ_0_, β_t_ is often referred to as effective R_0_ and designated by R_t_ or R_e_. As the pandemic progresses, new, more infectious variants of COVID-19 have and will continue to emerge. Under the (testable) assumption that the relative infectiousness between COVID-19 variants is independent of temperature response, the β_t_, R_0_, and R_t_ functions are simply multiplied by the relative infectiousness of the variant. For β_t_ and R_t_, this one-to-one scaling also requires the assumption that neither policy makers nor the public endogenously changes their actions in response to the change in infectiousness. Since this later assumption is unlikely to hold, we will cast our results in terms of a time-varying expected R_0_, where the temporal variation is driven by the expected daily temperature at a particular location.

He et al. (2013) provides an analysis that attempts to sort out the role of several factors played in driving the three waves of the 1918 influenza pandemic in England and Wales, noting the difficulties in doing so. They find temperature to be the second most important factor, after behavioral responses, but note that the weekly aggregation of death counts and the relatively narrow temperature range in their data preclude confidence in finding that temperature was not the leading factor. We bring the tools of modern environmental economics to this task in the context of COVID-19 and assemble a much richer dataset.

Our work is based on a week-ahead forecasting model that accounts for most of the daily variation in COVID-19 related mortality (R^2^ = 0.97) and isolates COVID-19’s temperature response profile (*p* < 0.001). This TRP shows a 60% increase in week-to-week deaths when decreasing the temperature from 31 °C to 5 °C. The estimated TRP for new positives is much more pronounced—cases increase by almost 400% over the same temperature range.

As an example of using our TRP as the estimate of the temperature transmission sensitivity function in SEIR models, consider the Delta variant of COVID-19. Preliminary U.K. and U.S. estimates of Delta’s R_0_ put it in the range of [5.0–9.5] (Gallager 2021; Washington Post 2021), putting it on par with chickenpox. We conservatively assume that the low end of this range represents the R_0_ for the average 31 °C U.S. mid-July temperature. If the Delta variant has a substantively similar positive case TRP to that of the original virus, and the two sources of increasing infectiousness are multiplicative (as suggested by the structure of a simple SEIR model), then a frightening degree of transmission amplification is possible. As temperatures move toward 5 °C in many places, a temperature-referenced R_0_ defined using our TRP moves the Delta variant from chickenpox-like transmission potential to the transmission potential of measles (R_0_ 12–18), one of the most infectious pathogens known. We hope the modeling community can rapidly use our TRPs to produce more accurate longer-term forecasts to help guide pandemic planning and response efforts. A detailed discussion of creating location-specific, temperature-referenced, time-varying R_0_ series is provided in the Supplemental Material.

### Prior Efforts

COVID-19’s temperature response profile (TRP) proved elusive to early attempts at measurement. The now well-accepted approach for estimating influenza’s TRP is epitomized by Barreca and Shimshack (2012), which draws heavily on the literature modeling climate impacts on human populations (Graff Zivin and Neidell 2013; Auffhammer et al. 2013; Hsiang 2016). Under this approach, a panel data set of political entities, such as countries or their political subregions (e.g., states, counties), is assembled and the outcome of interest is observed across a long time horizon (e.g., a 20-year period). The ability to employ fixed-effect indicator variables to correct for time-invariant differences between political jurisdictions, coupled with the use of short-run weather variability, provides statistical identification of a variety of impact response functions.

Prior research has three important limitations. First, cross-sectional data cannot statistically identify the desired function without making the implausible assumption that all confounding variables have been adequately controlled for. Routinely updated time-series models slowly incorporate environmental conditions, via the lags of their dependent variable, into their forecasts without ever isolating them. Short panel datasets, where the stimulus of interest has a limited range (temperature, humidity, UV light, air pollution in each location), often lack the statistical power to pin down such response functions. Consequently, estimated response functions frequently have conflicting signs on key variables or are fragile in the sense that statistical significance is gained or lost when time trends or demographic variables are added to models (Briz-Redón and Serrano-Aroca 2020; Ding et al. 2021; Jamshidi et al. 2020; Jüni et al. 2020; Kerr et al. 2021; Mecenas et al. 2020; Pedrosa 2020; Xu et al. 2020).

Second, early work (often using Chinese or cross-country data) focused on predicting the speed at which the pandemic spread in different locations using derivative statistics such as growth rates or R_0_ (Wang et al. 2020; Carleton et al. 2021). These works pointed to the 0–10 °C temperature range as being most conducive to spreading COVID-19, with a possible humidity effect (Wang et al. 2020; Sajadi et al. 2020; Ficetola and Rubolini 2020). However, current interest is focused on situations where COVID-19 is spatially well-seeded and where β_t_ can change with actions like changing state-level COVID-19 restrictions.

Third, the quality and comparability of reported COVID-19 statistics is often suspect, particularly from the early phase of the pandemic. We move past this initial period to an observational window where both reporting the key statistics of the pandemic and the initial behavioral response to it had largely stabilized. A different problem now dominates – temporal mismatches between when an event (e.g., a COVID-19-related death) was reported and the weather variables potentially influencing that event (Hsiang 2016). When reported event dates significantly differ from actual event dates, the resulting measurement error can overwhelm standard sources of biological variation, such as individual-level differences in incubation periods.

### Correcting State-Level COVID-19 Statistics and Why It Matters

Our ability to isolate the TRP for COVID-19 related deaths stems largely from our reconstruction of state-level COVID-19 data. Replacing the daily death counts initially reported by states with the revised daily counts based on actual death certificate date is the most important of these steps. We are able to do this for 65% of the states representing almost 80% of the U.S. population. For many states, a simple OLS regression of death counts by death certificate date on the death counts the state originally reported yields an R^2^ substantially less than 0.5.

While there is no doubt that there are still some misclassified deaths in this data source, such errors are thought to be greatly diminished by our sample period due to the greater recognition of COVID’s many avenues of attack and increased testing capacity. There is some medical discretion as to whether COVID-19 is listed as an underlying or a contributing cause of death. This is irrelevant for our purposes since we follow the standard practice of defining COVID-19-related deaths as including both categories. With respect to the death certificate date, in the U.S. it is a criminal offense to falsify death certificate dates due to their use for life insurance purposes. There is also little incentive for state officials to do so since they have considerable discretion over “when” information on COVID-related deaths is made public.

When death certificate date data is not available, we use the retroactively corrected data series that several states have produced. This type of data generally rectifies many obvious initial reporting errors and provides a more consistent treatment of initial “probable” COVID-19-related deaths across states. In these and the other remaining (mostly small) states, we also correct other implausible data reports such as implicit negative daily death counts and zero counts on one day followed by a clear double-count the following day. The other main issue involves states reporting a batch of previously unreported deaths (typically from nursing homes) on a particular day, with varying degrees of information on when those deaths might have actually occurred. The Supplemental Material provides details on the construction of our COVID-related variables.

**Fig. 1:**
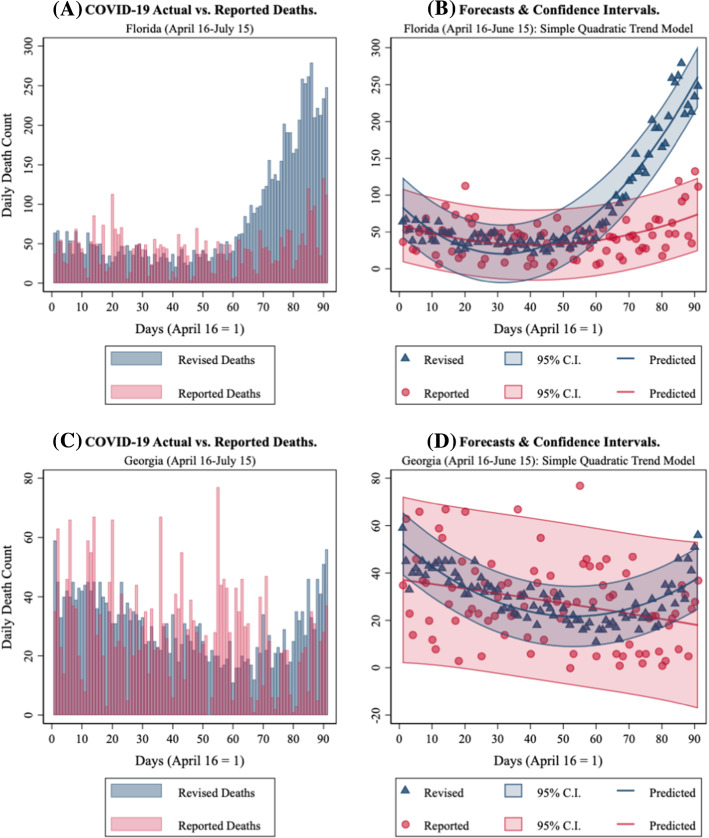
COVID-19 Deaths in Florida and Georgia. April 16-July 15, 2020. **A** Florida Revised vs. Reported. **B** Florida quadratic time trend forecasts and confidence intervals. **C** Georgia Revised vs. Reported. **D** Georgia quadratic time trend forecasts and confidence intervals

Figure 1 displays how repairing daily state-level COVID-19 death counts impacts modeling. Panel (A) shows the death counts originally reported by Florida [as archived by the COVID Tracking Project (CTP) (CTPDailyDead_it_)] in red with the “Revised” death counts by death certificate date overlaid in blue. Revised curves follow the general shape predicted by epidemiological models, whereas curves based on the originally reported counts are characterized by large spikes and very pronounced day-of-the-week patterns, neither feature predicted by biological models. Panel (B) shows the two implications of these results. First, the confidence interval from fitting a simple quadratic trend model is dramatically larger using the death counts Florida originally reported death counts than with the same model fit using death counts based on the death certificate dates. Second, this model is slow to pick up the sharp rise in Florida deaths since the originally reported counts temporally lag the revised counts. Results for Georgia are displayed in Fig. 1, Panels (C) and (D). They show that state’s originally reported death counts led to both missing a downturn and an upturn, while supporting an incorrect story of slow progress. Many other states show similar patterns.

**Fig. 2:**
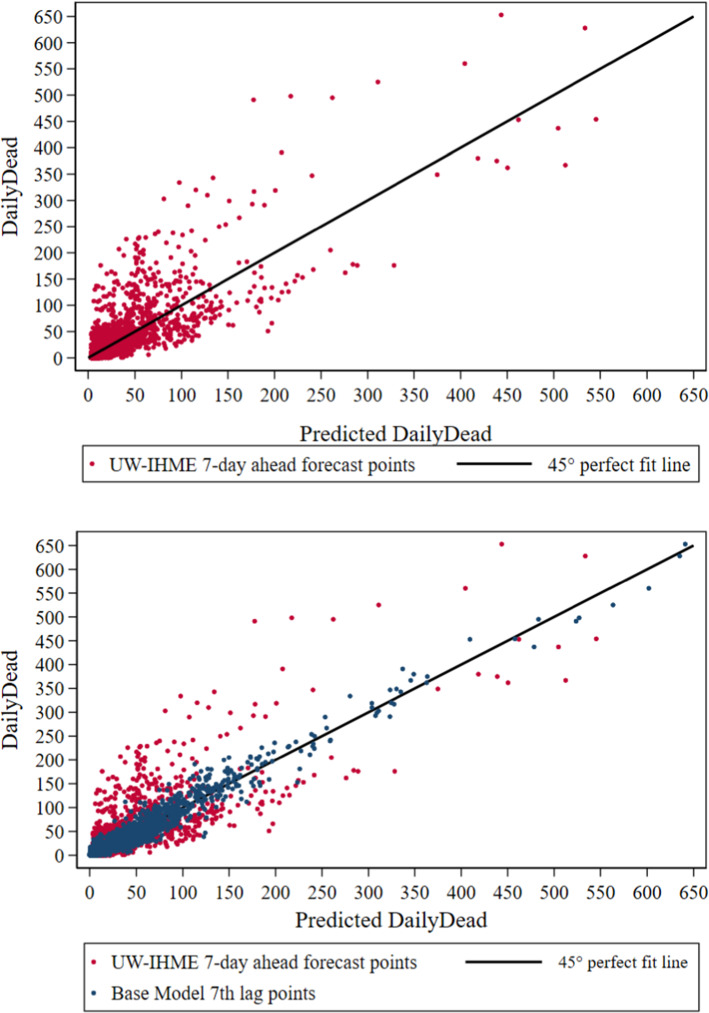
DailyDead_it_ vs. predicted values using information available 7 days earlier. U.S. States April 16-July 15. **A** UW-IHME forecasts (R^2^ = .66). **B** Eq. 1 base model predictions (R^2^ = .97)

Figure 2 Panel (A) shows the week-ahead forecast from the University of Washington’s Institute of Health Metrics and Evaluation (UW-IHME 2020) plotted against our model’s DailyDead_it_ at the state level for the entire United States over the three-month period we examine. This model explains a reasonable amount of the variance, reflected in the R^2^ of 0.66 obtained by regressing our Revised death counts on the UW-IHME forecasts. Substituting the week-ahead forecasts from other heavily used forecasting models results in a similar impression.

Figure 2 Panel (B) plots predicted DailyDead_it_ from the forecasting model (Eq. 1) presented in the next section, which only uses information available a week earlier. This regression’s R^2^ of 0.97 is reflected in the tight scatter plot. Since our model is not heavily parameterized, by far the main source of the improvement in the fraction of variance explained is estimating the model using our temporally aligned death counts (Table S4, Model 21; figures and tables in the Supplement Material are denoted by the prefix “S”). The performance of our data correction effort with providing estimates for all four pairings of our Revised versus the originally Reported death counts are provided in Table S4. It is worth noting that even prediction of current Reported death counts is improved by using our lagged Revised counts. Our specification, allowing for temperature amplification, further reduces the root mean square error by a little over 30% relative to just using the revised data. Overall with the combination of better data and our model, we have moved away from the original situation, where one of the major forecasting models being used by government officials across the U.S. had a week-ahead root mean square prediction error more than 20% larger than the state-level daily mean death count of 22 during our sample period (~ 1100 nationally), to one where that standard prediction accuracy measure is less than 40% of it.

### Modeling Approach

We use a production function approach that requires substantially more data than the typical SEIR modeling approaches that are applied to data in the early stage of a pandemic. Our model’s greater flexibility is particularly important once an outbreak has reached a point where, after initial interventions, positive cases and death counts can ebb and flow in both directions rather than exponentially increase. During our sample period, Gu (2020) estimates that the U.S. average R_t_ was 1.02, with a minimum value of 0.83 and a maximum of 1.31.

Conceptually, we start with a time-lagged infection pool that represents the seeds of the current pandemic trajectory that influence the output: the number of deaths observed today. Our main specification uses past death counts, which are stochastic realizations of the relevant infection pool, as a proxy for the size and composition of that pool. This infection pool reflects a multitude of factors, ranging from a state’s demographic composition to its social network structure rather than just the number of currently infected (e.g., quarantined infected are not doing much to spread the disease). Our model uses state-level fixed effects to control for the factors that are effectively constant over the time period examined such as population size, demographic composition, the quality of its public health infrastructure and transportation networks, and average differences in factors such as variables and mobility of its population. We use a simple quadratic time trend in our main specification to accommodate a set of factors (e.g., facemask adherence and falling case fatality rates) that drove down death counts in the early part of our sample period (before their effect appears to flatten out). Conceptually, small changes in exogenous factors like temperature can rock this system back and forth, allowing their influence to be uncovered.

A set of state-specific trends is not separately identified in our model. However, we show that our main TRP result is robust to including controls for the most obvious (non-temperature) potential source for such trends: state-specific trends in contact rates. We proxy for these by using the standard dates for state-level shelter in place and reopening dates as well as changes in observed state-level mobility data.

While the decision to use death counts rather than test-positive cases is broadly consistent with most modeling efforts of this time-period, this issue deserves more discussion here because our main objective is to uncover COVID’s TRP rather than to put forward a pure forecasting effort. Logically, the causal relationship should be between NewPositives_it_ and temperature. The time link between them should also be shorter than for their DailyDead_it-k_ counterpart. If those transmitting the disease have higher contact rates than those dying of it, then the TRP for positive cases is expected to be more responsive than the TRP for deaths. With deaths concentrated among nursing home residents, this generates a clear prediction that our results confirm: the positive TRP is more responsive to temperature than the one for death counts.

Even if one wants to predict DailyDead_it_, NewPositives_it-k_ might be a better predictor than DailyDead_it-k_. However, what makes the reported NewPositives_it-k_ measure more complicated and potentially less reliable to work with than DailyDead_it-k_ is that it does not represent all or even almost all the positive cases in state i on date t-k. That is because there is no universally administered daily test. Observing all positive cases, as we do with the death counts, is not actually needed. Large-scale routine surveillance testing could have been implemented in each state using high quality probability sampling to provide estimates of such quantities. However (and somewhat surprisingly), such surveys were not deployed during our sample period. As such, observed positives through testing are not a random sample of positive cases. A sizeable fraction of positive cases go untested as they are mild, and another large fraction of cases (15% to 70%, CDC, 2021) involve asymptomatic individuals. There are also known selection issues with respect to testing on demographic variables. The best we can hope for with currently available data on positive cases is to observe a stable fraction of them conditional on testing data. We employ such a specification in our empirical modeling of the relationship between positive cases and temperature. Many of the same temporal alignment issues that arose with death counts characterize positive test results, with many testing delays being non-random in nature. This problem could be addressed retrospectively (and going forward), if test results were also reported by day of administration rather than only the date returned from a lab, but very few states have done this for our sample period.

We use the three-month period of April 16-July 15. In doing so, we avoid the chaos of the early first wave where a substantial fraction of positive cases and deaths were not recognized due to a lack of adequate testing capacity and initial knowledge concerning the full range of COVID-19’s clinical manifestations. Many early COVID-19 deaths were misclassified as influenza (Silverman, 2020). Furthermore, the usual rationale advanced for using death counts – the presumption that death counts due to the new disease of interest are always observed – is demonstrably false (Salerno et al. 2020; Weinberger et al. 2020).

Our time window allows examination of the *i* = 1,…,51 U.S. states (including DC) over a three-month period. This window means that we are mainly looking at sustained community transmission rather than how the major initial seeding events, e.g., the dispersion of returning tourists from Italy and other early European hotspots, played out. We effectively start to make use of observable outcomes, deaths, positive cases, and tests on April 9th, because we often use a one-week lag of the variables of interest. Our time variable, *t* = 1, …, 137 (denoted in days) starts with 1 on March 1st, the approximate date individual states started reporting COVID statistics.

Our time window excludes the fall/winter 2020–2021 period, where facemask adherence and social distancing became increasingly politicized and hence divergent across states, and mixtures of mutated COVID-19 variants known to differ by transmission efficiency started to be introduced (Li 2020). While there is no current evidence that COVID-19 variants of interest have evolved different temperature profiles, this remains an important open question. Our time window allows us to observe cold-to-warm TRP direction. To a first approximation, it is reasonable to expect the TRP to be similar going the other direction, but plausible behavioral and biological mechanisms suggest some caution in making that assumption.

Our main statistical model isolates COVID-19’s TRP with respect to state-level daily death counts (DailyDead_it_). We also look at new positive test counts (NewPositives_it_). Any reasonable TRP specification should allow for the possibility of non-responsiveness (i.e., zero temperature dependence), and a test of that null hypothesis. Daily exogenous variation in temperature on any specific day should be the source of statistical information for identifying the TRP of interest. Intuitively, the TRP is being statistically identified by having days where the lagged infection pool variable is of approximately the same magnitude, but a range of different temperatures is observed. The number of such comparable days is substantially increased by the introduction of controls for conditions that remain fixed across states and a quadratic national time trend.

Statistical modeling issues revolve around functional forms and the specification of the relevant lag structure. The slow-moving nature of systematic changes in temperature over time implies that, over a short time horizon, temperature is unlikely to be the primary driving force behind DailyDead_it_ and NewPositives_it_. Over longer time periods, the TRP of a virus can be a major factor that helps to steer the path of a pandemic, an issue we examine later (see Fig. 8).

We use a multiplicative scaling function for temperature. This feature of our model incorporates the logic that sufficiently lagged temperature by itself cannot generate changes in DailyDead_it_, i.e., adverse changes in temperature do not predict an appreciable increase in COVID-19 deaths in small states when their lagged infection pool indicator is zero. Deaths where both heat and COVID-19 are listed as underlying or contributing causes are included in our dependent variable. The logic here is that the temperature that contributes to a heat-related death occurs after the temperature that contributes to a COVID-19-related death (i.e., the temperature at the time of transmission). This may contribute to our finding that COVID-19’s TRP appears relatively more sensitive to temperature changes at the higher end of our temperature range than influenza (Barreca and Shimshack 2012).

Because those dying on day *t* became infected over an extended period of time rather than on a single day, some means of representing temperature in this setting is required. The two options (due to the very strong correlation between closely adjacent MaxTemp_it-k_) are a distributed lag framework that imposes structure on a set of MaxTemp_it-k_, or parameterizing the scaling function as the product of individual scaling functions, each with a different lag of MaxTemp_it-k_. We chose the second approach. Parsimonious distributed lag models tend to impose the restriction that lags are linearly decreasing or increasing in lag length, a feature unlikely to characterize our situation.

The main scaling function we use is the product of two standard logistic functions, 1/(1 + EXP(X)^ϓ^), where X is the variable of interest, and ϓ is the single estimated parameter. This function converges to a constant as ϓ goes to zero. For X, we consider both MaxTemp_it-k_ and LogMaxTemp_it-k_ as the stimulus variable. Mean temperature provides an almost identical fit and using minimum temperature provides a slightly worse fit.

We also consider another commonly used scaling ratio function, X/(X + ψ). An estimate of ψ > 0 results in smaller values of X being scaled up more than large values of X and an estimate of ψ < 0 results in larger values of X being scaled up more than smaller values of X. An estimate of ψ = 0 results in a function exhibiting no temperature responsiveness. This function can also be used with either LogMaxTemp_it-k_ or MaxTemp_it-k_. This ratio scaling function is well behaved provided the estimate of -ψ is bounded sufficiently away from MIN(X), which appears not to be an issue in the situations we examine. This ratio scaling function and the logistic scaling function introduced earlier both impose a key identifying restriction, taken from what is known about influenza (Barreca and Shimshack 2012), the TRP should be is a smooth, weakly, monotonic function over the temperature range examined.

The start date of our sample period leads us to restrict attention to conditions where MaxTemp_it_ ≥ 5 °C. Below this temperature level, data is relatively scarce during our sample period and concentrated in a few sparsely populated states such as Alaska and Montana. If death and positive case TRPs behave like influenza (Barreca and Shimshack 2012) then they will increase from 5 °C until a few degrees below freezing. However, for COVID-19 this remains an unknown. Much below 5 °C the assumption that our TRP is a smooth, weakly monotonic process may no longer hold. Our estimated TRPs are normalized to 100 at 31 °C (~ 88°F) – the U.S. population-weighted average for our sample’s last (mid-July) week – to aid interpretability.

## Model Specification

Formally, our base model for a U.S. state *i* on day *t* is given by:

1 $$ \begin{aligned} {\text{DailyDead}}_{{{\text{it}}}} = & {\text{ EXP}}\left( {\Sigma_{{\text{i}}} {\text{StateIndicator}}_{{\text{i}}} + \, \alpha_{1} {\text{LogDays}}_{t} + \, \alpha_{2} {\text{LogDays}}_{t}^{2} + \, \alpha_{3} {\text{LogDailyDead}}_{{{\text{it}} - 7}} } \right) \\ & *\left( {1/\left( {1 \, + {\text{ EXP}}\left( {{\text{LogMaxTemp}}_{{{\text{it}} - 7}} } \right)^{{\gamma 1}} } \right)} \right)*\left( {1/\left( {1 \, + {\text{ EXP}}\left( {{\text{LogMaxTemp}}_{{{\text{it}} - 14}} } \right)^{{\upgamma 2}} } \right)} \right) \\ & + \, \varepsilon_{{{\text{it}}}} , \\ \end{aligned} $$

where estimated coefficients for the StateIndicator_i_, and the Greek letter parameters minimize the sum of the square of estimated error terms. The NewPositives_it_ model is similarly structured. The additive error term necessitates using nonlinear least squares to solve the model (Goldfeld and Quandt 1970; Wooldridge 2010) but decouples conditional mean estimates from the estimated error component.

The logic of this model is that it is a product of two components. The first produces expected current-period deaths as a function of past observed deaths (and other covariates) averaged over observed temperatures. The second allows expected DailyDead_it_ to (potentially) vary with past values of MaxTemp_it-k_.

The terms inside the first component are the infection pool proxy, DailyDead_it-7_, state-level fixed effect indicators, StateIndicator_i,_ and a quadratic time trend in Days_t_ (*t* = 1, …, 137; initialized to March 1 to aid interpretability). The StateIndicator_i_ captures the influence of a wide range of variables that remain constant over the period examined. The time trend variables pick up the decline in the case fatality rate and the average effect over time of initial lockdowns, social distancing, and state re-openings. The first component is exponentiated to incorporate the restriction that expected DailyDead_it_ cannot be negative and should be positive if COVID-19 transmission is active anywhere in the set of connected units examined. Commensurately, we use LogDailyDead_it-k_ and LogDays_t_ as regressors, so this component can be interpreted as a log–log regression model with state-level fixed effects and a quadratic time trend. While it may seem natural to use more than one lag of DailyDead_it-k_, this should be avoided. Adjacent lags are very highly correlated (0.98, contrasted with 0.61 for the originally reported death counts), as one would expect from a pandemic once a new virus is well-established.

The second component is the logistic function that scales predicted DailyDead_it_ up or down with changes in MaxTemp_it-k_. Deaths on specific days are the result of infections propagated over earlier days. We use LogMaxTemp_it-7_ and LogMaxTemp_it-14_ (one and two weeks in the past respectively) to roughly encompass the period when temperature could have influenced COVID infectiousness.

Our choice of the 7th lag is motivated by the CDC’s (2021) scenario planning estimate that it typically takes six days from exposure to symptoms. Conceptually, the first lag should be a bit longer than this CDC estimate, since shorter lags are highly correlated with it. While most deaths would occur later, sudden COVID-19-related cardiac deaths are common. Our choice of the 14th lag is influenced by CDC-recommended ten-day quarantine period (where transmission risk is concentrated in the early middle part of this period). Coupled with the distribution of times from infection to symptom, suggests putting the second lag about a week after the first. The strong correlation between temperatures on adjacent days suggests our two temperature values are effectively spanning a period from about 3 days before *t* to 18 days after *t*.

Clearly, there are some deaths that occur substantially later. As this duration of time gets longer, the exact date of a death is likely to be strongly influenced by local attitudes toward mechanical life support. The average flow of such deaths, which are no longer well linked to temperature, and augmentations of the state’s infection pool with positives from outside the state getting incorporated into the state-level fixed effects. The implication of this for different states in terms of generating future deaths is displayed in Fig. 5. Deviations from these average flows contribute to heteroscedasticity in our error component.

Model results are reasonably robust to small shifts in temperature lag positions, with two caveats. First, the 7^th^ lag is a natural one to use; many people’s lives follow a typical weekly pattern of contacts and activities, and administrative reporting procedures often have a day-of-the-week pattern. Second, lags too far back should be and are insignificant. Our results (Table 1) show the temperature parameters on the two lagged variables are sizeable, significant (*p* < 0.001), and with respect to their magnitudes, statistically indistinguishable. This last result is an interesting property of our estimated TRP that warrants further investigation. Using the same 7th and 14th temperature lags with positive cases as the dependent variable, we find the 7th lag to be large and the 14th lag small. Since the time link should be shorter for positives, we move the 14th lag to the other side of the 7th lag. We now find the temperature parameter on the 2nd lag large and of similar magnitude to the 7th lag. This result is consistent with individuals showing severe symptoms, typically in a hospital context and getting their tests quickly returned, while other individuals being tested experience a wider distribution of return dates.

**Table 1:** Slope coefficient estimates for Main DailyDead_it_ model

Variable	Coefficient	Robust S.E	t-statistic	*p*-value
LogDays_t_	-10.3772	1.4614	− 7.10	0.000
LogDays_t_*LogDays_t_	1.1834	0.1696	6.98	0.000
LogDailyDead_it-7_	0.8670	0.0277	31.28	0.000
LogMaxTemp_it-7_	0.3110	0.0448	6.95	0.000
LogMaxTemp_it-14_	0.2945	0.0516	5.71	0.000

We investigate [Tables S3 and S6] the sensitivity of our results to a range of alternative specifications, e.g., different infection pool indicators, alternative temperature scaling functions, use of a population-centroid weighted temperature series, adding absolute humidity, relative humidity, ultraviolet radiation, the inclusion of shelter-in-place/reopening orders, controlling for mobility, use of lagged cumulative death counts, and the use of the death counts states originally reported. We also advance a version of our base model where the dependent variable and infection pool indicator are weekly rolling averages that update daily. A detailed discussion of this model is provided in the Supplemental Material. Its specification tends to average out some reporting errors and is consistent with biologically driven heterogeneity in the duration between exposure and symptom onset being potentially important.

### Data

Our analysis uses three main types of data:

1. COVID-19 statistics for state-level death counts, positive cases, and tests, using The COVID Tracking Project (CTP; covidtracking.com) as our base information source.
2. Temperature data from the U.S. National Weather Service Integrated Surface Database.
3. State-level indicators and time variables.

As described in an earlier section and extensively documented in the Supplemental Material, we undertook very extensive repairs of the COVID-19 data reported daily by states, particularly those involving death counts. A dominant feature of this data is that there are substantial differences between when many of these events actually occurred and what states initially reported. It is not uncommon to see states include deaths from several weeks prior in any given day’s count.

For each state, weather variables are obtained for the airport with the highest volume of commercial traffic. We focus on the maximum daily temperature [details in Supplemental Material]. State-level aggregation requires taking weather data from a single station. However, measurement error induced by this compromise is likely to be less than one might think. Many states are spatially small or have a single, concentrated metropolitan area (e.g., Chicago, Illinois). In spatially large states with large populations, most people typically live within reasonable proximity to the largest airport. Even in Texas, most people live along the corridor between Dallas (DFW is our representative airport) and Houston. As a result, over 60% of the American population lives within 300 km of their state’s main airport. Further, major airports have been identified as a major spatial driver of spreading infectious respiratory diseases, generally (Browne et al. 2016), and COVID-19 specifically (Chokshi et al. 2021), with positive cases concentrated in their associated metropolitan areas and emanating outward from those areas. We examine the robustness of our decision to use main airport temperature measure by looking at estimates based on the use of an alternative temperature series constructed for the population-weighted centroid in each state.

### Daily Dead Model Results

Parameter estimates for the slope coefficients for Eq. (1) and summary statistics estimated using non-linear least squares are provided in Table 1. (Table S1 displays the full model including the state-level fixed effects). All estimated parameters are statistically significant at the p < 0.001 level, using robust standard errors clustered at the state-level. LogDailyDead_it-7_ is the dominant predictor. Its estimated coefficient, 0.8670 (t = 31.28), has a standard elasticity interpretation.

Table 2 shows that just over two thirds of the variation in state-level fixed effects are explainable by a simple OLS regression of minimum expected death counts on a small set of three state-level demographics taken from the U.S. Census Bureau (LogPopulation, %Black, and %Hispanic). States where the actual death certificate dates were available have considerably smaller prediction errors (p < 0.001) (Model 2 in Table S6).

**Table 2:** Model predicting statebase_i_ taken from DailyDead_it_ model

Variable	Coefficient	Robust S.E	t-statistic	*p*-value
Constant	− 2.3671	0.4478	− 5.29	0.000
LogPopulation	0.3763	0.0665	5.66	0.000
Log%Black	0.1208	0.0531	2.28	0.027
Log%Hispanic	0.1621	0.0483	3.36	0.002
R^2^	0.6649			
Root MSE	0.3949			
Observations	51			

**Fig. 3:**
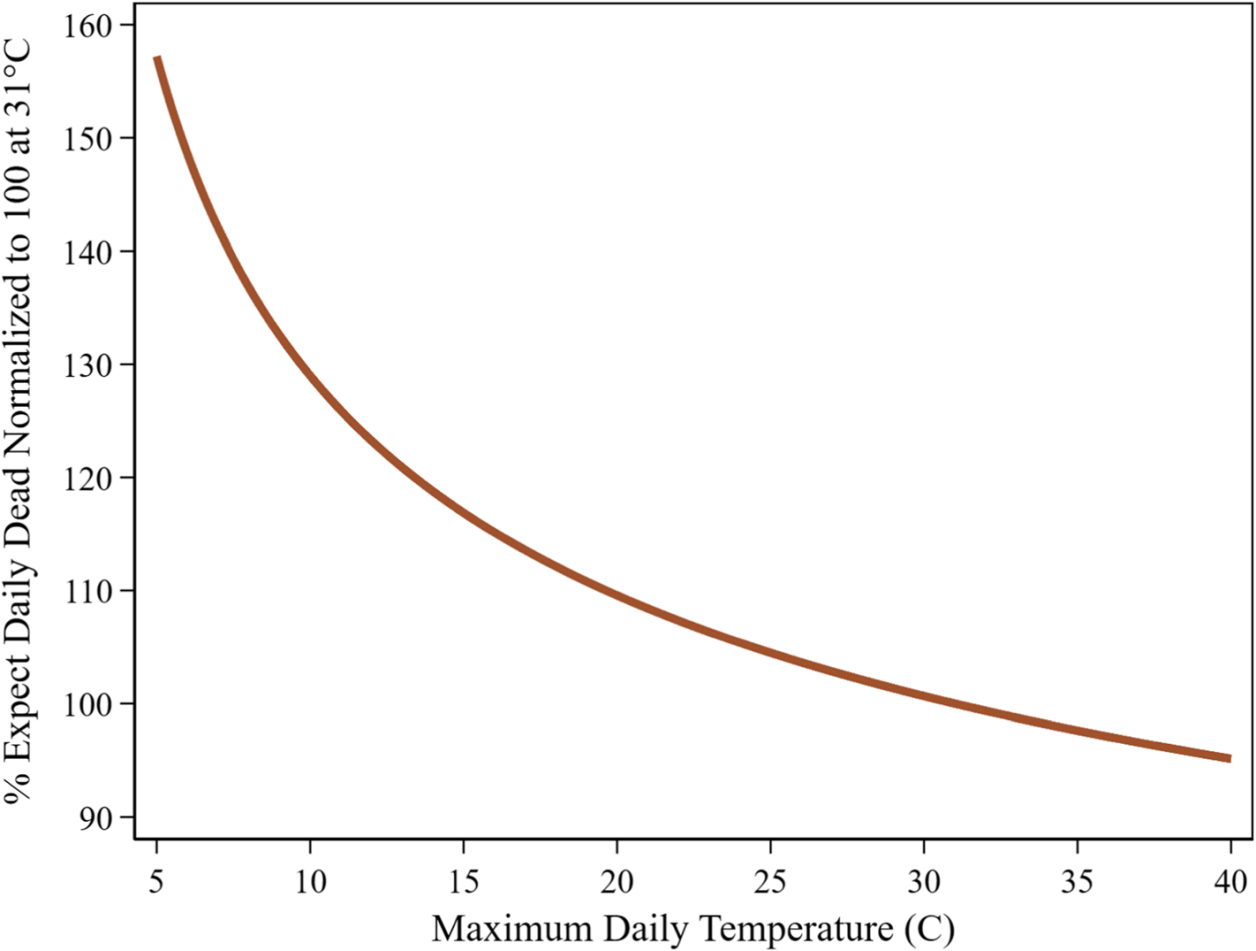
COVID-19 daily dead temperature response profile. U.S. States: April 16-July 15. Temperatures for 7th & 14th lags set equal

Figure 3 shows the TRP implied by the parameter estimates from Eq. (1) for the two MaxTemp_it_ lags in the logistic scaling function (details on TRPs construction are contained in the Supplemental Material). The vertical axis represents expected DailyDead_it_ at each temperature value, when both lags are set to the same temperature and the TRP is normalized to 100 at 31 °C. It reaches a maximum value of 160 at 5 °C. This TRP has a simple interpretation. If the predicted number of deaths at 31 °C is X, then at 5 °C the predicted number of deaths is 1.6X, where this logic and our estimated curve will provide the appropriate scale factor for converting between any two temperature values. A limitation of the TRP, as plotted in Fig. 3, is that to obtain a simple two dimension representation, the two lagged temperature variables were set to the same common value. Figure S1 provides the contour plot implied by our base model where the two MaxTemp_it-k_ lag values are allowed to vary independently.

#### Controlling for Potential Confounding Factors

We do not want TRP estimates to be confounded by other factors changing over time. These include state-mandated shelter-in-place orders, reopening plans, endogenous changes in contact rates, changing propensity to wear face masks, and changing case fatality rates. Since we are agnostic as to the mechanism, the most straightforward specification to consider first is polynomial time trend. Our base Eq. (1) specification uses a quadratic national time trend. Exploring this further, we find linear and quadratic, but a likelihood ratio test (*p* = 0.1264) suggest higher order terms are not needed. The quadratic trend for predicting DailyDead_it_ falls sharply until the end of May, after which it remains fairly flat before turning up very slowly at the end of our sample period. The usual caveat with a time trend from any short-run forecasting model like ours, is that any trend may not continue well into the future. This is particularly true when the underlying phenomena are known to be governed by a SEIR model. At some point, the growth path of the virus has to slow by an increasing fraction of the population in the Removed compartment.

Anecdotal evidence suggests our quadratic trend may be due to declining case fatality rates. This would be problematic if there is a large state-specific component to this. We can look at this issue to some extent by including a polynomial series in state-level per capita cumulative death counts, LogTotalDead_it-7_. Table S6, Model 14 shows that a quadratic series is at best marginally significant, and Model 15 shows that moving to a fourth-order polynomial series adds little. These results are suggestive of a weak initial local learning-by-doing effect that helps drives down the case fatality rate, but one that quickly dissipates and should be absorbed into the state’s lagged death count variable. Adding LogTotalDead_it-7_ effectively converts state-fixed effects into a dynamic object by coupling them with a time-varying component (see Supplemental Material for a discussion). If the size of the Removed fraction had grown large enough in some places to be substantially slowing transmission during our sample period, the polynomial in LogTotalDead_it-7_, should be decreasing at an increasing rate. We find it decreasing at a decreasing rate.

There is no state-level time series on the propensity to wear a facemask. The New York Times (Katz et al. 2020) undertook a very large, detailed, survey-based picture of facemask usage in July which showed considerable spatial variation. This should be picked up in our state-fixed effects. Other surveys paint a national picture of facemask adherence, rising sharply at first and then slowing. Average levels differ by state in a manner consistent with political orientation. Again, this story is consistent with our quadratic trend.

We now turn to state-mandated policies that influence contact rates, specifically at models which add (a) the number of days since a state issued a mandatory shelter-in-place order and (b) the number of days since a state started to formally reopen its economy. Results are in Table S6 (Model 8). The coefficients on these two variables are small and insignificant. The two LogMaxTemp_it-k_ parameters are statistically indistinguishable from those in our base specification.

This result might seem puzzling until one realizes these two sets of time-related variables are highly correlated. Dropping the quadratic trend (Model 9 in Table S6) provides a different picture. The earlier a state’s shelter-in-place order was issued, the lower the predicted death count (*p* = 0.092). The earlier a state started to reopen, the higher the predicted death count (*p* = 0.015). We are reluctant to provide any substantive interpretation of this result but note that the estimated temperature coefficients in this model fall by less than 20% on average. Papers that try to determine the role of state actions and the behavior they are intended to influence show the need to extensively model both state and local orders, and they incorporate detailed spatially disaggregated mobility data in order to differentiate the effects of these government mandates from endogenous social distancing by the public that often occurs before these mandates (Courtemanche et al. 2020; Goolsbee et al. 2020; Chetty et al. 2020). That task is beyond the scope of this paper.

We can, however, look at the impact of adding (Table S6, Model 27) the now standard state-level SafeGraph cell phone mobility data as a proxy for endogenous changes in contact rates to our base specification. This variable is cast relative to each state’s average weekly mobility to avoid simply picking up population differences, with the normalization week being the first week of March. This is well before the first state shelter-in-place order by California on March 19. Use of this variable requires choosing a lag length consistent with the temperature lags (7 and 14 days) in our base specification. This model’s R^2^ and the significance of this mobility variable are maximized at a lag of length of 11 days (*p* = 0.145). The temperature parameters increase, on average, by a little over 5% relative to those estimated for Eq. (1) and are statistically indistinguishable.

#### Understanding Implications of the Base Model Specification

**Fig. 4:**
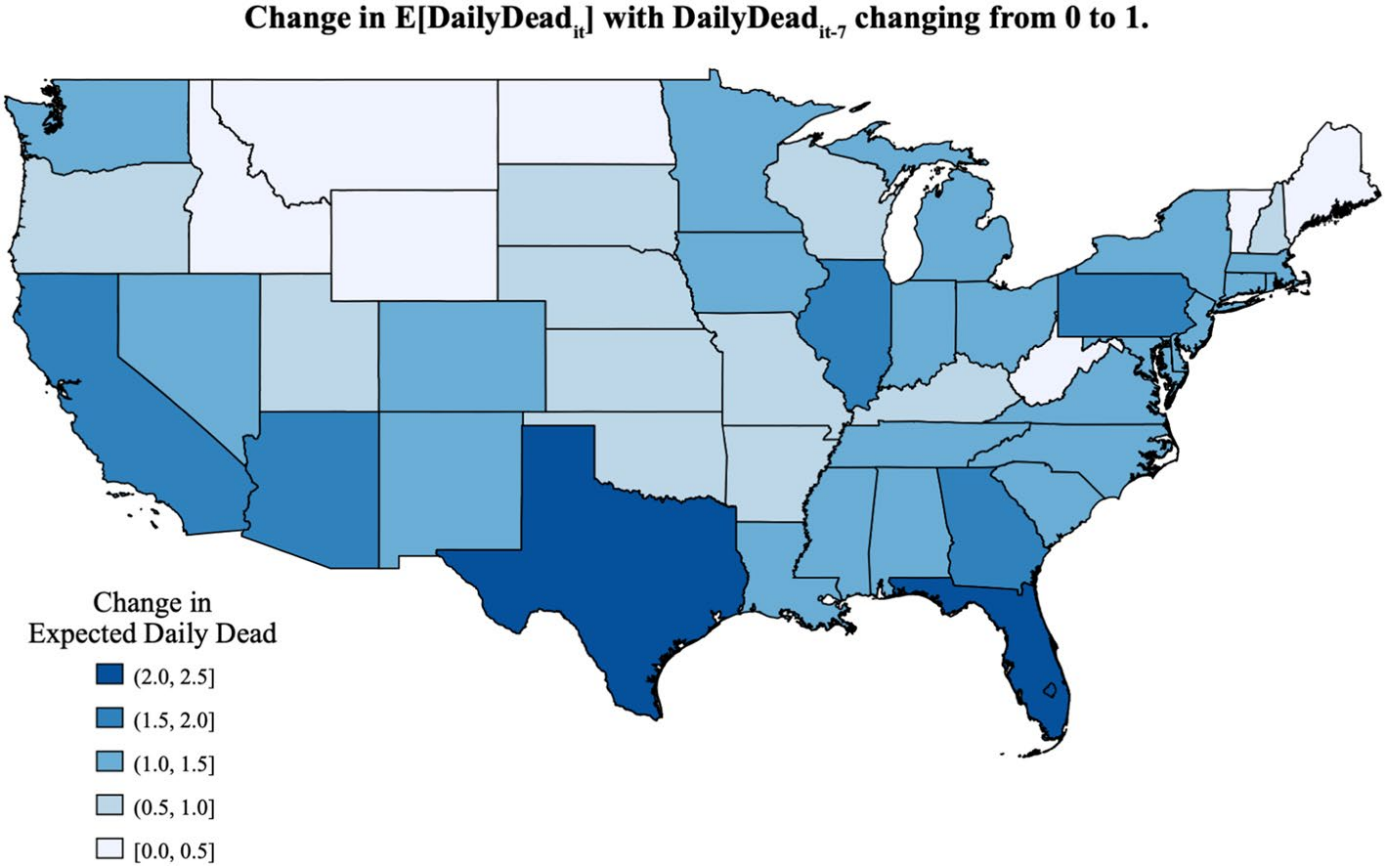
Expected DailyDead_it_ for U.S. continental states as DailyDead_it-7_ moves from 0 to 1, when the date is set to July 15 and the two lagged MaxTemp_it-k_ are set to 31 °C

Differentiating Eq. (1) with respect to DailyDead_it-7_ produces a measure of how the expected DailyDead_it_ increases from one more death in DailyDead_it-7_. Setting *t* and MaxTemp_it-k_ to their chosen values yields [TRP*α_3_*EXP(StateBase_it_)]/(DailyDead_it-7_)$_{({{1-{\alpha}}_3})}$, where the TRP directly scales the elasticity parameter, α_3_, on LogDailyDead_it-7_, and StateBase_it_ is the temporally varying sum of the fixed StateIndicator_i_ and the quadratic time trend. As an example, for Arizona on July 15 (StateBase_it_ = 2.1743, MaxTemp_it-7_ = 43.3, MaxTemp_it-14_ = 40.0 and the corresponding non-normalized TRP = 0.0359), changing DailyDead_it-7_ from 79 to 80 is predicted to increase the expected DailyDead_it_ by 1.9179. This can be seen as a variant of an effective R_0_ calculation. The map in Fig. 4 displays another variant of this calculation for states as DailyDead_it-7_ increases from 0 to 1. As shown, Texas and Florida have the highest breakout potential among U.S. states (in the sense that a single death predicts that more than two will follow), whereas in some states like West Virginia and Vermont a single death would not be predicted to be followed by another.

**Fig. 5:**
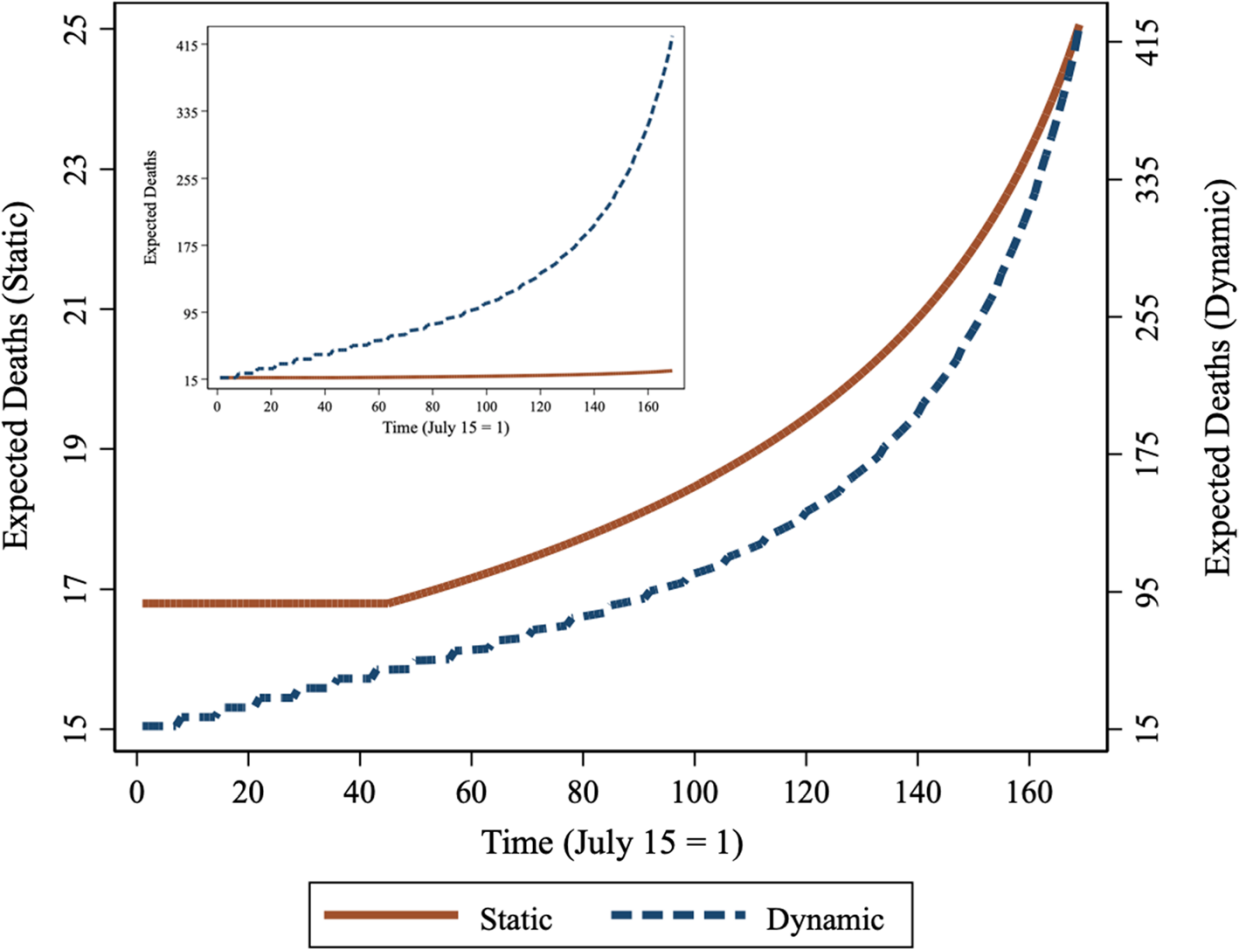
Stylized static and dynamic TRPs for Georgia derived from Eq. 1 parameter estimates. The brown solid curve [left vertical axis] is the stylized static TRP where temperature influences DailyDead_it_ through changes to MaxTemp_it-k_, with DailyDead_it-7_ held constant. The blue dashed curve [right vertical axis] is a stylized dynamic TRP under similar assumptions, except that dynamic feedback is allowed in the form of temperature influencing subsequent DailyDead_it-7_

DailyDead_it_ predictions for Georgia from both static and dynamic variants of our base model are simulated in Fig. 5. We fix MaxTemp_it-7_ and MaxTemp_it-14_ at 31 °C on July 16 and hold that temperature constant for 45 days to mimic the rest of the summer period before progressively decreasing them by 0.2 °C each day until reaching 5 °C right before the last week of December. We chose Georgia for this example because of its broad swing between mid-summer and mid-winter temperatures. The temperature range we simulate makes Georgia somewhat colder than what the state typically experiences in December, but 5 °C is not uncommon later in January.

The solid brown curve in Fig. 5 provides a stylized pure temperature response [static] (left vertical axis) representation of information contained in our base model. The two MaxTemp_it-k_ lags change in tandem, with all other variables fixed at their initial values. In this static response model expected DailyDead_it_ increases through only one channel: the direct impact of lowering temperature. There are 13 CTP reported deaths on July 16, the day after our sample period; we use this as the initial value and the relevant lag from the prior week. Immediately the brown curve jumps up to 17, reflecting the expected number of new deaths produced in a week in Georgia given a fixed infection pool variable equal to 13 deaths and a constant 31 °C temperature.

The dashed blue curve in Fig. 5 shows the dynamic counterpart (right vertical axis) that allows MaxTemp_it-k_ to influence DailyDead_it_, which is then used as the lagged model input for subsequent projections. It initially tracks the static line, however, the two processes quickly diverge and cover different ranges, so we provide an insert on this figure to show the absolute scale perspective. Now temperature affects expected DailyDead_it_ through two channels: the direct effect shown in the static model, and the indirect compounding effect that occurs when instead of holding the lagged death count constant, the currently estimated death count becomes the lagged value a week later. The indirect effect of maximum temperature is the dominant mechanism, accounting for more than 90% of the increase in the expected death count as temperature falls from 31 to 5 °C. The inset in Fig. 5 plots the two curves under the same scale and conveys the magnitude of the differences between the pure static and dynamic responses.

Neither model is likely to represent reality, but together they span it. Staying on the brown static TRP curve would prove difficult to do. It represents the expected number of deaths each day as the temperature changes provided that the infection pool is held constant at its starting level. In the face of declining temperature this requires continual actions to reduce the effective contact rate in such a way as to exactly offset the increase in transmission potential. In contrast, observing the full dynamic effect requires the absence of any offsetting actions by both the government and the public (such as increased social distancing and face mask adherence as indicators of COVID-19 activity are quickly increasing). On the 31^st^ of December 2020, there were 88 CTP reported deaths. That can be viewed in three ways. Most straightforward is that it was over a 500% increase in the death count. However, it is only about 20% of the full dynamic path, and it is also over 300% higher than if the role of temperature had been recognized and more stringent prevention measures were progressively deployed to keep case counts from rising.

Relative to our simulated values, actual MaxTemp values were warmer on average, moving toward low points on January 9^th^ and January 15^th^ between 3 and 4 °C. On January 20^th^, Georgia hit its death count peak of 138, an eight-fold increase from our first week’s 17 deaths. Since the static temperature amplification factor increases from 100% at 31 °C to 160% at 5 °C, our multiplier of 8 can be decomposed into the product of a static amplification factor and a dynamic one. With the static factor at 5 °C equal to 1.6, the dynamic amplification factor estimate is 5.

This example shows how a parameterized TRP can be used to help isolate the role of other factors and how statistical versions may be derived. Here, the controllable dynamic component has a little over 3 times (5/1.6) the impact as the static TRP factor. Minimizing the dynamic amplification factor by preventing the build-up of the infection pool as temperatures cool over time should be a key policy objective.

Georgia’s base death count generation capacity is fairly large (Fig. 4). This is not true of some geographically isolated states with small populations like Vermont, where community transmission with a negligible infection pool would be unsustainable in warm enough weather. This is not true of most states though, which is consistent with Baker et al. (2020)'s finding from examining earlier emergent viruses that warming weather, by itself, will not stop their spread.

We now turn to alternative TRP specifications for Eq. (1). These specifications: (a) look at alternative temperature scaling functions and the differences between MaxTemp entering those functions linearly or in log form, (b) substitute DailyDead_it-14_ for DailyDead_it-7_, (c) consider a variant of Eq. (1) where the dependent variable and infection pool are rolling 7 day aggregates that shift forward daily, which should help to dampen out the reporting errors, while at the same time maintaining a temperature-related time structure (details in Supplemental Material).

**Fig. 6:**
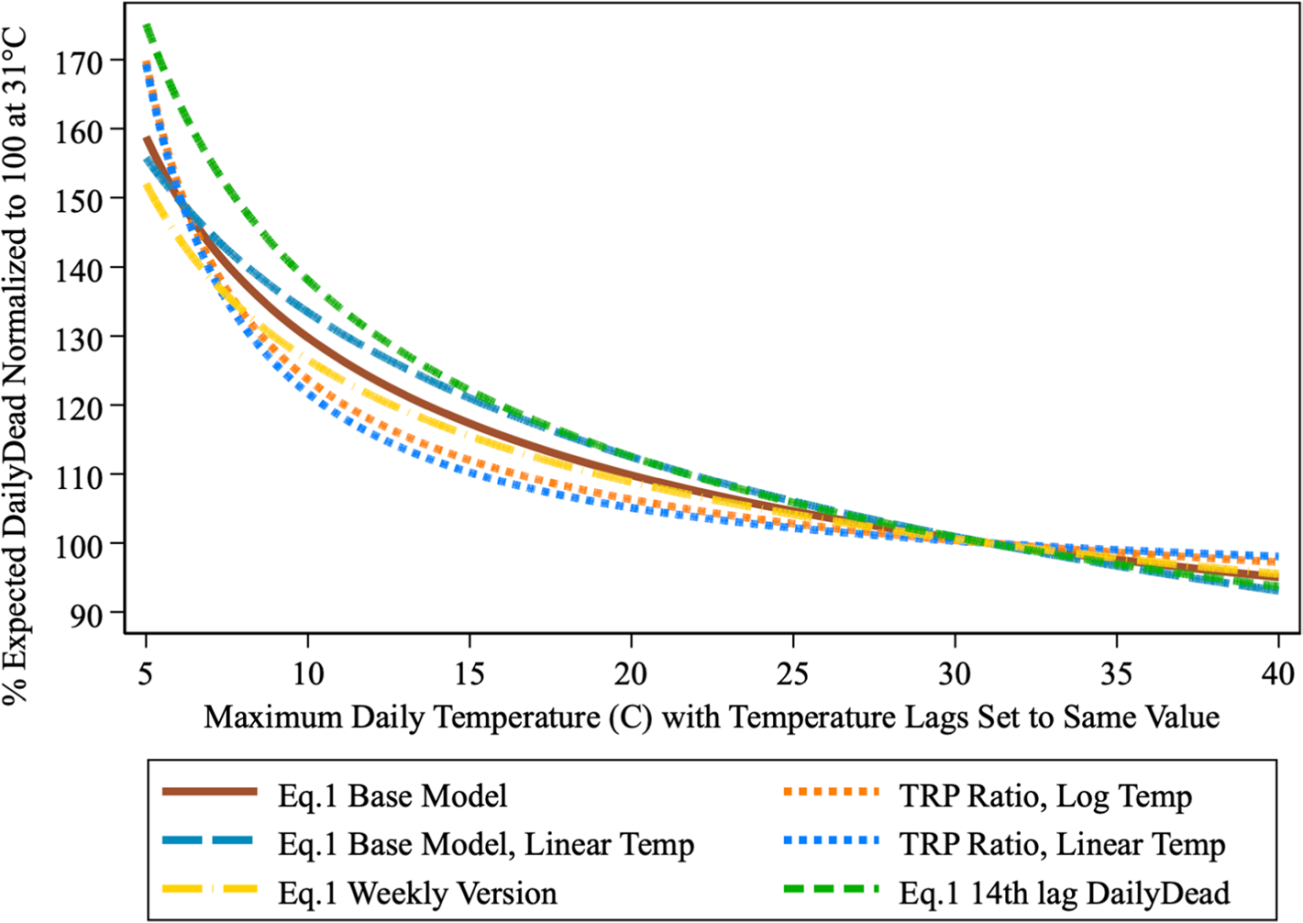
Alternative DailyDead TRPs. U.S. States: April 16-July 15

Figure 6 displays the set of TRPs from these models alongside that from our base Eq. (1) model. Model estimates for these alternative specifications are in Tables S3 and S6. Together they show that our finding of a highly sensitive TRP for DailyDead_it_ is quite robust. These alternative TRPs are remarkably similar to our base model’s and tend to bracket it. The one caveat is that some specifications suggest the TRP is flatter in the 10–20 °C range but steeper in the 5–10 °C range.

Next, we swap out the infection pool indicator. NewPositives_it-7_ is a good predictor (R^2^ = 0.95) of DailyDead_it_ (Table S6, Model 16) but not quite as good as DailyDead_it-7_. This suggests that either the positives have more measurement error than our corrected version of death counts or that DailyDead_it-7_ is a better reflection of the current risk-adjusted infection pool than NewPositives_it-7_. As would be expected, with the moving forward of the relevant infection pool, the 14^th^ temperature lag is no longer significant and is not included in the model.

Finally, estimating Eq. (1) using the original CTP death counts (Table S6, Model 26) produces considerably larger coefficients on temperature. Its TRP value at 5 °C is a little over 30% higher, with this gap declining as temperature increases. Figure S6 displays the CTP based TRP alongside our base specification. The more responsive TRP using the CTP data might surprise some due to the usual intuition that measurement error tends to attenuate parameter estimates. This is true. However, the measurement error here is not in temperature, but rather in the death counts.

#### UV, Humidity and Temperature at State Population-Weighted Centroids

Table S6 (Model 13) shows that replacing our temperature variable with the UV radiation variable identified by Carleton et al. (2021) as influencing COVID-19 activity provides a quite similar, but slightly inferior fit, relative to our base specification. Adding UV to our base specification (Table S6, Model 12) improves the overall fit, but the change is marginal, and depresses the magnitude of both sets of variables. Figure S3 shows why this is the case—the time series profiles of MaxTemp and UV are quite similar, which is not surprising given the underlying physics. We use temperature because it is more accessible, without making a causal claim for temperature versus UV. In contrast to UV, both absolute and relative humidity provide an inferior fit to models excluding MaxTemp and are insignificant in models including one of the humidity variables and MaxTemp (Table S6, Models 10 and 11).

We consider an alternative maximum temperature stimulus variable based on estimating maximum temperature at each state’s population-weighted centroid using standard inverse distance weighting (see Supplemental Material). This series is highly correlated (0.96) with our original series and hence provides similar estimates (Table S6, Model 28). The fit of this model is not quite as good though, because in the two states where the two temperature series show the most divergence (California and Florida) the point of temperature measurement has moved from where people are concentrated (on the coast) to sparser interior locations.

#### *Comparing Estimated TRP to the Relationship Implied in *the* Raw Data*

An interesting question is how our estimated TRP (Fig. 3) compares to what one might see visually in a plot of the raw data. Figure S6 provides a more sophisticated and interpretable version of such a plot where the key features of the raw data can readily be seen. It is based on a bivariate robust LOWESS (Cleveland 1979) plot of DailyDead_it_ (normalized to 100 in each state at 31 °C) on MaxTemp_it-7_. This normalization eliminates the role of population and with no temperature effect it should produce a roughly straight line. This plot suggests our TRP has isolated a substantively different and more plausible curve once one considers where observations are being generated, even though the strong role of temperature is obvious. A detailed discussion of this figure is included in the Supplemental Material.

### New Positive Case Model Results

NewPositives_it_ are modeled similarly to Eq. (1) substituting LogNewPositives_it-7_ as the infection pool regressor. Testing information is required to interpret reported state-level positives cases. We use a set of variables: LogNewTests_it_ to control for current testing intensity, LogNewTest_it-7_ to allow for a past positivity rate interpretation, PerCapitaTests_it-7_ (total tests administered per thousand lagged by one week) to help control for prior testing intensity, and an indicator variable for systematically lower reporting on Monday. We chose LogMaxTemp_it-7_ for consistency with the death count model. For the other temperature lag, the 2^nd^ lag fits best.

**Fig. 7:**
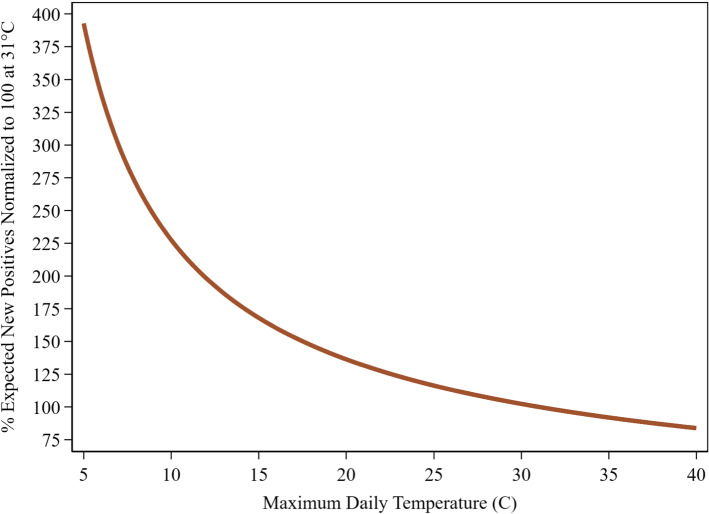
COVID-19: new positives temperature response profile. U.S. States: April 16-July 15. Temperatures for 2nd and 7th lags set equal

This model’s (Table S2) R^2^ is 0.95. All regressors are significant at *p* < 0.001, except for some of the test related variables: LogNewTest_it-7_ (*p* = 0.0584), PerCapitaTests_it-7_ (*p* = 0.003) and Monday (*p* = 0.039). Estimated parameters for the lagged temperature variables are considerably larger than their DailyDead_it_ counterparts. The testing variables tell an interesting story. At the margin, a 1% increase in the number of tests administered increases the number of positive cases by 0.39%. Essentially, more testing finds more positives, but at a decreasing rate. Testing has longer term benefits; a 1% increase in the number of tests administered a week ago decreases today’s positives by 0.18%, while a 1% increase in the cumulative number of tests administered per capita reduces today’s positives by 0.01%. Reporting of positive cases on Monday is about 8% lower than on other days. Figure 7 displays the implied TRP for NewPositives_it_. Figure S2 shows the contour plot which allows the two temperature lags to vary independently.

While we made substantial corrections to the positive case and related testing data, this data is still substantially noisier than our corrected death count series suggesting more caution in its direct use. Because positive cases should be more temperature sensitive than death counts, our measured death count TRP can serve as a reliable lower bound on the now more relevant positive case TRP, with high vaccine penetration among the older population and newer treatments like monoclonal antibodies altering the previous link between death counts and the infection pool.

## Discussion and Concluding Remarks

We show COVID-19 death counts are strongly influenced by changes in maximum daily temperature (Fig. 3). This relationship is considerably more pronounced for new positive cases (Fig. 7). Cooler temperatures can cause substantial increases in the number of deaths and positive cases over time. Dynamic feedback between a rising infection pool and cooling temperatures (Fig. 5) suggests that delays in responding to signs of increased virus activity can result in a rapid escalation of positive cases and death counts in cooling weather, while warming weather tends to have the opposite effect.

Our results suggest warming temperatures during the spring and summer of 2020 actively aided efforts to reduce COVID-19’s spread. This likely contributed to a false sense of the efficacy of prevention efforts and the lack of need for further measures. Cooling temperatures during the fall of 2020 provide an opportunity to look at whether our positive case TRP helps explain how the pandemic unfolded across states in the five months right after our sample period between mid-July and mid-December. This time frame was chosen to avoid confusing temperature amplification with the more infectious Alpha (B.1.1.7) variant (Davies 2021), which starts to become an important factor in early 2021.

To look at this issue, note that a SEIR model’s β_it_ can be expressed as the product of mean β_t_ across states, a TRP_it_, and a third factor that incorporates a demeaned version of the other factors influencing β_it_. We look at this decomposition, defining it now in terms of the change between two time periods by taking logs so the mean growth rate and temperature amplification are additively separable, with the third factor becoming the residual. We define the growth rate using the ratio of cumulative positive cases (CPCRatio), with the denominator being this statistic (from CTP) on July 16, the day after our sample period, and the numerator being that statistic on December 17, the matching weekday five months later. Measures of CPCRatio’s type are often referred to as incident rate growth ratios. We take the log of this quantity (LogCPCRatio) and regress it on the log of the matching ratio of our estimated TRP values using realized temperatures (LogTRPRatio). Note, that within a state, factors that stay constant between our two time periods, such as per capita normalizations and the average fraction of positives missed in testing, drop out of the ratio. The CPCRatio, itself, is proportionate to the average percent change in β_it_ between the two dates.

**Fig. 8:**
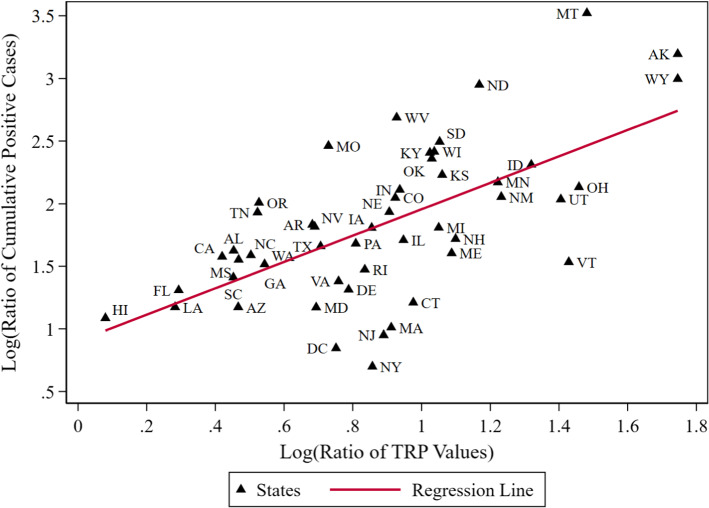
Log(growth rate in cumulative positive cases) vs. Log(ratio of temperature response profile values) between mid-July and mid-December 2020

Details on the construction of the data used in Fig. 8 are provided in the Supplemental Material. The primary concern involved the treatment of December temperatures outside the 5 °C lower bound on the range we study. We use the actual temperatures observed in the TRP value calculation down to 2 °C. For two states with negative (and hence undefined log) temperature lags, we conservatively use a TRP ratio a third higher than the next largest state due to the appearance of steep curvature in this range. This issue points to the usefulness of research that would extend the range of our TRP estimate into temperatures below 5 °C.

This simple OLS regression model has a constant of 0.09012 (6.39) with a parameter estimate on LogTRPRatio of 1.0547 (6.72), where t-statistics based on robust standard errors are displayed in the parentheses. If the standard multiplicative representation for β_it_ is correct, then the slope parameter should be 1. That cannot be rejected here. The R^2^ measure (0.40) indicates this single predictor explains just over 40% of the variability across states in the log growth rate of cumulative positive cases between mid-July and mid-December. Interpretation of this result is straightforward: a 1% increase in the TRPRatio predicts a 1% increase in the CPCRatio.

Exponentiating the constant term leads to an estimate that positive cases grew on average by 246% between mid-June and mid-December across the states, before accounting for the temperature amplification effect. The parameter on the LogTRPRatio provides a straightforward out-of-sample test of the null hypothesis (rejected at the *p* < 0.001 level) that our LogTRPRatio variable does not help predict the growth rate in a state’s cumulative positive cases, after our sample period, between mid-July and mid-December of 2020. A different way of viewing the informational content of our TRP measure is to use a simple alternative measure of the temperature change: the log of the difference between the first relevant MaxTemp lags in July and December. This parameter estimate, 0.3227 (2.69), is statistically significant, but this model’s R^2^ is only 0.06.

Figure 8 depicts the results of our regression model. As the LogTRPRatio increases from just above 1 in Hawaii to above 5 in the coldest state, the predicted conditional median growth rate in cumulative positives increases from 246 to 1570%. This increase by more than a factor of six illustrates the magnitude of the influence of temperature on COVID-19 transmission. While future weather values cannot be known in advance, for planning purposes historical weather data in a location, e.g., the 30-year average maximum temperature on each day (e.g., climate normals; National Centers for Environmental Information 2021) can be used in their place.

A less obvious feature of Fig. 8 is that the vertical dispersion of the LogCPCRatio for states near the same TRPRatio is an implication of the exercise depicted in Fig. 5. A state can keep its positive case count in check in the face of increasing temperature amplification or, to a lesser or greater degree, let this amplification escalate dynamically by allowing the size of the infection pool to increase over time. A variety of factors may help explain this vertical dispersion.

We can look, in a limited way at the role played by one of these factors—politics at the state governor level—that has received considerably more attention than temperature and which some readers might think potentially confounded with temperature. To do this, we group the Democratic governors and the four Republican governors (MA, MD, OH, VT) publicly opposing President Trump on COVID-19 policy together [NoTrumpCovidPolicy = 1] versus the Republican governors who did not publicly break with Trump on this issue. The new regression model is 1.1181 (7.39) + 1.0655*LogTRPRatio (6.45) − 0.4122* NoTrumpCovidPolicy (− 3.55). The R^2^ measure is now 0.52 suggesting that these two factors together explain just over half the variance in LogCPCRatio. The semi-elasticity associated with this indicator variable indicates the set of governors actively opposing Trump’s COVID-19 policies experienced a 34% lower growth rate in cumulative positive cases. The negligible change in the LogTRPRatio parameter estimate indicates a political factor, defined in this way, is almost orthogonal to LogTRPRatio. Adding the percent supporting Trump for president in 2020 to this model, which might proxy for a multitude of social factors influencing transmission, results in an R^2^ of 0.62. However, the parameter estimate on LogTRPRatio, 0.9676 (6.41), is largely unchanged and still statistically indistinguishable from 1. While this suggests our TRP risk factor does not incorporate political considerations, the converse is not true. Rising positive case counts coupled with rising TRP values portends danger ahead. Whether such a warning sign is heeded is a political decision.

The future U.S. path of the pandemic across the states is unlikely to repeat the pattern depicted in Fig. 8 and the type of model we used to successfully predict the virus’ behavior during its (late) early U.S. period is no longer applicable. Factors like vaccination rates, the patterns of prior infection, differential relaxation and strengthening of measures intended to influence transmission effectiveness like facemask mandates, and the nature of mixing/stratification processes between identifiable socio-demographic groups with high and low vaccination rates are now likely to be key factors, alongside the increasingly numerous COVID variants. This is not to say that the role of temperature is less important, but rather that our parameterized TRP function needs to be embedded in a SEIR model that allows for a time-varying transmission rate and accounts for this larger set of factors now driving the pandemic’s path across space and time.

This can be done by parameterizing the basic SEIR model’s constant transmission rate parameter β_0_ [= R_0_/γ_0_] as a function of the relevant time-varying factors and by taking account of the changing size and composition of the Susceptible compartment via immunity conferred by vaccination or prior infection. Five main factors are known to make the β_0_ transmission rate time-varying: varying contact rates (e.g., changes in mobility), behavioral changes (e.g., facemasks), responsiveness to temperature, waning immunity over time by those previously infected and/or vaccinated, and mutations whereby new variants of the virus become more (or less) contagious. Like other parameters assumed to be known in a SEIR model, our COVID-19 TRPs can be directly used as a parameterized function or as an informative prior in a Bayesian context.

A straightforward implication of our TRPs is that the level of vaccination needed for herd immunity falls as temperatures rise and rises as temperatures fall. This can be seen by considering the 75% vaccination level often put forward as providing herd immunity (McNeil 2020). This number follows directly from assuming R_0_ equals 4 (CDC 2021; Planning Scenarios 3 and 4, and Gallager 2021; Alpha variant), where herd immunity in a standard SEIR model is represented by 1 – (1/R_0_). Our death count TRP at 5 °C conservatively moves the required herd immunity level for Alpha (assuming R_0_ = 4 was for 31 °C) into the mid 80% range, while our more relevant positive case TRP moves this level to the low 90% range.

Preliminary UK and U.S. estimates put the more contagious Delta variant’s R_0_ in a 5 to 9.5 range – around that of chickenpox (Gallager 2021; Washington Post 2021). Assuming the lower bound, moves Delta’s required level of herd immunity from Alpha’s 75% to 80%. Conservatively assuming that Delta's R_0_ = 5 at 31 °C, our death count TRP moves that required level of herd immunity into the high 80% range at 5 °C, while our positive case TRP puts that level at 95%.

**Fig. 9:**
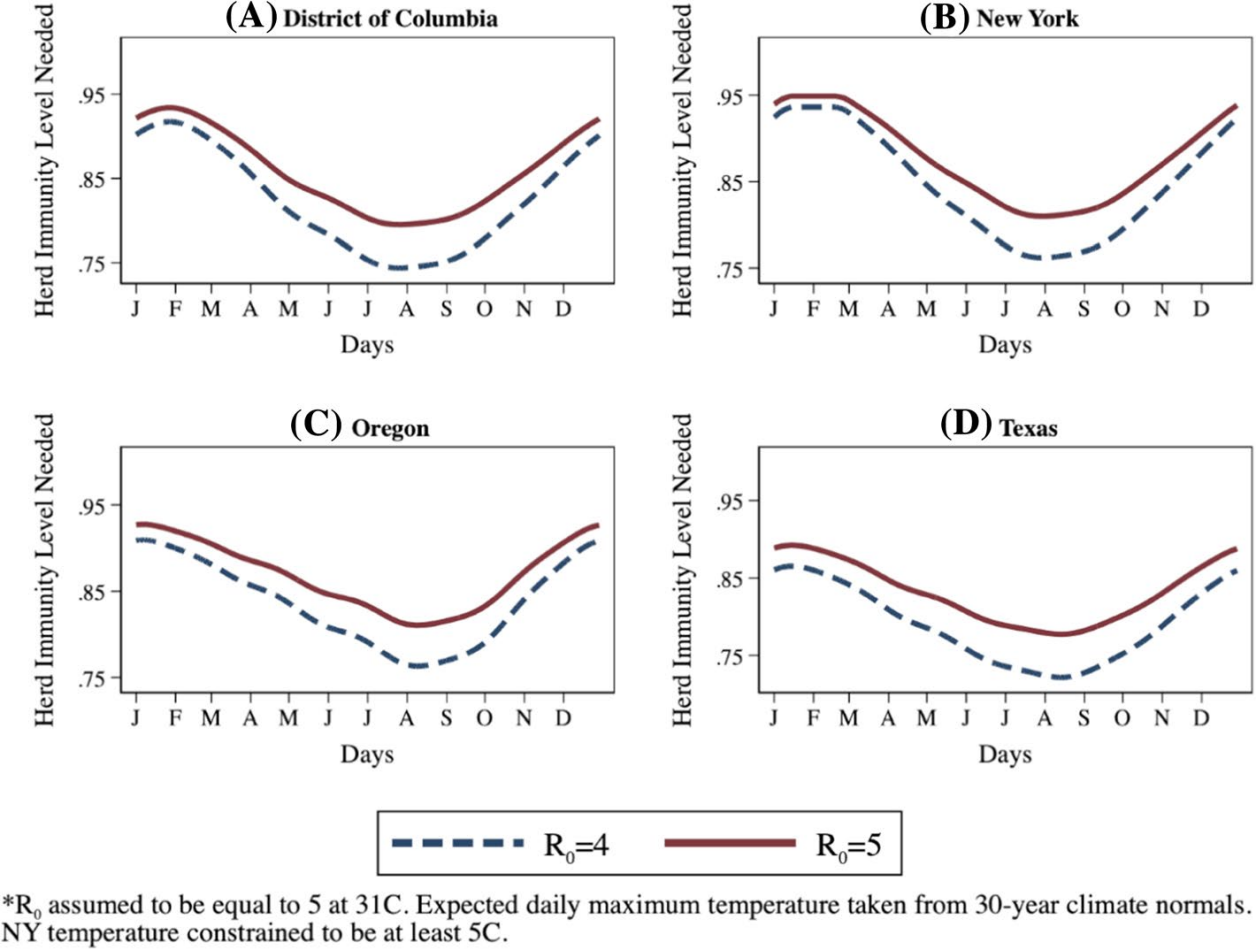
Herd Immunity Level Required for Alpha (R_0_ = 4) and Delta (R_0_ = 5) variants of COVID-19 at 31 °C by Day of Year for District of Columbia, New York, Oregon and Texas

Figure 9 plots required herd immunity levels using the positive case TRP for the District of Columbia and three states, New York, Oregon, and Texas, which span a range of climate conditions. Details on the construction of a time-varying R_0_ series, including code snippets in Python, R and Stata, are in the Supplemental Material. Both the switch from Alpha to Delta and cooling temperatures increase required immunity percentages to reach herd immunity. The normalization at 31 °C helps illustrate that in the warmest months, it is the substitution of Delta for Alpha driving the higher infection risk and herd immunity level. As one moves away from the summer months, temperature becomes the dominant factor in driving higher herd immunity levels. Together they imply tens of millions more Americans need to be vaccinated to stop the spread of the Delta variant this winter. The required increase will vary state-by-state based on current vaccination rates and the temperature-driven amplification risk they will face. For instance, from Fig. 9, Texas faces a much lower risk from temperature amplification than New York, but Texas has counterbalanced that natural advantage by vaccinating a much lower fraction of its population.

There are two more general lessons from our work. Firstly, the role played by our data correction effort cannot be over-emphasized. We transformed erratic death counts reported daily across states into orderly processes that are nearly indistinguishable from theory-driven textbook examples. No amount of modeling prowess can compensate for this problem, as demonstrated by the loss of trust by many policymakers as model predictions failed at critical junctures. The costs associated with the public health infrastructure investment in the U.S. (and other countries) required to fix the COVID-19 data reporting problems are trivial relative to the potential gains from being able to better foresee this pandemic’s path and may help contain the next pandemic before it takes a similar toll on lives and the economy.

**Fig. 10:**
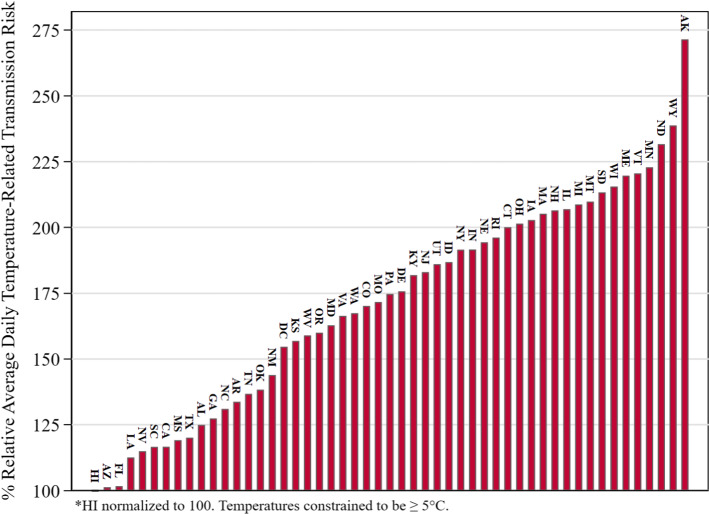
Average daily temperature-driven COVID-19 positive case transmission risk potential by state relative to Hawaii

Second, the approach put forward here may be of use in other novel situations involving large scale societal shocks. Our work is yet another demonstration that an area’s environmental characteristics can have a profound influence on public health. In this situation, we show that warmer weather conditions were an asset to mitigating the infectiousness of the novel SARS-CoV-2 virus. Figure 10 displays the relative value of this asset at the state-level.

This relative value can be obtained by recognizing that since the daily TRP value moves the temperature-driven, time-varying R_0_ up and down, the annual sum of a state’s daily TRP values is the summary measure of its annual temperature-related transmission potential relative to 31 °C. To make this measure more readily interpretable, we normalize each state’s average daily positive case TRP relative to HI, the state with the smallest average daily TRP value, and put it in percentage terms so HI equals 100. AZ and FL have only marginally higher average daily TRP values than HI. Figure 10 shows that over 2/3 of the states face temperature-driven COVID-19 transmission risk over 50% higher than HI and over 1/3 face more than a 100% higher risk from this factor. For states with very cold weather, these estimates are conservative since we have constrained the lower temperature bound when constructing the daily TRP estimate using the climate normals to 5 °C. Other statistics of the daily state-level TRP series are also relevant to managing temperature amplification risk since higher variability makes it more difficult to gauge how well prevention measures are working and the range of the temperature swing influences the level of risk from dynamic temperature amplification. The Supplemental Material provides a table (S5) of the daily TRP summary statistics for each state and construction details. The actual daily TRP series for each state based on the 30-year maximum temperature climate normals are provided in an accompanying dataset.

Unfortunately, the Trump Administration and many U.S. states failed to use the assist summer temperatures availed to help bring positive case counts down before cooler temperatures amplified transmission potential, something many experts at the time predicted (qualitatively) likely to happen, resulting in thousands of avoidable deaths. With the better data we were able to assemble, the relationship between COVID-19 and temperature is now precisely quantified.

## Supplementary Information

Below is the link to the electronic supplementary material.

Supplementary file1 (DOCX 1377 KB)
