# Supplementary material for: COVID-19’s U.S. Temperature Response Profile
Source: Environ Resour Econ (Dordr). 2021 Sep 20;80(4):675–704. doi: 10.1007/s10640-021-00603-8 (PMC8452123; doi:10.1007/s10640-021-00603-8)
Supplement: Supplementary file 1 — Supplementary file1 (DOCX 1377 KB) [file 10640_2021_603_MOESM1_ESM.docx]

Supplemental Material For

COVID-19’s U.S. Temperature Response Profile

Includes:

**Additional Discussion**

A. Data Preparation

B. Influence of Data Correction Effort

C. Construction of Temperature, Humidity and Ultraviolet Radiation Data

D. Alternative Specifications for Base Death Count Model

E. Additional DailyDead_it_ Specifications: Alternative Weather Variables

F. Use Positives as Infection Pool Indicator for Mortality

G. Role of Cumulative Death Counts

H. Univariate DailyDead_it_ and MaxTemp_it-7_ Relationship

I. Figure 8: Data Construction, Further Analysis and Implications

J. Constructing Temperature Response Profiles (TRPs)

K. Usage of our estimated TRP Functions

L. Construction of Daily TRP Series for Each State

M. Data and Code Availability

**Figures S1-S6**

**Tables S1-S5**

**Structure of Table S6**

**Table S6**

*A. Data Preparation*

The data set used in this paper starts with the COVID-19 statistics reported by individual states as aggregated daily by The COVID Tracking Project (covidtracking.com). In preliminary work, we (and other modeling groups) found there was no plausible epidemiological model that would produce the drastically varying levels of case counts in the state-level COVID-19 statistics reported daily. As a result, there is a major fork in the path for any modeling effort. It is possible build a predictive model for the reported daily death counts, where success is judged by minimizing the forecast error around observable quantities that policymakers see. The alternative is building a model where the focus is on trying to understand the behavior of a novel pathogen. When the reported dependent variable systematically diverges from what is generated by the underlying process, these two approaches also fundamentally diverge. For the first path, success, to some degree, comes in uncovering the administrative procedures influencing the divergence. The second path led us to undertake a major effort to rectify and repair reported COVID-19 statistics, with a focus on recovering the temporal structure needed to provide reliable TRP estimates.

The most important correction was replacing originally reported death counts with death counts by date of death as reflected on death certificates. Figure 1 illustrate the nature of the differences between the original CTPDailyDead_it_ and DailyDead_it_ for Florida and Georgia. The originally reported data results in substantially larger confidence intervals in predictive models. Additionally, it lags the revised death counts. Therefore, it substantially reduces the ability to detect and respond to changes in COVID-19 activity. We were able to make this correction for 33 states (comprising 77% of the U.S. population) where deaths by death certificate information could be located. The states where we have been unable to obtain deaths by death certificate dates tend to be smaller and generally have had relatively fewer COVID-19 deaths. Typically, these states did not face large COVID peaks, which appear to be associated with increased testing delays. However, we have still been unable to obtain this data from two large states – New York and Illinois – and of the 15 observations (out of 4,567) with residuals from Eq. 1 whose absolute value is 50 or more, seven are from these two states, with another four being from Florida.

The methods for collecting data backed by death certificates varied based on how the individual states chose to present them. Some states publish their counts based on death certificates in a downloadable format on their official COVID-19 website. Others publish them inside of longer reports or source code in ways that can be hard to find. In still other instances, the information is presented in tables or on graphs where it was viewable by hovering cursors over each bar in a bar chart. These cases require manual extraction into spreadsheets. Because there are no official rules for how to publish this information, even the charts vary in structure and presentation. Commonality across states is largely dependent on the specific software vendor they are using for their public-facing COVID-19 dashboards. Because of the diverse ways that death count data can be referred to, we found it important to verify via explicit labeling or official statements that the data were obtaining was COVID-19-related deaths by death certificate date. The CDC reports this data with a substantial lag (https://www.cdc.gov/nchs/nvss/vsrr/ COVID19/ index.htm), but thus far has only at the weekly level, making it unsuitable for our purposes.

For states where deaths by death certificate date were not available, we first sought to determine if an individual state, either on their COVID-19 reporting “dashboard” or in a downloadable file, had deaths by reported date. These datasets often contain substantial corrections to information that a state had originally reported for a specific day. Typical corrections included counts reported after Covidtracking.com’s daily reporting deadline, more accurate end-of-day tallies (e.g., late reporting counties/hospitals), the correction of testing dumps that resulted in the appearance of spikes on certain days, the removal of duplicate death certificates, and the resolution of probable cases. When these differed from the COVID-19 death counts originally reported (covidtracking.com has a set of “snapshots” of the originally reported information) we used the updated state data. In most smaller states we believe, but have been unable to fully verify, that these corrected datasets are “close” to deaths by death certificate date.

Two of these types of corrections turn out to be particularly important. First, when a state misses a reporting deadline no COVID-19 events (new deaths positives, or tests) are usually recorded on that date. This causes the next day to contain the events that happened during the previous day. Second, “probable” cases have often been treated differently across states and time. State-level resolution of this issue removes an extraneous source of variation. As with the data on death certificate dates, these corrections by states of their originally reported COVID-19 statistics were obtained through a variety of sources, ranging from reading counts off interactive bar charts to downloadable *.csv files.

Next, we corrected two obvious problems with the data. First, we corrected occasional upward large spikes accompanied by auxiliary information (e.g., a news/twitter release by the state’s department of health) noting that this spike was due to an accumulation of deaths over an extended period, typically from one or more congregate living facilities (e.g., nursing homes and prisons). For these, the correction involved increasing the initially reported death counts proportionately over the relevant period. There are also downward corrections that occur, for instance, when a state that initially reports probable COVID-19-related deaths, then reduces the number of deaths reported at a future date (by a specified number) to reflect the number of probable deaths in a batch that were reclassified from being COVID-19 to non-COVID-19 related because their test came back negative or to remove duplicate deaths that were originally report by the county where the individual lived and a different county where the individual died. The problem in these instances is that the state while the state reports the number of deaths rolled back, it does not report the dates on which those rolled back deaths were first reported. We use the same proportionate reallocation rule, with the caveat that corrections are not made to days where this would result in negative death counts. Second, we corrected reports of zero deaths on days surrounded by death counts on adjacent days that were sufficiently large that the actual occurrence of zero deaths was highly unlikely. Such days are generally also characterized by a failure to report some other COVID-19 statistics (like new positive cases) and by an abnormally high death count on the following day (and sometimes two if it is a weekend). This suggests that the appropriate correction is to average counts across the two and, in some instances, three days. As a concrete example, a sequence of (7, 5, 0, 12) on Friday, Saturday, Sunday, and Monday is corrected to be (7, 5, 6, 6).

We also corrected data in situations where a state had “corrected” an earlier report, but where an entire rectified data series from the state was not available. A typical example is a state that initially reported all deaths except for those in the state’s largest county, with the corrected version containing the death count for the whole state reported in in a press/twitter release. Similarly, the data fails a logical consistency check when the difference between the reported cumulative death counts on two consecutive days generate a negative daily death count. This typically occurs in states with small populations and few COVID-19 deaths, who, without comment, reduce their cumulative death count by one or two. Here we “rollback” that correction to the closest date that no longer produces a negative daily death count.

Temporal misalignment of NewPositives_it_ has received more attention than DailyDead_it_ because of the large time gap often found between the time of administration of a diagnostic test and when the result is returned. Indeed, it is this gap, coupled with different state testing regimes, which led most modeling groups to concentrate on predicting DailyDead_it_. There is no reason by test results cannot be reported by lab analysis and administration dates, where the latter file would be updated daily. This coupled, with daily reporting of the total tests administered on the prior day, would allow much better monitoring of overall testing situation.

We have made similar corrections to daily positive case counts and new daily test counts as we did with the death count data using corrected state reports and adjustment of non-reporting days. We also had to deal prorated rollbacks of initially reported antibody tests in some states. The major difference between death counts and positive cases, though, is that very few states have made positive case counts by day of test administration (rather than the day the test result was reported) available. This means that few states publish datasets that can readily subsume the positive case count data from the COVID Tracking project, which is likely due to different information reporting standards. Specifically, a state eventually knows and eventually must report death certificate recorded data of COVID-19-related deaths, but this information is subject to reporting delays due to strategic political reasons, as well as waiting for confirmation of test results or autopsies. Because the date of death is on the death certificate, obtaining official death counts for all states is eventually feasible. The lab doing the test knows the date of test administration, but this information is often not shared with the state. States could require reporting of this type of information, but whether such information about prior could ever be captured now. Total test counts are even messier due to the common practice – particularly in the earlier part of our sample period – of reporting all newly returned positive test results daily but reporting negative results inconsistently or in batches. Some negative test results (often by the state’s lab) were reported daily along with the positives, while other negatives (often those by private labs) were reported once a week. We performed rollbacks of antibody tests. For a period of time after antibody tests became available, some states mixed them together with diagnostic tests. Make a correction for this practice is complicated by, in contrast to death counts from particular facilities, the state not providing the time period over which this mixing of test types occurred. When an approximate start date could be reasonably inferred, usually via a sustained jump in testing, without the state making claims (e.g., via twitter or press release) about testing capacity being substantially expanded in some way, we have averaged and prorated the testing data needed to be rolled back.

The temporal misalignment of the NewPositives_it-k_ is somewhat less important for deaths than it might first appear because there is a shorter window for positive-to-positive transmission than the positive to death transition and test results for hospital patients, the persistent high positivity pool, are typically returned quickly. Even very noisy information on the pattern of test deployment can help to serve as controls for temporal variation in state-level testing.

*B. Influence of Data Correction Effort*

The influence of our data correction efforts can be quickly gleaned from Table S4, which describes four simple autoregressive models with a constant term and 7^th^ death count lag. The first uses the original “Reported” dataset (CovidTracking.com) as the sources of the dependent variable, CTPDailyDead_it_, and its associated lags. The second uses our corrected version as the “Revised” for the dependent variable and the originally Reported for the lagged regressor. The third uses CTPDailyDead_it_ as the dependent variable and the Revised for the lagged regressor. The fourth uses Revised for both. The parameter estimates for lagged deaths are similar in versions using the same lagged variable and substantively larger in the two versions using lagged revised counts. The R^2^ starts at 0.69 for the Reported/Reported model, stays roughly the same (.70) if Revised are predicted from CTPDailyDead_it-7_, and increases somewhat further to 0.75 for the Reported/ Revised combination. However, the Revised/Revised combination, has an R^2^ of 0.93, which clearly illustrates that the explanatory power (R^2^ of .97) of our base Eq. (1) model in Table A1 comes mainly from our data repair and rectification effort. Note that in moving from this simple model using the Revised data to that of Eq. (1), the root mean square prediction error falls by over 30%. Note that all of the models in Table S4 set the massive NJ (June 25) reported death count outlier of 1877 (Revised death count is 16) to missing, since it is so large that many modeling groups have either dropped it or prorate it over earlier time periods. The R^2^ of this Reported/Reported model using these observations falls to 0.42.

*C. Construction of Temperature, Humidity and Ultraviolet Radiation Data*

Weather data for our main analysis is drawn from the National Centers for Environmental Information (NCEI) Integrated Surface Database (ISD), which reports hourly temperature and humidity data for most airports in the world. For each state, weather variables are taken from the airport with the highest volume of commercial traffic, where the volume information is found in the Federal Aviation Administration’s 2018 Commercial Service Enplanements report. Our key variable of interest is daily maximum temperature (MaxTemp). This variable, used as the 7^th^ and 14^th^ lag, has considerable variation over our sample period with an average means of 24.7°C and 23.7°C, respectively, standard deviations of 7.4°C, as well as a min of 5°C and a max of 43.9°C. An alternative maximum temperature series constructed at each state’s population-weighted centroid using standard inverse-distance weighting is described in the Supplemental Materials.

We also look at measures of humidity and ultraviolet radiation. Hourly relative humidity is calculated as a function of hourly observed temperature and dewpoint temperature. Under the assumption of ideal gas behavior, we calculate hourly absolute humidity as well (details can be found at https://www.hatchability.com/Vaisala.pdf). We then pick the highest readings within each 24-hour period as daily MaxTemp, MaxRelativeHumidity, and MaxAbsoluteHumidity. Minimum daily temperature is obtained by picking the lowest reading and the mean is obtained by averaging the hourly readings. Our measure of ultraviolet radiation is the UV index, which provides a forecast of the expected risk of overexposure to UV radiation from the sun. UV index data at our representative airports is obtained from OpenWeather Ltd., which publishes daily UV index forecasts calculated by the National Weather Service.

*D.* *Alternative Specifications for Base Death Count Model*

Table S3 compares our base model to a set of alternative specifications. These alternative specifications were chosen to look at the sensitivity of the implied TRP because they all have reasonably similar fits relative to our base model. This allows us to observe how robust the TRP is to various modeling decisions that we made. The first specification replaces the LogMaxTemp_it-k_ with their linear counterparts. On the surface, this seems like a decision regarding which of two different scales fits better, but because the models have estimated parameters in the scale function, it is possible for the two different specifications to provide reasonably similar TRPs. The second uses a popular ratio scaling function LogMaxTemp_it-k_/(LogMaxTemp_it-k_ + α), where α is an estimated parameter. The third replaces LogMaxTemp_it-k_ with MaxTemp_it-k_ in this ratio scaling function. A potential issue with Eq. 1, is the possibility that LogMaxTemp_it-14_ also has some influences our main infection pool indicator LogDailyDead_it-7_. Our next specification replaces our infection pool indicator with an alternative, LogDailyDead_it-14_, so both temperature variables are now clearly exogenous from the temporal perspective of the infection pool indicator.

The last is a weekly variant (Eq. S1) of the base model, where the dependent variable is the sum of the daily death count of the next seven days, with corresponding backward shifts of the lagged variables. This model averages out much of the daily variation and many types of administrative reporting practices. In forecasting the pandemic’s progression, it has become common to use data aggregated to a weekly level in an effort to average out many of the administratively-induced reporting issues that our extensive reconstruction and repair of the daily death count data sought to alleviate. A variant of Eq. (1) can be estimated that uses death count data aggregated into seven-day periods, WeeklyDead_it_ = Σ_t_ DailyDead_it_, where the summation is over t=1 to t=7. This makes WeeklyDead_it-7_ the sum of the 7th through 13th lags of DailyDead_it_. Importantly, this aggregation does not reduce the number of observations because on each day, the weekly aggregation at t=1 adds a new observation DailyDead_i1_ and drops DailyDead_i8_. Lags of the WeeklyDead_it_ variable can then be used in the standard way. One implication of this specification is that the temperature variables need to shift backward. For conceptual consistency, we use LogMaxTemp_it-14_ in place of LogMaxTemp_it-7_. Empirically, the model fits best when the second temperature lag is LogMaxTemp_t-18_. The model fit is reasonably similar using the 13th lag and 19th lags. Beyond the 19th lag, the temperature variable becomes insignificant, suggesting that older temperature information is not useful. The model we report is thus:

WeeklyDead_it_ = EXP(Σ_i_ StateIndicator_i_ + α_1_LogDays_t_ + α_2_LogDays_t_^2^ + βLogWeeklyDead_it-7_)

*(1/(1 + EXP(LogMaxTemp_t-14_)^ϓ1^))*(1/(1 + EXP(LogMaxTemp_t-18_)^ϓ2^)) + ε_it_. (S1)

The overall impression from Table S3 is the general stability of the common parameters for time and the infection pool. Some of the specifications offer insights into the latter variable. It is not influenced very much by whether the logistic or ratio scaling function was used, nor whether LogMaxTemp_it_ or MaxTemp_it_ was the stimulus variable. Using LogDailyDead_it-14_ in place of LogDailyDead_it-7_ results in model that explains less of the variance (0.94) and is somewhat less responsive to past deaths counts (.80 vs .86). It’s estimated TRP is similar to that of the base model (Figure 6), although it does rise bit more sharply between 5°C and 10°C. This suggests that if there is an endogeneity effect, it is not large and that our base TRP is conservative. In the rolling weekly model (Table S3, Model 7), the coefficient on the infection pool indicator shifts from the .86 in our base model to .92 in the rolling weekly version of our model. The R^2^ for the weekly model increases to 0.99 (from the base model’s 0.97).

*E. Additional DailyDead_it_ Specifications: Alternative Weather Variables*

The other weather variables which have received considerable attention are absolute humidity, relative humidity, and ultraviolet (UV) radiation. Details of construction are provided below in the Data Preparation Section of the Appendix. The limitation with all of these weather variables is that their correlation with MaxTemp_it_ (i.e., absolute humidity: 0.58, relative humidity: -0.21, UV: 0.79).

*Humidity*

Maximum absolute humidity has a reasonable size effect (p < 0.001) in the model where it replaces the corresponding MaxTemp_it_ variables. However, the LogAbsoluteHumidity_it-k_ parameter estimates are close to zero and no longer significant when the two parallel logistic functions comprising the temperature scaling function are added (Model 10 in Table S6). Relative humidity has a marginally significant relationship with DailyDead_it_ when MaxTemp_it_ is not in the model. Its effect is close to zero in a model with MaxTemp_it_ (Model 11 in Table S6).

*UV*

The situation with UV_it_ is more complex (Table S4 &Table S6, Models 12 and 13). The MaxTemp_it_ lags are marginally better predictors in a head-to-head comparison. In the model (12) with both sets of predictors, R^2^ improves by .0003. The obvious problem is the strong correlation between the two series. The first MaxTemp_it_ lag is still highly significant while the second is significant at the .05 level. The second UV_it_ lag just misses being significant and is at the .05 level (Table S3). It is effectively impossible to disentangle the influence of LogMaxTemp_it_ and UV_it_. Their relationship is displayed in Figure S3, which plots LogMaxTemp_it_ and LogUV_it_ for two states over our sample period: Georgia (Atlanta) and New York (New York City). We cast our results in terms of MaxTemp_it_ rather than UV_it_ because it is more widely reported and understood, without making any claim our work supports a joint versus singular causal mechanism.

*MaxTemp at State Population-Weighted Centroid*

As an additional robustness check, we construct an alternative maximum temperature series at each state’s population centroid. This temperature series is derived using standard inverse distance weighting procedures. We interpolated daily temperature values at each state’s population centroid using all of the weather monitors that are located within 200km of that state’s population centroid.

This alternative temperature series has a correlation of .96 with the main airport in the state temperature series. Most individual states have higher correlations than this. Some sparsely populated western states like Montana and Wyoming that have no large city have correlations in the low .9 range. In only two states, California and Florida, do the two series have lower correlations which are both in the low .6 range. The population centroid approach moves the point of temperature measurement from the Los Angeles International Airport (LAX), on the coast, north and inland toward Bakersfield. In Florida, the centroid approach moves the point of measurement and it moves from the Miami International Airport (MIA) toward the center of the state which is east of Tampa and southwest of Orlando. Both of these new measurement locations are less representative of the coastal locations where the pandemic was seeded and spread from than Los Angeles and Miami.

Use of any single temperature series for California, a large geographically diverse state that on any given day can feature some of hottest temperatures in the U.S. (e.g., Death Valley) and some of the coldest (e.g., Sierra Mountains) with a very population concentrated in many places. During our sample period exposure to COVID-19 was concentrated coastal areas which received heavy initial seeding (largely from LAX but to a lesser degree SFO) where LAX’s moderate, low variability temperature series appears to be more appropriate than using the temperature series from Bakersfield’s airport (BFL) roughly 120 north and inland from LAX and near California’s population-weighted centroid. Temperatures at BFL are warmer in the summer and cooler in the winter than LAX, thereby exhibiting more temperature swing than LAX. We replace LAX with BFL for analyses outside our sample period as being more representative of temperature amplification faced by California. However, we note temperature swing at BFL is still quite small and may still underestimate the temperature variability influencing transmission in California.

Florida represents a different situation from California that can best be visualized by noting that it is common for a hurricane to extend across Florida from its east (Gulf) coast to its west (Atlantic) coast and over a period of a couple days move north up the coast. Average MaxTemp in April is less than 3°C, in July less than 1°C and less than 4°C in December warmer at Lakeland Linder International Airport (LAL), near Florida’s population-weighted centroid, than it is at MIA. Thus, Florida is consistently characterized by warm temperatures (relative to most other U.S. states), low variability and slightly (temporally) out-of-sync between MIA and LAL. As such, in comparison with other states, both of these Florida temperatures series behave in a similar manner.

A version of the base model using this alternative temperature variable is provided in Table S6, Model 28. The fit of this model is not as good as with the original temperature series. The two lagged population-centroid temperature variables are significant at the p < 0.001 level, and the TRP estimated from this model is reasonably similar but a bit attenuated.

At a more general level, a population weighted temperature average is not the correct concept. This is because it does not control for exposure via localized infection pools. Temperature in locations with no positive cases is irrelevant. Initially, and still to a large extent during our sample period, COVID-19 tended to have spread outward from large U.S. airports. With the exposed population tending to be concentrated near such airports, temperature’s accurate measurement at them and the fact that temperature at these airports is highly correlated with an alternative series estimated at each at state population centroids, are all arguments in favor of the temperature stimulus we use.

*F. Positives as Infection Pool Indicator for Mortality*

The specification in Eq. (1) is cast in terms of DailyDead_it_. If MaxTemp_it-k_ influences COVID transmission via the link between positive cases, then we should be able to replace DailyDead_it-7_ with NewPositives_it-7_. Estimates for this model (Table S6, Model 16) show LogNewPositives_it-7_ being significant at p < 0.001 and the R^2^ measure falling a bit. The coefficient on MaxTemp_it-14_ is insignificant, which would be expected if NewPositives_it-7_ incorporates that information, while the coefficient on LogMaxTemp_it-7_ is larger than in the base specification.

*G. Role of Cumulative Death Counts*

A different aspect of the COVID-19 death statistics that has not been incorporated into the model is lagged cumulative death count, TotalDead_it-k_. If DailyDead_it-k_ can be seen as a proxy for the infection pool influencing DailyDead_it_, then TotalDead_it-k_ (normalized by population) is proxy for the fraction of the population that is no longer at risk from COVID-19, in the sense of being either that they have been removed from the susceptible fraction of the population via death or immunity via prior infection. A key feature of a SEIR framework is that this “Removed” fraction becomes a large enough fraction of the population to start noticeably slowing successful transmission of the virus. We can perform a simple test of this point was reached during our sample period. Adding LogPCTotalDead_it-7_ effectively makes StateBase_it_ dynamic but in a different way than the quadratic time trend by letting each state evolve according to its own pattern of deaths. Table S6 (Models 14 and 15) display the results of this model with LogPCTotalDead_it-7_ entered as (a) a second order polynomial and (b) a fourth order polynomial.

Three results are worth noting. First, the increase in explanatory power is small. The most noticeable changes are in the expected places: the StateIndicators and a substantial reduction in importance of the overall quadratic time trend. Second, the LogMaxTemp_it-k_ parameter estimates are similar to Eq. (1) which suggests that our TRP is robust to a substantial dynamic reparameterization of the model. Third, in the quadratic specification, DailyDead_it_ is declining with as LogPCTotalDead_it-7_ increases but at a declining rate. This is not what would be expected the if this proxy for a SEIR model’s Removed fraction had grown large enough to be a substantial force acting to suppress transmission. In the fourth order specification, all the LogPCTotalDead_it-7_ terms are insignificant (although jointly significant). Figure S4 displays, starting at .05, the two response functions for LogPCTotalDead_it-7_. Like the quadratic time trend, they suggest a sharp drop in how DailyDead_it_ is influenced by DailyDead_it-7_ as LogPCTotalDead_it-7_ increases from low levels with the fourth order polynomial being flatter at high levels of LogPCTotalDead_it-7_ than the quadratic. The influence of this factor is reasonably small, by the time a state hits 10 deaths per 100,000, a condition that characterizes 80% of the states at the end of our sample period. Earlier, we noted one interpretation of our quadratic time trend was that medical care (and hence death rates) had improved sharply at first and then declined. This specification has a similar interpretation but suggests that some of that learning is state specific and related to its prior COVID-19 caseload. There is no indication this rate of decline is accelerating even in the hard-hit Northeastern states where deaths per 100,000 can be as high as 175 (NJ). This feature should emerge looking at the fourth order polynomial representation, if the data supports it. That is not the case though. This suggests the population fraction previously infected by mid-summer 2020 was still too small to substantially slow transmission.

*H. Univariate DailyDead_it_ and MaxTemp_it-7_ Relationship*

If we have succeeded in isolating the TRP using the set of StateIndicator_i_ and a quadratic time trend, a simple regression of DailyDead_it_ on lagged MaxTemp_it-7_ without the state fixed effects and quadratic time trend should reveal a substantially different curve than our estimated TRP displayed in Figure 3. Figure S6 displays this relationship using a robust LOWESS smoother (bandwidth 0.2) on MaxTemp_it-7_. This curve is dramatically more sensitive to temperature between 10°C and 30°C. Below 10°C the curve drops, which is expected since states with temperatures near 5°C are isolated and smaller population-wise. The curve bends up near 35°C. There are 521 observations in this range and include all 50 states plus DC. These observations, however, are disproportionately (p < 0.01) come from states with larger state fixed effects than are predicted from the demographics alone in Table 2, which helps explain why this bump up at high temperatures does not show up in the TRP from our model. We look further looked specifically at whether a version of Model 3, where in addition to its linear temperature term, a quadratic is added. A likelihood ratio test does not suggest that this additional term is needed, at any conventional level of statistical significance.

Pinning down the TRP further will require: (a) obtaining more data over a longer time horizon with more temperature variation and, in particular, the important -5°C to 5°C range, where due to our sample period data was too sparse to make reliable inference (b) obtaining death certificate data information from the few remaining large states (i.e., New York and Illinois) where it is not yet available, since states with death certificate date reporting have prediction errors that are substantially smaller (p < 0.001) than those that don’t (Table S6, Model 2), or (c) having high quality temporally aligned COVID-19 statistics at the county level, which would provide a better temperature-infection pool match and dramatically increase sample size.

*I.* *Figure 8: Data Construction, Further Analysis and Implications*

Figure 8 is based on data on positive cases taken from the COVID Tracking Project for two dates. The dates are Thursday July 16^th^, which is the day after our sample period and, Thursday December 18, its counterpart five months later. A state’s cumulative incidence rate of positive cases was calculated for each of these two days by dividing the cumulative number of reported positive cases reported by state on those two days by its population. The ratio of the December to July positive case incidence rates (x 100) provides the percent change (growth rate) in positive cases between these two dates. Note that normalization by population is not strictly needed here, because it impacts both the numerator and denominator in the same way and hence drops out. This is also true of other factors and, in particular, each state’s average fraction of positive cases not identified through testing drops out.

In constructing the dataset for Figure 8 and the regression underlying it, there are several data issues that need to be address. The first is that there are eight states with lagged December temperature values below the 5°C lower bound on the temperature range which our model was estimated on. For 6 of these states, the relevant temperature lags are between 2°C to 5°C. While clearly the conservative option is to constrain the TRP ratio estimates to that of ID, both of whose relevant lags are 5°C, like most regression models ours should be an informed approximation in neighboring regions. The appears to be the case here but note that the TRP is increasing quickly between 2°C and 3°C. AK and WY have negative temperature values, which technically makes the TRP ratio undefined due to the log-log structure. Looking at the curvature, noted above, we conservatively (a higher value would have fit the model better) set the TRP ratio a third larger than the largest of the other states. Uncertainty over how to best treat temperatures below 5°C points to the need for research to expand the range over which COVID-19’s TRP has been estimated with high precision.

A different problem exists with the TRP ratio for two other states CA and MT. CA during our sample period is reasonably defined using LAX’s temperature series because of concentration of the pandemic along the coast (from which it radiated inland) and because the temperature differentials going inland tend to be less in the spring and early summer than other parts of the year. Between July and December, 10°C would be a large difference between any two randomly chosen dates at LAX. As discussed above when considering use of an alternative single point in a state (e.g., state population centroid) for temperature measurement, there is no perfect way to represent California’s temperature given its size and population configuration during this later period of the pandemic. For Figure 3, we shift the point of measurement to Bakersfield’s Meadows Field (BFL), the nearest commercial airport to the state’s population centroid. At BFL, a swing of 20°C between July and December would be common. This is still a small amount of swing compared to most states. Using BFL moves CA from having 2^nd^ to 4^th^ smallest temperature swing among the states. MT also has the problem of a realized temperature smaller than what the state actually experiences, but for a different reason. MT had an unusual weather event that drove MaxTemp from 4°C to 17°C to -6°C in a relatively short time window. Our first lag falls (12°C) near the high point, creating the false impression that mid-December is warm in Montana. The average MT MaxTemp value over the first two weeks is close to 4°C, so we use that value for both lags. Due to the predicted increasing steepness of the TRP as it moves below 5°C, the estimation of MT mid-December TRP using 4°C lags is likely to be conservative.

The other data issue involves the cumulative positive incident rate ratio for Hawaii. At the end of our sample period, Hawaii is just starting to experience its first large wave of COVID cases, the last state to do so, after having sustained sporadic community transmission of several months. Hence, this state has a very low baseline cumulative positive case count in mid-July and its incident rate ratio based on a different context than the other states. The most straightforward thing to do is to jump forward two months to the third Thursday in September and use its cumulative incident rate. We match this with (a) increasing the mid-December cumulative positive incident rate by a factor of (5/3) to account for using only three months rather than five and (b) defining the TRP ratio over this mid-September to mid-December. There is no perfect solution here, but reasonable alternatives, including dropping Hawaii, altogether tend not to change the parameter estimates of the regression model much. We looked at three other states that might have a similar issue, AK, MT and WY, but each of these states had substantially higher cumulative counts and were experiencing two to six times more daily positives in mid-July than HI.

The finding from the regression model underlying Figure 8, that the parameter estimate for LogTRPRatio is highly significant p < 0.001 and statistically indistinguishable from 1. This result is reasonably robust to alternative treatments of the TRP ratio (including making no corrections and constraining temperatures used in the TRP estimate to be 5°C, if they fall below that bound). The main import of the changes described here is to increase the fraction of the variance explained from ~30% to ~40%. Moving one month earlier or later for HI’s positive incident ratio reduces the variance explained by 1 to 2%. The other changes we made are conservative, in the sense that giving California more temperature swing, making Montana colder than 4°C, or extending the TRP range further for Alaska and Wyoming would have resulted in a marginally higher R^2^.

Incident growth rate ratios tend to be quite asymmetric, which is the case here. We use the log of an incident rate ratio as our dependent variable. This transformation of an incident rate ratio tends lead to a variable that has a normal distribution, a property that cannot be rejected here (Shapiro-Wilk test (p=0.2877)) and lends itself to the usual elasticity interpretation in a simple log-log regression model specification with the log of the TRP ratio as the predictor variable.

*J. Constructing Temperature Response Profiles (TRPs)*

To compare the TRP’s implied by the models in Table S4, we plot the functions using two independent random uniform variables RTemp and RTemp2, defined over the range 5°C and 40°C. The logistic scaling function with two temperature variables, LogMaxTemp_it-7_ and LogMaxTemp_it-14_, and corresponding Eq. 1 estimated parameters ϓ1 and ϓ2 (Table S4), results in the following scaling function in the base model:

(1 /(1 + exp(LogRTemp^ϓ1^)))*(1 /(1 + exp(LogRTemp2^ϓ2^))). (S2)

There is a fundamental indeterminacy in such a scaling function, in that multiplying the production function part of Eq. 1 by a constant will result in an offsetting change in the scaling function which maintains the same expected value for the dependent variable. Note that each of the two multiplicative components of Eq. S2 converge to the constant value of .5 irrespective of temperature values as ϓ1 and ϓ2 become increasingly negative. Logistic functions are typically normalized to lie between 0 and 1 by changing the “1” in the numerator to “2”, but this is not needed with our normalization to 31°C, which solves the indeterminacy from the perspective of comparing curves. This is done by calculating the value of the estimated scale function at 31°C:

(1 / (1 + exp(log(31)^ϓ1^)))*(1 /(1 + exp(log(31)^ϓ2^))). (S3)

Dividing Eq. S2 by Eq. S3 produces a function which equals 1 at 31°C. Multiplying this quantity by 100 produces a function which has a natural percentage interpretation and equals 100 at 31°C. Note for prediction purposes, the original scaling function parameters need to be used.

The ratio scaling function with parameters α_1_ and α_2_ using RTemp and RTemp2 is:

(LogRTemp/(LogRTemp + α_1_)) *(LogRTemp2/(LogRTemp2 + α_2_)). (S4)

As α_1_ and α_2_ converge to zero, both multiplicative components converge to 1 irrespective of temperature values. The value of this function at 31°C can be calculated in a manner similar to that described for the logistic. Dividing Eq. 5 with the value of that function at 31°C and multiplying by 100 produces the desired TRP.

*K. Usage of our estimated TRP Function*

A temperature-referenced, and hence time varying R_0_ series based on our positive case TRP for any location can be constructed from a set of two inputs and the two temperature parameters estimated in Table S2 (for the death count TRP use the temperature parameters from Table S1). The first input needed is a base R_0_ defined at a particular temperature. Here, we assume a lower end of estimate of R_0_ = 5 for Delta variant at the mid-summer U.S. average temperature of 31°C (adjustments to our TRP for normalization to a different temperature point is straightforward). Second, a representative temperatures series, such as a specific prior year. For an expected temperature series, we recommend either the 30-year historical average known as climate normals or their 15-year counterparts, which have higher variance but better incorporate recent climate change trends. For U.S. locations, they can be found here: https://www.ncei.noaa.gov/access/us-climate-normals/. We use each state’s largest airport weather station to represent that state. For the District of Columbia, we use Reagan National Airport, adjacent to it in Virginia. Linking between locations, day of the year and temperature can be obtained by using historical temperature averages.

We provide code snippets for constructing a TRP in the two box immediately below for Python and R and more complete pseudo code for implementation in Stata in the bottom box.

*Code Snippets: R (left), Python (right), and Stata (bottom)*

V1 <- 0.5435

V2 <- 0.6873

relative_trp <- function( temp_lag7, temp_lag14 ){

return( (1.0 / (1.0 + exp(log(temp_lag7 ** V1)))) *

(1.0 / (1.0 + exp(log(temp_lag14**V2))))

)

}

NORM_CONST <- relative_trp( 31, 31 )

normalized_trp <- function( temp_lag7, temp_lag14 ){

return(relative_trp(temp_lag7,temp_lag14)/NORM_CONST)

}

import numpy as np

V1 = 0.5435

V2 = 0.6873

def relative_trp( temp_lag7, temp_lag14 ):

return (1.0 / (1.0 + np.exp(np.log(temp_lag7)**V1))) * \

(1.0 / (1.0 + np.exp(np.log(temp_lag14)**V2)))

NORM_CONST = relative_trp( 31, 31 )

def normalized_trp( temp_lag7, temp_lag14 ):

return relative_trp( temp_lag7, temp_lag14 ) / NORM_CONST

*Import [e.g., Excel] 30-year “TempMax” climate normals for location by Month and Day using as L7. And L14. lags.

*Define base R0 value at 31°C

gen R0Base=5

*TRP before normalization use Table S2

gen UnNormed_TRP=///

( (1/(1 + exp(log(L7.MaxTemp)^0.5435))) * ///

(1/(1 + exp(log( L14.MaxTemp )^.6873))))

*Calculate value of TRP at 31C

gen T31_TRP=((1 /(1 + exp(log(31)^0.5435)))) * ///

((1 /(1 + exp(log(31)^0.6873))))

*Calculate 31C normalized version of TRP

gen TRP=(UnNormed_TRP/T31_TRP)

It is important to note that use of our TRP functions with MaxTemp below 5°C is outside the range on which they were estimated. For MaxTemp values between 2°C and 5°C, use the value of the TRP at 5°C is a reasonable conservative approximation. However, our TRP is not defined for temperature values at or below freezing (0°C) due to the log-log structure of Model 1 and, given differences in droplet behavior near or below 0°C, there is no reason to expect that our analysis can be extrapolated to this range.

Figure 9 displays the time varying level of herd immunity (via temporary immunity provided by vaccination and prior infection) required for the District of Columbia, New York, Oregon and Texas using R_0_ = 4 (Alpha variant) and R_0_ = 5 (Delta variant) under the assumptions that these are conservative values for the two variants at 31°C, the Model 2 positive case temperature parameters from the logistic scaling function and the 30-year daily climate normals at their major airport’s weather station (DCA, JFK, PDX and DWF). Note that in Figure 9, Panel B (New York), the flat spot is due to substituting in the value at 5°C for the period where expected MaxTemp is lower, in New York’s case hitting 3.9°C/39°F.

In a SEIR model, where β_t_ (or R_t_) is already parameterized to capture the average temperature effects, our TRPs can be used to help provide the foundation of location-specific spatial and temporal components. The intuition here is that the magnitude of the average temperature amplification effect can be removed directly or via absorption into a statistical procedure. This leaves location-specific seasonal patterns defined in terms the magnitude of the swing in R_0_ across days unchanged. The average effect will always be positive due our 31°C normalization, but note that other normalizations of the TRP are straightforward and may be needed for particular model specifications.

An open question for empirically-oriented SEIR models is whether the TRP actually influences β_t_ in the multiplicative manner suggested by the basic SEIR model structure and, which in turn, influences the amplitude of the swings in R_0_ noted above. A simple more flexible specification parameterizes β_t_ would use TRP^λ^, where λ is an estimated model parameter (or drawn from a distribution for simulation sensitivity purposes). λ is the usual Box-Cox (power) parameter, where λ=1 represents the multiplicative special case and λ=0 the special case where the TRP’s influence increases at a slower logarithmic rate. Other more general but still parsimoniously-parameterized functions, where the multiplicative form is embedded as a special case, are also available

*L. Construction of Daily TRP Series for Each State*

The TRP series for each state is constructed with U.S. Daily Climate Normals (1991-2020) dataset obtained from NOAA. We extract daily maximum temperature at each state’s major airports (the same set of airports used in the base models, except for substituting BFL for LAX in CA). To get the unnormalized positive case TRP at each state, we use equation (S2), set LogRTemp be the 7^th^ lag of log daily maximum temperature and LogRTemp2 be the 14^th^ lag, like in the base model. Daily maximum temperature below 5°C is outside the range on which they were estimated. Therefore, we substitute daily maximum temperature whose values are below 5°C to 5°C. We also provide another series of state TRP by setting the constraint to 2°C. ϓ1 and ϓ2 are extracted from Table S2, with ϓ1=0.5435 and ϓ2=0.6873. We also calculate the value of the estimated scale function at 31°C as shown in equation (S3), by using the same set of ϓ1 and ϓ2 values. Dividing the unnormalized TRP by TRP at 31°C produces a function which equals 1 at 31°C. Multiplying this quantity by 100 produces a function which has a natural percentage interpretation and equals 100 at 31°C. This is the normalized daily TRP for each state, and its summarized statistics are reported in Table S5. A similar approach can be used to obtain estimates for the death count TRP using the temperature parameter estimates from Table 1, using either the 5°C or 2°C lower-bound temperature constraint. The daily climate normals for each state, the daily positive case TRP assuming a 5°C constraint, the same using a 2°C constraint, the daily death count TRP assuming a 5°C constraint, and the same using a 2°C constraint are provide in an Excel spreadsheet.

*M. Data and Code Availability*

The data used in this study is archived at https://github.com/dcmoyer/COVID-TRP in the form of a Stata “.dta” file. An Excel version of this file was created using StatTransfer. The Stata “do” file creating the data set contains a line-by-line set of the changes made to the original covidtracking.com data set and the providence of those changes. Three (sets) of additional Stata “do” files are available in this archive. The first “do” file contains the code (Stata 16.1) for the regression models reported in this paper. The second “do” file provides an example of how to estimate the static and dynamic temperature response profiles for an individual state, using Georgia as an example. Third data and “do” file contains Stata code for creating the basic versions (fine labeling was done using Stata’s graph editor) of the figures in this paper. An Excel file with climate normals for each state as well as positive case and death count daily TRP constrained at 5°C and 2°C is also included.

**Supplemental Material Figures**

**Figure S1.** DailyDead TRP contour plot representation.


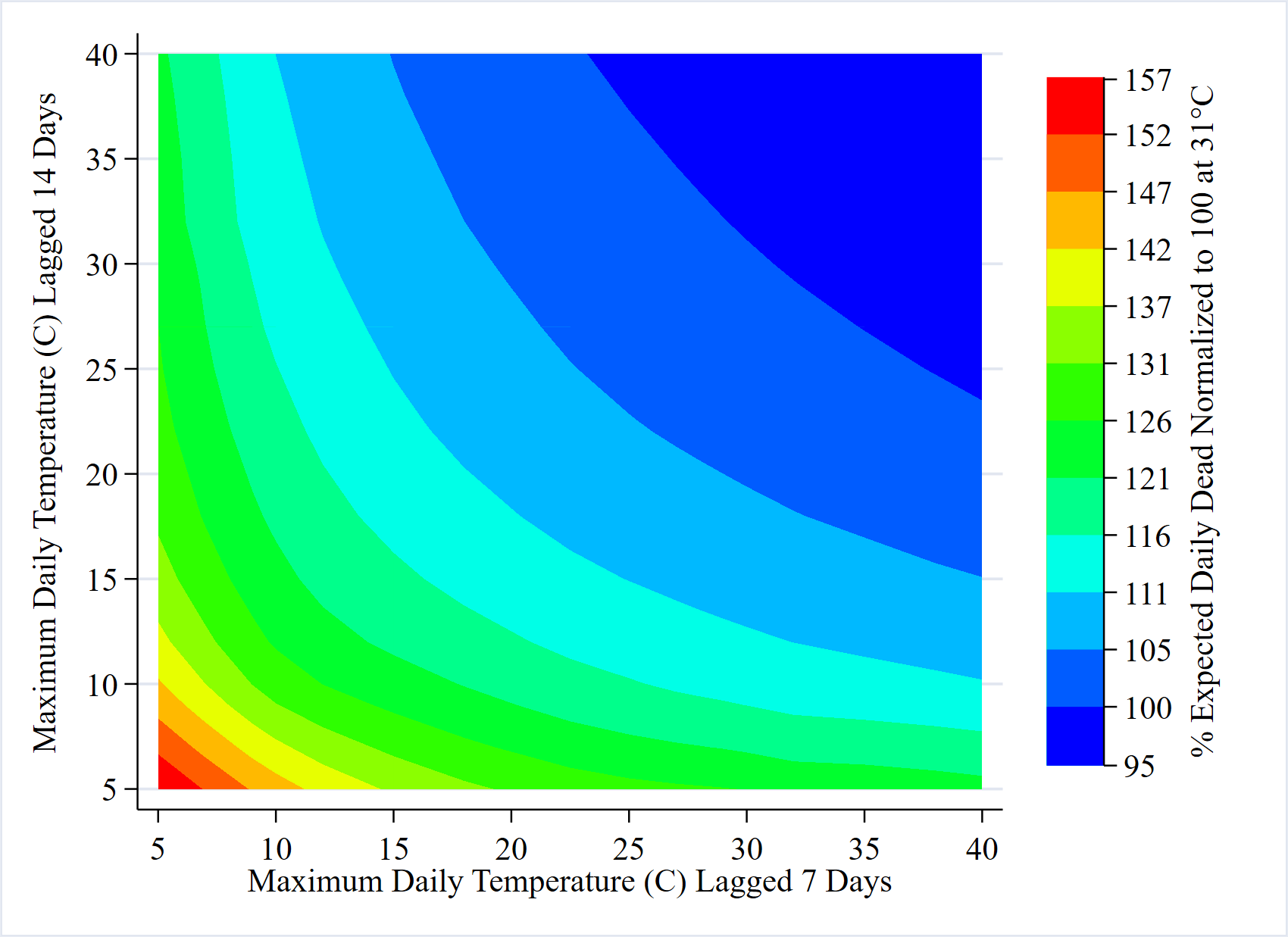


**Figure S2.** NewPositives TRP contour plot representation.


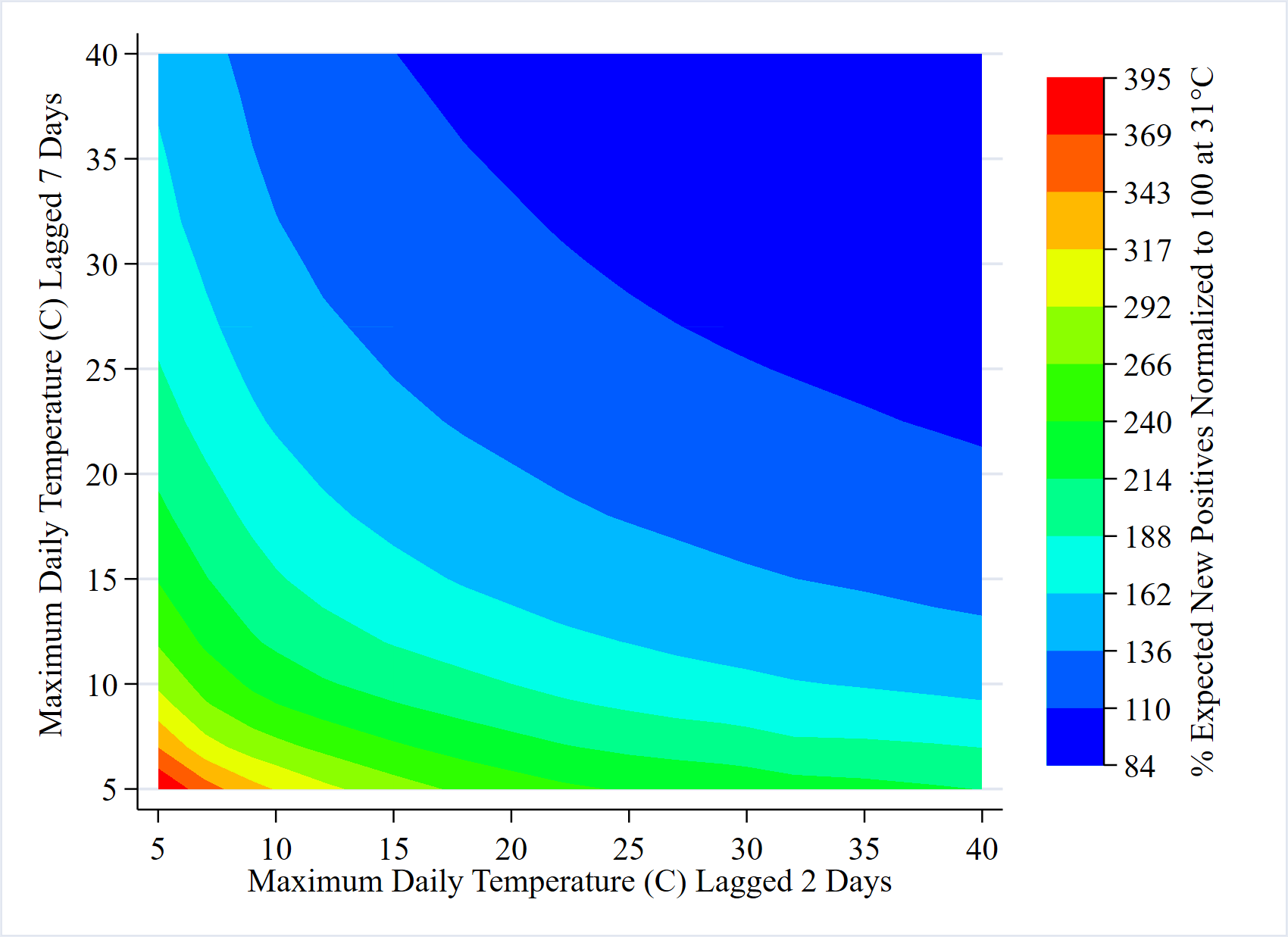


**Figure S3.** LogMaxTemp vs. LogUV. **(A)** displays Georgia. **(B)** displays New York.


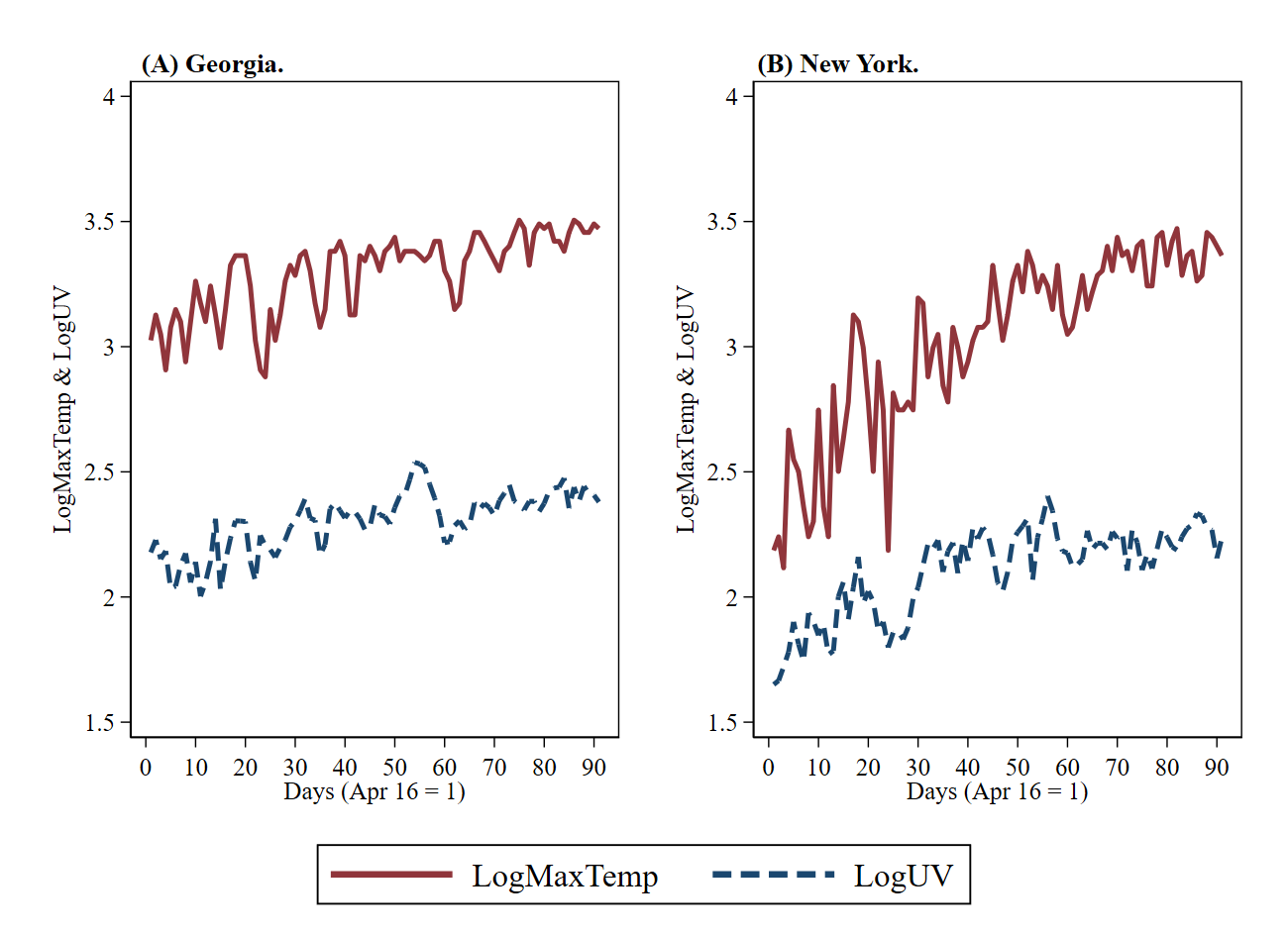


**Figure S4.** DailyDead_it_ responsiveness to lagged cumulative per capita death counts.


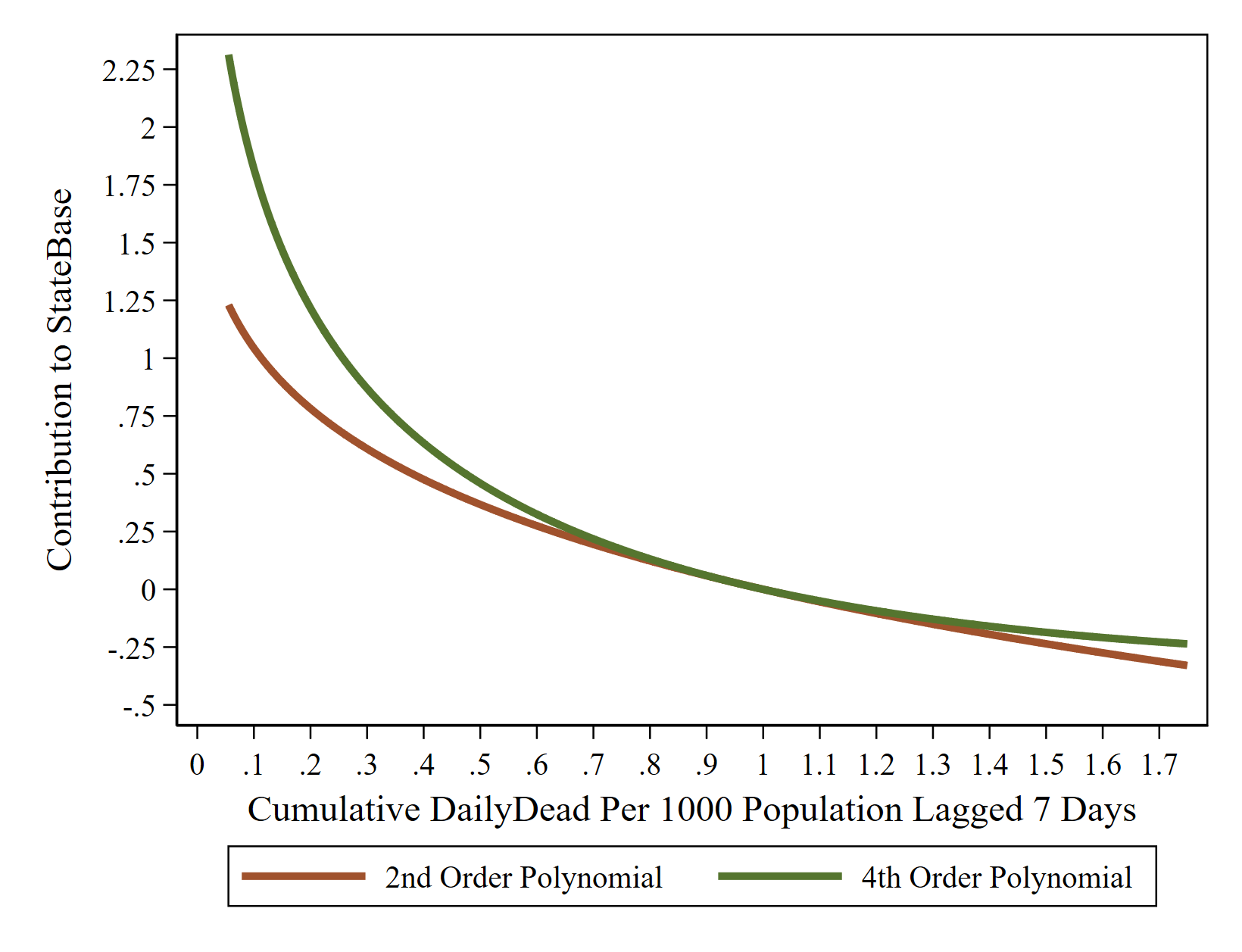


**Figure S5.** Bivariate relationship DailyDead & MaxTemp. U.S. states: April 16-July 15. Lowess smoother: .2 bandwidth.


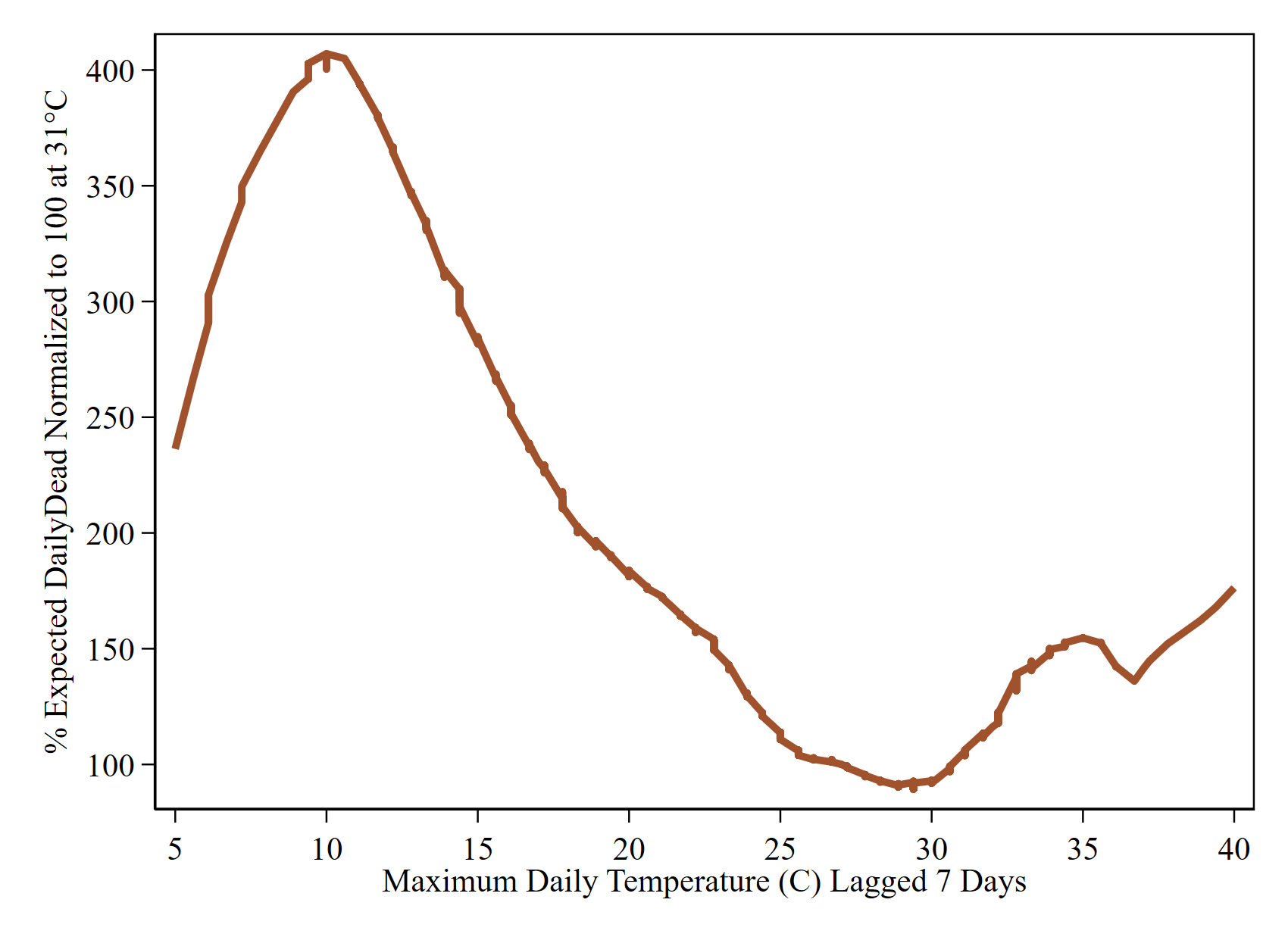


**Figure S6.** Death count TRPs based on DailyDead vs. CTP originally reported. U.S. states: April 16-July 15.


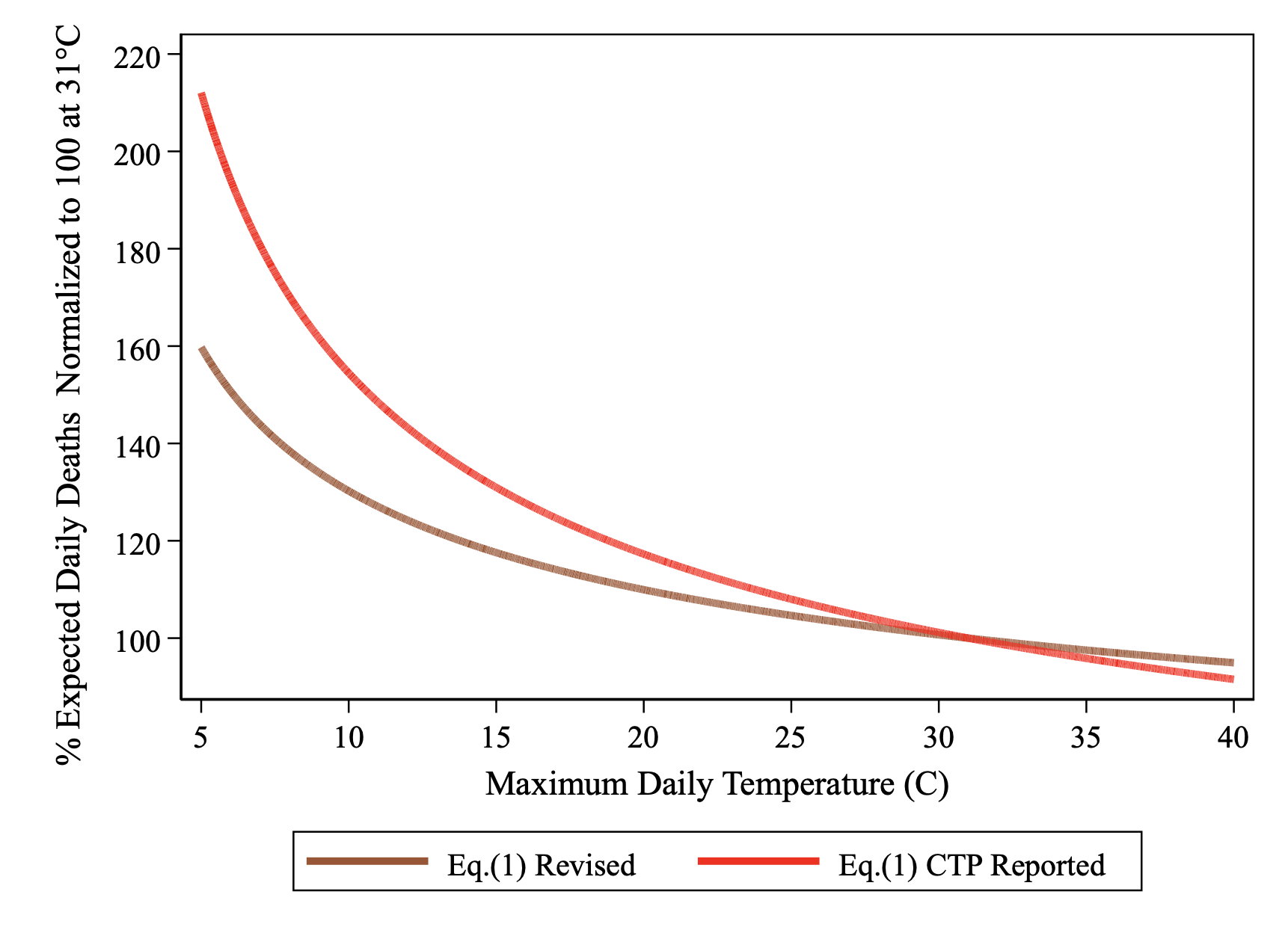


**Supplemental Materials Tables**

**Table S1. Base model 1 [Eq. 1] predicting DailyDead_it_.**

| **Variable** | **Coefficient** | **Robust S.E.** | **t-statistic** | **p-value** |
| --- | --- | --- | --- | --- |
| Alaska | 23.8933 | 3.1314 | 7.63 | 0.000 |
| Alabama | 26.1698 | 3.1149 | 8.40 | 0.000 |
| Arkansas | 25.5277 | 3.1144 | 8.20 | 0.000 |
| Arizona | 26.5129 | 3.0962 | 8.56 | 0.000 |
| California | 26.4774 | 3.1081 | 8.52 | 0.000 |
| Colorado | 26.0548 | 3.1209 | 8.35 | 0.000 |
| Connecticut | 26.2140 | 3.1197 | 8.40 | 0.000 |
| District of Columbia | 25.9150 | 3.1306 | 8.28 | 0.000 |
| Delaware | 25.9414 | 3.1330 | 8.28 | 0.000 |
| Florida | 26.5935 | 3.0917 | 8.60 | 0.000 |
| Georgia | 26.3336 | 3.1171 | 8.45 | 0.000 |
| Hawaii | 23.7736 | 3.1274 | 7.60 | 0.000 |
| Iowa | 26.0511 | 3.1298 | 8.32 | 0.000 |
| Idaho | 25.2141 | 3.1238 | 8.07 | 0.000 |
| Illinois | 26.4075 | 3.1210 | 8.46 | 0.000 |
| Indiana | 26.2014 | 3.1238 | 8.39 | 0.000 |
| Kansas | 25.5610 | 3.1236 | 8.18 | 0.000 |
| Kentucky | 25.7111 | 3.1237 | 8.23 | 0.000 |
| Louisiana | 26.2047 | 3.1147 | 8.41 | 0.000 |
| Massachusetts | 26.3124 | 3.1192 | 8.44 | 0.000 |
| Maryland | 26.3021 | 3.1236 | 8.42 | 0.000 |
| Maine | 24.8825 | 3.1301 | 7.95 | 0.000 |
| Michigan | 26.1080 | 3.1136 | 8.39 | 0.000 |
| Minnesota | 26.1769 | 3.1285 | 8.37 | 0.000 |
| Missouri | 26.0599 | 3.1240 | 8.34 | 0.000 |
| Mississippi | 26.1877 | 3.1168 | 8.40 | 0.000 |
| Montana | 24.6468 | 3.1300 | 7.87 | 0.000 |
| North Carolina | 26.2045 | 3.1192 | 8.40 | 0.000 |
| North Dakota | 25.1081 | 3.1315 | 8.02 | 0.000 |
| Nebraska | 25.6150 | 3.1247 | 8.20 | 0.000 |
| New Hampshire | 25.7766 | 3.1305 | 8.23 | 0.000 |
| New Jersey | 26.2906 | 3.1101 | 8.45 | 0.000 |
| New Mexico | 25.9870 | 3.1267 | 8.31 | 0.000 |
| Nevada | 26.0207 | 3.1178 | 8.35 | 0.000 |
| New York | 26.1870 | 3.1047 | 8.43 | 0.000 |
| Ohio | 26.2321 | 3.1239 | 8.40 | 0.000 |
| Oklahoma | 25.7736 | 3.1245 | 8.25 | 0.000 |
| Oregon | 25.5651 | 3.1216 | 8.19 | 0.000 |
| Pennsylvania | 26.3668 | 3.1197 | 8.45 | 0.000 |
| Rhode Island | 26.0353 | 3.1304 | 8.32 | 0.000 |
| South Carolina | 26.3046 | 3.1088 | 8.46 | 0.000 |
| South Dakota | 25.3250 | 3.1267 | 8.10 | 0.000 |
| Tennessee | 25.9479 | 3.1130 | 8.34 | 0.000 |
| Texas | 26.7452 | 3.0915 | 8.65 | 0.000 |
| Utah | 25.6526 | 3.1183 | 8.23 | 0.000 |
| Virginia | 26.2141 | 3.1259 | 8.39 | 0.000 |
| Vermont | 24.2220 | 3.1289 | 7.74 | 0.000 |
| Washington | 25.9565 | 3.1198 | 8.32 | 0.000 |
| Wisconsin | 25.8251 | 3.1249 | 8.26 | 0.000 |
| West Virginia | 25.1182 | 3.1324 | 8.02 | 0.000 |
| Wyoming | 23.8756 | 3.1321 | 7.62 | 0.000 |
| LogDays_t_ | -10.3772 | 1.4614 | -7.10 | 0.000 |
| LogDays_t_*LogDays_t_ | 1.1834 | 0.1696 | 6.98 | 0.000 |
| LogDailyDead_it-7_ | 0.8670 | 0.0277 | 31.28 | 0.000 |
| LogMaxTemp_it-7_ | 0.3110 | 0.0448 | 6.95 | 0.000 |
| LogMaxTemp_it-14_ | 0.2945 | 0.0516 | 5.71 | 0.000 |
|  |  |  |  |  |
| R^2^ | 0.9711 |  |  |  |
| Root MSE | 8.5430 |  |  |  |
| Observations | 4567 |  |  |  |

**Table S2. Model predicting NewPositives_it_.**

| **Variable** | **Coefficient** | **Robust S.E.** | **t-statistic** | **p-value** |
| --- | --- | --- | --- | --- |
| Alaska | 44.3754 | 7.8220 | 5.67 | 0.000 |
| Alabama | 45.0719 | 7.7439 | 5.82 | 0.000 |
| Arkansas | 45.0296 | 7.7613 | 5.80 | 0.000 |
| Arizona | 45.4897 | 7.7481 | 5.87 | 0.000 |
| California | 45.2471 | 7.8135 | 5.79 | 0.000 |
| Colorado | 44.7237 | 7.7036 | 5.81 | 0.000 |
| Connecticut | 44.9710 | 7.8123 | 5.76 | 0.000 |
| District of Columbia | 44.8480 | 7.7886 | 5.76 | 0.000 |
| Delaware | 44.8834 | 7.7766 | 5.77 | 0.000 |
| Florida | 45.7236 | 7.8081 | 5.86 | 0.000 |
| Georgia | 45.2116 | 7.7504 | 5.83 | 0.000 |
| Hawaii | 43.5360 | 7.5748 | 5.75 | 0.000 |
| Iowa | 44.9210 | 7.7604 | 5.79 | 0.000 |
| Idaho | 44.4862 | 7.6268 | 5.83 | 0.000 |
| Illinois | 45.2043 | 7.8320 | 5.777 | 0.000 |
| Indiana | 44.7918 | 7.7378 | 5.79 | 0.000 |
| Kansas | 44.8676 | 7.6937 | 5.83 | 0.000 |
| Kentucky | 44.4355 | 7.7336 | 5.75 | 0.000 |
| Louisiana | 46.0003 | 7.9379 | 5.80 | 0.000 |
| Massachusetts | 45.0429 | 7.8516 | 5.74 | 0.000 |
| Maryland | 45.1639 | 7.8065 | 5.79 | 0.000 |
| Maine | 43.7347 | 7.6623 | 5.71 | 0.000 |
| Michigan | 44.6836 | 7.7889 | 5.74 | 0.000 |
| Minnesota | 44.8802 | 7.7795 | 5.77 | 0.000 |
| Missouri | 44.9420 | 7.7386 | 5.81 | 0.000 |
| Mississippi | 45.1696 | 7.7728 | 5.81 | 0.000 |
| Montana | 44.2548 | 7.6490 | 5.79 | 0.000 |
| North Carolina | 44.0362 | 7.549 | 5.81 | 0.000 |
| North Dakota | 44.4879 | 7.7784 | 5.72 | 0.000 |
| Nebraska | 44.8758 | 7.7590 | 5.78 | 0.000 |
| New Hampshire | 44.2714 | 7.7374 | 5.72 | 0.000 |
| New Jersey | 45.2459 | 7.8555 | 5.76 | 0.000 |
| New Mexico | 44.0833 | 7.8327 | 5.76 | 0.000 |
| Nevada | 45.1552 | 7.7471 | 5.83 | 0.000 |
| New York | 45.2907 | 7.8897 | 5.74 | 0.000 |
| Ohio | 44.6637 | 7.7159 | 5.79 | 0.000 |
| Oklahoma | 44.7409 | 7.7293 | 5.79 | 0.000 |
| Oregon | 43.9776 | 7.6495 | 5.75 | 0.000 |
| Pennsylvania | 44.8583 | 7.7437 | 5.79 | 0.000 |
| Rhode Island | 44.8480 | 7.8233 | 5.73 | 0.000 |
| South Carolina | 45.0818 | 7.7231 | 5.84 | 0.000 |
| South Dakota | 44.4405 | 7.7294 | 5.75 | 0.000 |
| Tennessee | 45.3233 | 7.8145 | 5.80 | 0.000 |
| Texas | 45.3508 | 7.7418 | 5.86 | 0.000 |
| Utah | 44.9916 | 7.7777 | 5.78 | 0.000 |
| Virginia | 45.0741 | 7.7745 | 5.80 | 0.000 |
| Vermont | 43.1094 | 7.6813 | 5.61 | 0.000 |
| Washington | 44.5409 | 7.7176 | 5.77 | 0.000 |
| Wisconsin | 44.7343 | 7.7540 | 5.77 | 0.000 |
| West Virginia | 44.1364 | 7.7090 | 5.73 | 0.000 |
| Wyoming | 43.7190 | 7.6626 | 5.71 | 0.000 |
| LogNewTests_it_ | 0.3855 | 0.0833 | 4.63 | 0.000 |
| MONDAY | -0.0801 | 0.0329 | -2.44 | 0.015 |
| LogDays_t_ | -19.5476 | 3.7152 | -5.62 | 0.000 |
| LogDays_t_*LogDays_t_ | 2.3839 | 0.4551 | 5.24 | 0.000 |
| LogNewPositives_it-7_ | 0.5856 | 0.0469 | 12.48 | 0.000 |
| LogNewTests_it-7_ | -0.1755 | 0.0584 | -2.93 | 0.005 |
| PerCapitaTests_it-7_ | -0.0096 | 0.0031 | -3.13 | 0.002 |
| LogMaxTemp_it-2_ | 0.5435 | 0.1082 | 4.94 | 0.000 |
| LogMaxTemp_it-7_ | 0.6873 | 0.0460 | 14.94 | 0.000 |
|  |  |  |  |  |
| R^2^ | 0.9507 |  |  |  |
| Root MSE | 303.503 |  |  |  |
| Observations | 4592 |  |  |  |

**Table S3. Alternative specifications for DailyDead_it_ model.** StateIndicator_i_ for models provided in Table S6. Robust standard errors clustered at the state level.

| **Variable** | **Model. 1**  Base Model | **Model. 3**  Linear MaxTemp | **Model. 4**  Ratio Scaler | **Model. 5**  Ratio Scaler  Linear MaxTemp | **Model. 6**  14^th^ Lag DailyDead | **Model. 7**  Rolling Weekly | **Model. 12**  Add  UV |
| --- | --- | --- | --- | --- | --- | --- | --- |
| LogDays_t_ | -10.3772 | -10.5134 | -10.1582 | -10.0802 | -29.2265 | -13.9999 | -9.0650 |
|  | (1.4614) | (1.4508) | (1.4960) | (1.5057) | (2.4812) | (1.5320) | (1.2291) |
| LogDays_t_*LogDays_t_ | 1.1834 | 1.2006 | 1.1560 | 1.1462 | 3.3075 | 1.5822 | 1.0460 |
|  | (0.1696) | (0.1683) | (0.1736) | (0.1748) | (0.2953) | (0.1787) | (0.1461) |
| LogDailyDead_it-7_ | 0.8670 | 0.8652 | 0.8716 | 0.8731 |  |  | 0.8585 |
|  | (0.0277 | (0.0283) | (0.0269) | (0.0267) |  |  | (0.0280) |
| LogDailyDead_it-14_ |  |  |  |  | 0.8049 |  |  |
|  |  |  |  |  | (0.1907) |  |  |
| LogWeeklyDead_it-7_ |  |  |  |  |  | 0.9204 |  |
|  |  |  |  |  |  | (0.0487) |  |
| LogMaxTemp_it-7_ | 0.3110 |  | -0.6064 |  | 0.1907 |  | 0.2010. |
|  | (0.0448) |  | (0.0922) |  | (0.1491) |  | (0.0981) |
| LogMaxTemp_it-14_ | 0.2945 |  | -0.5713 |  | 0.4566 |  | 0.2540 |
|  | (0.0516) |  | (0.0866) |  | (0.0667) |  | (0.0568) |
| MaxTemp_it-7_ |  | 0.1186 |  | -1.3784 |  |  |  |
|  |  | (0.0166) |  | (0.2482) |  |  |  |
| MaxTemp_it-14_ |  | 0.1132 |  | -1.2842 |  |  |  |
|  |  | (0.0206) |  | (0.2182) |  |  |  |
| LogMaxTemp_it-14_ |  |  |  |  |  | 0.2111 |  |
|  |  |  |  |  |  | (0.0498) |  |
| LogMaxTemp_it-18_ |  |  |  |  |  | -233.139 |  |
|  |  |  |  |  |  |  |  |
| LogUV_it-7_ |  |  |  |  |  |  | 0.4099 |
|  |  |  |  |  |  |  | (0.1729) |
| LogUV_it-7_ |  |  |  |  |  |  | 0.2463 |
|  |  |  |  |  |  |  | (0.1256) |
|  |  |  |  |  |  |  |  |
| R^2^ | 0.9711 | 0.9711 | 0.9709 | 0.9709 | 0.9473 | 0.9872 | 0.9714 |
| Root MSE | 8.5430 | 8.5357 | 8.5595 | 8.5684 | 11.5266 | 38.9815 | 8.4871 |
| Observations | 4567 | 4567 | 4567 | 4567 | 4576 | 4552 | 4567 |

**Table S4. AR(7)** **DailyDead_it_ models using different originally Reported and Revised death count combinations.** U.S. states: April 16-July 15. June 25 NJ death count (1877) set to missing in reported. (Robust standard errors clustered at state level).

|  | **Model 18** | **Model 19** | **Model 20** | **Model 21** |
| --- | --- | --- | --- | --- |
|  | Reported/Reported | Corrected/Reported | Reported/Corrected | Corrected/Corrected |
| DailyDead_it-7_ | 0.6986 | 0.6663 | 0.7434 | 0.7798 |
|  | (0.0069) | (0.0063) | (0.0063) | (0.0033) |
| Constant | 5.0046 | 6.4614 | 3.3406 | 3.1815 |
|  | (0.4210) | (0.3873) | (0.3820) | (0.1965) |
|  |  |  |  |  |
| R^2^ | 0.6893 | 0.7046 | 0.7484 | 0.9252 |
| RMSE | 26.545 | 24.419 | 23.885 | 12.286 |
| Observations | 4639 | 4639 | 4639 | 4639 |

**Table S5. State-level daily temperature response profile statistics.**

| **State** | **Constrained to 5C** | | | | | **Constrained to 2C** | | | | |  |
| --- | --- | --- | --- | --- | --- | --- | --- | --- | --- | --- | --- |
|  | **Mean** | **sd** | **median** | **min** | **max** | **Mean** | **sd** | **median** | **min** | **max** |  |
| Alabama | 130.4 | 33.1 | 119.1 | 95.8 | 195.4 | 130.4 | 33.1 | 119.1 | 95.8 | 195.4 | |
| Alaska | 283.4 | 110.2 | 314.1 | 140.9 | 392.6 | 491.8 | 335.7 | 314.1 | 140.9 | 880.1 | |
| Arizona | 105.8 | 21.2 | 101.0 | 81.6 | 144.8 | 105.8 | 21.2 | 101.0 | 81.6 | 144.8 | |
| Arkansas | 139.6 | 44.0 | 123.1 | 94.6 | 229.1 | 139.6 | 44.0 | 123.1 | 94.6 | 229.1 | |
| California | 121.8 | 28.8 | 115.1 | 88.2 | 175.8 | 121.8 | 28.8 | 115.1 | 88.2 | 175.8 | |
| Colorado | 177.7 | 78.0 | 153.4 | 96.8 | 329.1 | 177.7 | 78.0 | 153.4 | 96.8 | 329.1 | |
| Connecticut | 208.8 | 111.5 | 154.3 | 102.8 | 392.6 | 266.9 | 230.6 | 154.3 | 102.8 | 880.1 | |
| Delaware | 183.5 | 90.9 | 142.4 | 100.9 | 391.2 | 183.5 | 90.9 | 142.4 | 100.9 | 391.2 | |
| District of Columbia | 161.4 | 65.5 | 133.6 | 97.5 | 302.8 | 161.4 | 65.5 | 133.6 | 97.5 | 302.8 | |
| Florida | 106.2 | 7.4 | 104.6 | 96.9 | 119.3 | 106.2 | 7.4 | 104.6 | 96.9 | 119.3 | |
| Georgia | 133.0 | 33.9 | 121.9 | 96.9 | 198.5 | 133.0 | 33.9 | 121.9 | 96.9 | 198.5 | |
| Hawaii | 104.4 | 4.3 | 104.3 | 98.6 | 110.5 | 104.4 | 4.3 | 104.3 | 98.6 | 110.5 | |
| Idaho | 195.1 | 106.5 | 151.3 | 92.2 | 392.6 | 215.3 | 149.0 | 151.3 | 92.2 | 606.6 | |
| Illinois | 216.0 | 115.8 | 159.6 | 104.0 | 392.6 | 308.8 | 280.8 | 159.6 | 104.0 | 880.1 | |
| Indiana | 200.0 | 109.2 | 145.9 | 103.2 | 392.6 | 244.0 | 203.9 | 145.9 | 103.2 | 857.0 | |
| Iowa | 211.7 | 116.4 | 152.7 | 102.5 | 392.6 | 306.2 | 285.2 | 152.7 | 102.5 | 880.1 | |
| Kansas | 163.8 | 74.9 | 132.9 | 94.0 | 329.6 | 163.8 | 74.9 | 132.9 | 94.0 | 329.6 | |
| Kentucky | 189.9 | 100.8 | 141.1 | 102.2 | 392.6 | 195.3 | 112.5 | 141.1 | 102.2 | 470.1 | |
| Louisiana | 117.5 | 20.3 | 112.0 | 95.4 | 155.2 | 117.5 | 20.3 | 112.0 | 95.4 | 155.2 | |
| Maine | 229.4 | 113.9 | 178.1 | 111.1 | 392.6 | 321.3 | 273.1 | 178.1 | 111.1 | 880.1 | |
| Maryland | 170.0 | 75.6 | 137.5 | 98.3 | 337.8 | 170.0 | 75.6 | 137.5 | 98.3 | 337.8 | |
| Massachusetts | 214.3 | 107.9 | 165.1 | 107.3 | 392.6 | 249.1 | 178.4 | 165.1 | 107.3 | 745.4 | |
| Michigan | 218.0 | 115.8 | 161.6 | 105.1 | 392.6 | 311.5 | 280.7 | 161.6 | 105.1 | 880.1 | |
| Minnesota | 232.7 | 122.0 | 174.8 | 105.4 | 392.6 | 369.8 | 322.0 | 174.8 | 105.4 | 880.1 | |
| Mississippi | 124.4 | 28.1 | 115.7 | 94.3 | 178.4 | 124.4 | 28.1 | 115.7 | 94.3 | 178.4 | |
| Missouri | 179.2 | 95.7 | 134.1 | 97.6 | 392.6 | 181.4 | 100.6 | 134.1 | 97.6 | 435.2 | |
| Montana | 219.0 | 113.9 | 175.1 | 98.2 | 392.6 | 293.0 | 248.4 | 175.1 | 98.2 | 880.1 | |
| Nebraska | 202.9 | 114.3 | 145.8 | 99.3 | 392.6 | 279.9 | 264.2 | 145.8 | 99.3 | 880.1 | |
| Nevada | 120.0 | 32.8 | 112.0 | 83.2 | 183.7 | 120.0 | 32.8 | 112.0 | 83.2 | 183.7 | |
| New Hampshire | 215.6 | 113.8 | 159.9 | 104.4 | 392.6 | 287.9 | 252.1 | 159.9 | 104.4 | 880.1 | |
| New Jersey | 191.1 | 98.4 | 146.9 | 100.7 | 392.6 | 194.7 | 106.2 | 146.9 | 100.7 | 451.5 | |
| New Mexico | 150.3 | 56.3 | 130.1 | 94.9 | 275.5 | 150.3 | 56.3 | 130.1 | 94.9 | 275.5 | |
| New York | 199.9 | 100.0 | 156.4 | 105.1 | 392.6 | 206.5 | 113.8 | 156.4 | 105.1 | 483.8 | |
| North Carolina | 136.8 | 37.5 | 123.3 | 96.8 | 210.9 | 136.8 | 37.5 | 123.3 | 96.8 | 210.9 | |
| North Dakota | 241.9 | 124.8 | 187.6 | 107.1 | 392.6 | 403.4 | 337.6 | 187.6 | 107.1 | 880.1 | |
| Ohio | 210.3 | 111.9 | 155.0 | 105.1 | 392.6 | 265.3 | 222.8 | 155.0 | 105.1 | 880.1 | |
| Oklahoma | 144.4 | 49.2 | 127.0 | 92.7 | 245.5 | 144.4 | 49.2 | 127.0 | 92.7 | 245.5 | |
| Oregon | 167.1 | 54.2 | 152.5 | 105.5 | 276.4 | 167.1 | 54.2 | 152.5 | 105.5 | 276.4 | |
| Pennsylvania | 182.5 | 91.3 | 141.8 | 99.6 | 392.6 | 182.5 | 91.5 | 141.8 | 99.6 | 394.7 | |
| Rhode Island | 204.8 | 104.5 | 156.5 | 105.0 | 392.6 | 220.3 | 136.2 | 156.5 | 105.0 | 564.9 | |
| South Carolina | 121.7 | 22.9 | 115.1 | 95.6 | 164.1 | 121.7 | 22.9 | 115.1 | 95.6 | 164.1 | |
| South Dakota | 222.7 | 120.4 | 163.3 | 102.8 | 392.6 | 342.6 | 309.9 | 163.3 | 102.8 | 880.1 | |
| Tennessee | 142.7 | 47.3 | 123.7 | 96.1 | 241.2 | 142.7 | 47.3 | 123.7 | 96.1 | 241.2 | |
| Texas | 125.4 | 31.3 | 116.3 | 89.8 | 185.7 | 125.4 | 31.3 | 116.3 | 89.8 | 185.7 | |
| Utah | 194.3 | 107.7 | 151.6 | 91.5 | 392.6 | 215.0 | 150.7 | 151.6 | 91.5 | 610.9 | |
| Vermont | 230.3 | 119.5 | 175.1 | 106.9 | 392.6 | 348.2 | 303.9 | 175.1 | 106.9 | 880.1 | |
| Virginia | 173.8 | 80.0 | 137.8 | 100.0 | 352.4 | 173.8 | 80.0 | 137.8 | 100.0 | 352.4 | |
| Washington | 174.8 | 50.8 | 165.3 | 112.9 | 270.4 | 174.8 | 50.8 | 165.3 | 112.9 | 270.4 | |
| West Virginia | 166.0 | 70.3 | 132.5 | 102.0 | 321.4 | 166.0 | 70.3 | 132.5 | 102.0 | 321.4 | |
| Wisconsin | 225.0 | 115.6 | 172.8 | 107.6 | 392.6 | 326.3 | 287.3 | 172.8 | 107.6 | 880.1 | |
| Wyoming | 249.3 | 118.1 | 210.0 | 109.6 | 392.6 | 399.2 | 322.8 | 210.0 | 109.6 | 880.1 | |

**Structure of Table S6**

The parameter estimates and standard summary statistics for models discussed is this paper are provided in Table S6. It contains three columns for each model. The first column contains the variables included in the model, the second the parameter estimates, and the third the standard errors. After the parameter estimates, the model’s R^2^, root mean square error (RMSE), and the number of observations on which the model was fit are provided.

The order in which the models appear are:

1. The base model represented by Eq. 1 using LogDays_t_, LogDays_t_^2^, and LogDailyDead_it-7_ as the predictors along with a set of state-level indicator variables and using LogMaxTemp_it-7_ and LogMaxTemp_it-14_ in the temperature scaling function.

2. Model regressing the squared residuals from (1) on the log of state population and an indicator variable, GOOD_DATE for DailyDead_it_ representing death counts by death certificate date.

3. Base model using linear versions of the two temperature variables.

4. Base model using alternative ratio scaling function Temp/(Temp + α), where Temp is temperature variable and α is the estimated parameter.

5. Base model using the alternative ratio scaling function and linear versions of the MaxTemp_it-k_.

6. Base model with LogDailyDead_it-14_ substituted for LogDailyDead_it-7_.

7. A weekly version of the base model (see Eq. 2) substituting LogDailyDead_it-14_ for LogDailyDead_it-7_ and LogDailyDead_it-18_ for LogDailyDead_it-14_.

8. A version of the base model that adds two state-level government policy variables, the log of the number of days since a shelter-in-place order was first issued (LogDaysShelterInPlace_it_) and the log of the number of days since a state began to formally reopen its economy (LogDaysReopen_it_).

9. A version of (8) that drops the quadratic time trend of the base model.

10. The base model adding parallel scaling functions using the LogMaxAbsoluteHumidity_it-7_ and LogMaxAbsoluteHumidity_it-14_.

11. The base model adding parallel scaling functions LogMaxRelativeHumidity_it-7_ and LogMaxRelativeHumidity_it-7._

12. Base model adding additional parallel scaling functions for LogUV_it-7_ and LogUV_it-14_.

13. Base model substituting LogUV_it-k_ for LogMaxTemp_it-k_.

14. Base modeling adding a quadratic in terms of LogTotalDead_it-7_.

15. Base modeling adding a 4th order polynomial LogTotalDead_it-7_.

16. Base model substituting LogNewPositives_it-7_ and testing variables for LogDailyDead_it-7_.

17. The model used in the paper to predict NewPositives_it_.

18. The AR(7) regression of the originally reported death counts from the COVID Tracking Project (CPTDailyDead_it_) on the 7^th^ lag of itself (CPTDailyDead_it-7_) for Table S4.

19. The same as (18) but with DailyDead_it_ regressed on the 7^th^ lag of the CTPDailyDead_it_.

20. The same as (18) but CTPDailyDead_it_ regressed on the 7^th^ lag of DailyDead_it_.

21. The same as (18) but DailyDead_it_ regressed on its 7^th^ lag.

22. The StateBase_it_ calculation for each state.

23. The model reported in Table 2 which predicts the state-level fixed-effect estimates obtained in (1) as a function of a small set of demographic variables.

24. State-level estimates of the minimum infection pool from (1) obtained by setting DailyDead_it-7_ = 0 and assuming infection fatality rate of 0.65% from https://www.cdc.gov/coronavirus/2019-ncov/ hcp/planning-scenarios.html, retrieved 30 June 2020.

25. State-level change in expected DailyDead_it_ when DailyDead_it-7_ shifts from 0 to 1 on July 15, assuming 31°C.

26. Base model (1) using the uncorrected (CovidTracking.com) death count data.

27. Base model (1) adding the SafeGraph mobility data lagged 11 days.

28. Base model (1) substituting LogMaxTemp_it_ with the population weighted centroid temperature data LogCMaxTemp_it_.

**Table S6. Parameter estimates including state-level fixed effects for all models.**

| **Model 1** |  |  |  | **Model 2** |  |  |
| --- | --- | --- | --- | --- | --- | --- |
| Variable | Coefficient | S.E. |  | Variable | Coefficient | S.E. |
| State_AK | 23.8933 | 3.1314 |  | LogPopulation | 83.8847 | 4.2834 |
| State_AL | 26.1698 | 3.1149 |  | Good_Dates | -47.062 | 9.213 |
| State_AR | 25.5277 | 3.1144 |  | _cons | -591.045 | 34.3825 |
| State_AZ | 26.5128 | 3.0962 |  |  |  |  |
| State_CA | 26.4774 | 3.1081 |  | R-square | 0.0777 |  |
| State_CO | 26.0547 | 3.1209 |  | Root MSE | 282.8758 |  |
| State_CT | 26.214 | 3.1197 |  | Observations | 4567 |  |
| State_DC | 25.915 | 3.1306 |  |  |  |  |
| State_DE | 25.9414 | 3.133 |  |  |  |  |
| State_FL | 26.593 | 3.0917 |  |  |  |  |
| State_GA | 26.3335 | 3.1171 |  |  |  |  |
| State_HI | 23.7735 | 3.1274 |  |  |  |  |
| State_IA | 26.0511 | 3.1298 |  |  |  |  |
| State_ID | 25.2141 | 3.1238 |  |  |  |  |
| State_IL | 26.4075 | 3.121 |  |  |  |  |
| State_IN | 26.2014 | 3.1238 |  |  |  |  |
| State_KS | 25.561 | 3.1236 |  |  |  |  |
| State_KY | 25.7111 | 3.1237 |  |  |  |  |
| State_LA | 26.2046 | 3.1147 |  |  |  |  |
| State_MA | 26.3124 | 3.1192 |  |  |  |  |
| State_MD | 26.3021 | 3.1236 |  |  |  |  |
| State_ME | 24.8825 | 3.1301 |  |  |  |  |
| State_MI | 26.108 | 3.1136 |  |  |  |  |
| State_MN | 26.1768 | 3.1285 |  |  |  |  |
| State_MO | 26.0599 | 3.124 |  |  |  |  |
| State_MS | 26.1877 | 3.1168 |  |  |  |  |
| State_MT | 24.6467 | 3.13 |  |  |  |  |
| State_NC | 26.2044 | 3.1192 |  |  |  |  |
| State_ND | 25.1081 | 3.1315 |  |  |  |  |
| State_NE | 25.6149 | 3.1247 |  |  |  |  |
| State_NH | 25.7766 | 3.1305 |  |  |  |  |
| State_NJ | 26.2905 | 3.1101 |  |  |  |  |
| State_NM | 25.987 | 3.1267 |  |  |  |  |
| State_NV | 26.0206 | 3.1178 |  |  |  |  |
| State_NY | 26.1869 | 3.1047 |  |  |  |  |
| State_OH | 26.2321 | 3.1239 |  |  |  |  |
| State_OK | 25.7735 | 3.1245 |  |  |  |  |
| State_OR | 25.565 | 3.1216 |  |  |  |  |
| State_PA | 26.3667 | 3.1197 |  |  |  |  |
| State_RI | 26.0352 | 3.1304 |  |  |  |  |
| State_SC | 26.3045 | 3.1088 |  |  |  |  |
| State_SD | 25.3249 | 3.1267 |  |  |  |  |
| State_TN | 25.9478 | 3.113 |  |  |  |  |
| State_TX | 26.7451 | 3.0915 |  |  |  |  |
| State_UT | 25.6526 | 3.1183 |  |  |  |  |
| State_VA | 26.214 | 3.1259 |  |  |  |  |
| State_VT | 24.222 | 3.1289 |  |  |  |  |
| State_WA | 25.9564 | 3.1198 |  |  |  |  |
| State_WI | 25.8251 | 3.1249 |  |  |  |  |
| State_WV | 25.1181 | 3.1324 |  |  |  |  |
| State_WY | 23.8756 | 3.1321 |  |  |  |  |
| t0 [LogDays] | -10.3772 | 1.4614 |  |  |  |  |
| t2 [LogDays^2] | 1.1833 | 0.1696 |  |  |  |  |
| b1 [L7.DailyDead] | 0.8670 | 0.0277 |  |  |  |  |
| w1 [L7.LogMaxTemp] | 0.3110 | 0.0448 |  |  |  |  |
| w2 [L14.LogMaxTemp] | 0.2945 | 0.0516 |  |  |  |  |
|  |  |  |  |  |  |  |
| R-square | 0.9711 |  |  |  |  |  |
| Root MSE | 8.54299 |  |  |  |  |  |
| Observations | 4567 |  |  |  |  |  |
| **Model 3** |  |  |  | **Model 4** |  |  |
| Variable | Coefficient | S.E. |  | Variable | Coefficient | S.E. |
| State_AK | 24.1894 | 3.1105 |  | State_AK | 19.7989 | 3.2261 |
| State_AL | 26.4871 | 3.0949 |  | State_AL | 22.0379 | 3.2065 |
| State_AR | 25.8456 | 3.094 |  | State_AR | 21.3966 | 3.2068 |
| State_AZ | 26.8561 | 3.0766 |  | State_AZ | 22.3503 | 3.1868 |
| State_CA | 26.7855 | 3.0888 |  | State_CA | 22.3551 | 3.1984 |
| State_CO | 26.3678 | 3.1008 |  | State_CO | 21.9269 | 3.2123 |
| State_CT | 26.5217 | 3.1002 |  | State_CT | 22.0978 | 3.211 |
| State_DC | 26.2224 | 3.1102 |  | State_DC | 21.7987 | 3.2229 |
| State_DE | 26.2461 | 3.1126 |  | State_DE | 21.8302 | 3.2255 |
| State_FL | 26.924 | 3.0727 |  | State_FL | 22.4424 | 3.1813 |
| State_GA | 26.6499 | 3.0972 |  | State_GA | 22.2013 | 3.2079 |
| State_HI | 24.0935 | 3.1058 |  | State_HI | 19.6424 | 3.2213 |
| State_IA | 26.3616 | 3.1096 |  | State_IA | 21.9292 | 3.2223 |
| State_ID | 25.5187 | 3.1028 |  | State_ID | 21.1049 | 3.2171 |
| State_IL | 26.7207 | 3.1017 |  | State_IL | 22.2791 | 3.2116 |
| State_IN | 26.5115 | 3.1039 |  | State_IN | 22.0804 | 3.2153 |
| State_KS | 25.8761 | 3.1029 |  | State_KS | 21.4358 | 3.2162 |
| State_KY | 26.0181 | 3.1036 |  | State_KY | 21.5961 | 3.216 |
| State_LA | 26.5312 | 3.0945 |  | State_LA | 22.0579 | 3.2051 |
| State_MA | 26.623 | 3.0999 |  | State_MA | 22.1903 | 3.2103 |
| State_MD | 26.612 | 3.1039 |  | State_MD | 22.1803 | 3.2146 |
| State_ME | 25.1868 | 3.1093 |  | State_ME | 20.7746 | 3.2241 |
| State_MI | 26.419 | 3.0941 |  | State_MI | 21.9829 | 3.2051 |
| State_MN | 26.4845 | 3.1086 |  | State_MN | 22.0594 | 3.2204 |
| State_MO | 26.372 | 3.1038 |  | State_MO | 21.9366 | 3.2157 |
| State_MS | 26.5081 | 3.0966 |  | State_MS | 22.0519 | 3.2086 |
| State_MT | 24.9503 | 3.1089 |  | State_MT | 20.5389 | 3.2239 |
| State_NC | 26.5201 | 3.0992 |  | State_NC | 22.0746 | 3.2108 |
| State_ND | 25.418 | 3.1108 |  | State_ND | 20.9895 | 3.2252 |
| State_NE | 25.9292 | 3.1043 |  | State_NE | 21.4896 | 3.2172 |
| State_NH | 26.0835 | 3.1101 |  | State_NH | 21.6628 | 3.2235 |
| State_NJ | 26.6003 | 3.0911 |  | State_NJ | 22.1681 | 3.1995 |
| State_NM | 26.3079 | 3.106 |  | State_NM | 21.8522 | 3.2192 |
| State_NV | 26.3483 | 3.0969 |  | State_NV | 21.8778 | 3.2101 |
| State_NY | 26.4961 | 3.086 |  | State_NY | 22.0656 | 3.1936 |
| State_OH | 26.541 | 3.1042 |  | State_OH | 22.1123 | 3.2157 |
| State_OK | 26.0908 | 3.1039 |  | State_OK | 21.6433 | 3.2168 |
| State_OR | 25.8687 | 3.1009 |  | State_OR | 21.4553 | 3.2147 |
| State_PA | 26.6756 | 3.1003 |  | State_PA | 22.2464 | 3.21 |
| State_RI | 26.3401 | 3.1102 |  | State_RI | 21.9243 | 3.2231 |
| State_SC | 26.6242 | 3.0888 |  | State_SC | 22.1704 | 3.2005 |
| State_SD | 25.6346 | 3.106 |  | State_SD | 21.2081 | 3.2202 |
| State_TN | 26.2643 | 3.0928 |  | State_TN | 21.8186 | 3.2049 |
| State_TX | 27.0718 | 3.0725 |  | State_TX | 22.5997 | 3.1813 |
| State_UT | 25.9635 | 3.0978 |  | State_UT | 21.5337 | 3.2112 |
| State_VA | 26.5225 | 3.106 |  | State_VA | 22.095 | 3.2173 |
| State_VT | 24.5342 | 3.1077 |  | State_VT | 20.0972 | 3.2248 |
| State_WA | 26.2598 | 3.0996 |  | State_WA | 21.8458 | 3.2116 |
| State_WI | 26.1328 | 3.1047 |  | State_WI | 21.7098 | 3.2181 |
| State_WV | 25.4241 | 3.1115 |  | State_WV | 21.0064 | 3.2257 |
| State_WY | 24.1743 | 3.1111 |  | State_WY | 19.7744 | 3.2268 |
| t0 [LogDays] | -10.5134 | 1.4508 |  | t0 [LogDays] | -10.1582 | 1.496 |
| t2 [LogDays^2] | 1.2006 | 0.1683 |  | t2 [LogDays^2] | 1.156 | 0.1736 |
| b1 [L7.DailyDead] | 0.8652 | 0.0283 |  | b1 [L7.DailyDead] | 0.8716 | 0.0269 |
| k1 [L7.MaxTemp] | 0.1186 | 0.0166 |  | a1 [L7.LogMaxTempRatio] | -0.6064 | 0.0922 |
| k2 [L14.MaxTemp] | 0.1132 | 0.0206 |  | a2 [L.14LogMaxTempRatio] | -0.5713 | 0.0866 |
|  |  |  |  |  |  |  |
| R-square | 0.9711 |  |  | R-square | 0.9709 |  |
| Root MSE | 8.5357 |  |  | Root MSE | 8.5595 |  |
| Observations | 4567 |  |  | Observations | 4567 |  |

| **Model 5** |  |  |  | **Model 6** |  |  |
| --- | --- | --- | --- | --- | --- | --- |
| Variable | Coefficient | S.E. |  | Variable | Coefficient | S.E. |
| State_AK | 19.9478 | 3.2387 |  | State_AK | 65.8251 | 5.1475 |
| State_AL | 22.1737 | 3.2192 |  | State_AL | 68.061 | 5.1403 |
| State_AR | 21.5327 | 3.2195 |  | State_AR | 67.3279 | 5.1244 |
| State_AZ | 22.4755 | 3.1994 |  | State_AZ | 68.5349 | 5.1144 |
| State_CA | 22.4943 | 3.2109 |  | State_CA | 68.4365 | 5.1459 |
| State_CO | 22.0654 | 3.2251 |  | State_CO | 67.809 | 5.19 |
| State_CT | 22.2389 | 3.2236 |  | State_CT | 68.1275 | 5.1979 |
| State_DC | 21.9397 | 3.2357 |  | State_DC | 67.7794 | 5.1833 |
| State_DE | 21.9728 | 3.2383 |  | State_DE | 67.8381 | 5.1874 |
| State_FL | 22.5717 | 3.1937 |  | State_FL | 68.5752 | 5.1189 |
| State_GA | 22.337 | 3.2207 |  | State_GA | 68.1592 | 5.1688 |
| State_HI | 19.7783 | 3.2346 |  | State_HI | 65.5373 | 5.164 |
| State_IA | 22.0689 | 3.235 |  | State_IA | 67.8723 | 5.1683 |
| State_ID | 21.2483 | 3.23 |  | State_ID | 66.6162 | 5.1773 |
| State_IL | 22.4168 | 3.2241 |  | State_IL | 68.3871 | 5.1781 |
| State_IN | 22.22 | 3.228 |  | State_IN | 68.0356 | 5.1836 |
| State_KS | 21.5737 | 3.2292 |  | State_KS | 67.1362 | 5.1709 |
| State_KY | 21.7375 | 3.2287 |  | State_KY | 67.5074 | 5.1713 |
| State_LA | 22.1887 | 3.2181 |  | State_LA | 67.9049 | 5.18 |
| State_MA | 22.3303 | 3.2228 |  | State_MA | 68.321 | 5.1973 |
| State_MD | 22.3194 | 3.2273 |  | State_MD | 68.2591 | 5.1808 |
| State_ME | 20.9191 | 3.2368 |  | State_ME | 66.7611 | 5.1843 |
| State_MI | 22.1223 | 3.2179 |  | State_MI | 67.88 | 5.1921 |
| State_MN | 22.2001 | 3.233 |  | State_MN | 68.0622 | 5.1707 |
| State_MO | 22.0752 | 3.2286 |  | State_MO | 67.8882 | 5.1769 |
| State_MS | 22.1865 | 3.2214 |  | State_MS | 68.1112 | 5.1551 |
| State_MT | 20.6827 | 3.2367 |  | State_MT | 66.4531 | 5.1578 |
| State_NC | 22.2111 | 3.2235 |  | State_NC | 68.0884 | 5.1536 |
| State_ND | 21.1307 | 3.238 |  | State_ND | 66.7992 | 5.1669 |
| State_NE | 21.6275 | 3.2299 |  | State_NE | 67.3138 | 5.1449 |
| State_NH | 21.8048 | 3.2362 |  | State_NH | 67.4967 | 5.1648 |
| State_NJ | 22.307 | 3.212 |  | State_NJ | 68.1408 | 5.1939 |
| State_NM | 21.9869 | 3.2322 |  | State_NM | 67.8389 | 5.158 |
| State_NV | 22.0095 | 3.2232 |  | State_NV | 67.7397 | 5.1692 |
| State_NY | 22.2048 | 3.2061 |  | State_NY | 67.9975 | 5.1948 |
| State_OH | 22.2527 | 3.2284 |  | State_OH | 68.15 | 5.1795 |
| State_OK | 21.7801 | 3.2298 |  | State_OK | 67.5243 | 5.1817 |
| State_OR | 21.5986 | 3.2275 |  | State_OR | 67.156 | 5.1598 |
| State_PA | 22.386 | 3.2226 |  | State_PA | 68.3417 | 5.1932 |
| State_RI | 22.0673 | 3.2358 |  | State_RI | 67.928 | 5.1884 |
| State_SC | 22.3055 | 3.2131 |  | State_SC | 68.165 | 5.134 |
| State_SD | 21.3491 | 3.233 |  | State_SD | 67.0871 | 5.153 |
| State_TN | 21.9553 | 3.2176 |  | State_TN | 67.6737 | 5.149 |
| State_TX | 22.731 | 3.1936 |  | State_TX | 68.8406 | 5.1212 |
| State_UT | 21.6738 | 3.2239 |  | State_UT | 67.4172 | 5.1481 |
| State_VA | 22.2351 | 3.23 |  | State_VA | 68.1428 | 5.1842 |
| State_VT | 20.2395 | 3.2378 |  | State_VT | 65.8494 | 5.1845 |
| State_WA | 21.9887 | 3.2244 |  | State_WA | 67.4178 | 5.1773 |
| State_WI | 21.8518 | 3.2308 |  | State_WI | 67.4209 | 5.1734 |
| State_WV | 21.1489 | 3.2386 |  | State_WV | 66.9085 | 5.175 |
| State_WY | 19.9215 | 3.2394 |  | State_WY | 65.3417 | 5.156 |
| t0 [LogDays] | -10.0802 | 1.5057 |  | t0 [LogDays] | -29.2265 | 2.4812 |
| t2 [LogDays^2] | 1.1462 | 0.1748 |  | t2 [LogDays^2] | 3.3075 | 0.2953 |
| b1 [L7.LogDailyDead] | 0.8731 | 0.0267 |  | b1 [L14.DailyDead] | 0.8049 | 0.0528 |
| j1 [L7.MaxTempRatio] | -1.3784 | 0.2482 |  | d1 [L7.LogMaxTemp] | 0.1907 | 0.1491 |
| j2 [L14.MaxTempRatio] | -1.2842 | 0.2182 |  | d2 [L14.LogMaxTemp] | 0.4566 | 0.0667 |
|  |  |  |  |  |  |  |
| R-square | 0.9709 |  |  | R-square | 0.9473 |  |
| Root MSE | 8.5684 |  |  | Root MSE | 11.5266 |  |
| Observations | 4567 |  |  | Observations | 4567 |  |

| **Model 7** |  |  |  | **Model 8** |  |  |
| --- | --- | --- | --- | --- | --- | --- |
| Variable | Coefficient | S.E. |  | Variable | Coefficient | S.E. |
| State_AK | 32.2561 | 3.3204 |  | State_AK | 31.4104 | 4.935 |
| State_AL | 33.4098 | 3.4613 |  | State_AL | 33.7675 | 4.9922 |
| State_AR | 33.1846 | 3.4181 |  | State_AR | 34.9329 | 6.5912 |
| State_AZ | 33.5965 | 3.4931 |  | State_AZ | 34.0518 | 4.9494 |
| State_CA | 33.5544 | 3.5253 |  | State_CA | 33.9114 | 4.8834 |
| State_CO | 33.2358 | 3.4949 |  | State_CO | 33.5507 | 4.9353 |
| State_CT | 33.3913 | 3.5326 |  | State_CT | 33.6648 | 4.9135 |
| State_DC | 33.3387 | 3.4602 |  | State_DC | 33.4086 | 4.9777 |
| State_DE | 33.3536 | 3.463 |  | State_DE | 33.3454 | 4.907 |
| State_FL | 33.6267 | 3.5214 |  | State_FL | 34.1685 | 4.9652 |
| State_GA | 33.4411 | 3.5043 |  | State_GA | 33.9497 | 5.0074 |
| State_HI | 32.235 | 3.3628 |  | State_HI | 31.2108 | 4.9078 |
| State_IA | 33.4024 | 3.465 |  | State_IA | 35.421 | 6.5281 |
| State_ID | 32.6949 | 3.3931 |  | State_ID | 32.6437 | 4.9101 |
| State_IL | 33.5292 | 3.5385 |  | State_IL | 33.8479 | 4.9023 |
| State_IN | 33.3958 | 3.5026 |  | State_IN | 33.6863 | 4.9253 |
| State_KS | 33.0417 | 3.4258 |  | State_KS | 33.0641 | 4.9609 |
| State_KY | 33.2089 | 3.4448 |  | State_KY | 33.1836 | 4.9321 |
| State_LA | 33.2585 | 3.5182 |  | State_LA | 33.6462 | 4.9107 |
| State_MA | 33.4843 | 3.5513 |  | State_MA | 33.7928 | 4.9266 |
| State_MD | 33.4762 | 3.5157 |  | State_MD | 33.8419 | 4.9784 |
| State_ME | 32.8432 | 3.393 |  | State_ME | 32.4265 | 4.9926 |
| State_MI | 33.2494 | 3.5412 |  | State_MI | 33.5583 | 4.9126 |
| State_MN | 33.4384 | 3.4867 |  | State_MN | 33.6694 | 4.9433 |
| State_MO | 33.3401 | 3.4748 |  | State_MO | 33.6839 | 5.0421 |
| State_MS | 33.4338 | 3.468 |  | State_MS | 33.7608 | 4.9809 |
| State_MT | 32.7862 | 3.3443 |  | State_MT | 32.1433 | 4.9335 |
| State_NC | 33.4229 | 3.4781 |  | State_NC | 33.7394 | 4.9644 |
| State_ND | 33.0229 | 3.3904 |  | State_ND | 34.4903 | 6.5509 |
| State_NE | 33.1492 | 3.4206 |  | State_NE | 35.0003 | 6.5482 |
| State_NH | 33.275 | 3.4368 |  | State_NH | 33.2621 | 4.9404 |
| State_NJ | 33.3541 | 3.5798 |  | State_NJ | 33.7331 | 4.8955 |
| State_NM | 33.3743 | 3.4505 |  | State_NM | 33.4305 | 4.9106 |
| State_NV | 33.295 | 3.4474 |  | State_NV | 33.547 | 4.9722 |
| State_NY | 33.2705 | 3.6001 |  | State_NY | 33.658 | 4.9073 |
| State_OH | 33.4485 | 3.5053 |  | State_OH | 33.6993 | 4.9158 |
| State_OK | 33.1365 | 3.4407 |  | State_OK | 34.9644 | 6.3832 |
| State_OR | 33.0518 | 3.4061 |  | State_OR | 32.9705 | 4.8949 |
| State_PA | 33.4902 | 3.549 |  | State_PA | 33.9518 | 5.0137 |
| State_RI | 33.3732 | 3.4744 |  | State_RI | 33.5337 | 4.9486 |
| State_SC | 33.4631 | 3.4531 |  | State_SC | 33.9176 | 4.9945 |
| State_SD | 33.0889 | 3.3936 |  | State_SD | 34.7205 | 6.5824 |
| State_TN | 33.3154 | 3.4405 |  | State_TN | 33.4996 | 4.9642 |
| State_TX | 33.7662 | 3.509 |  | State_TX | 34.3117 | 4.9565 |
| State_UT | 33.254 | 3.4022 |  | State_UT | 34.9622 | 6.5357 |
| State_VA | 33.4343 | 3.5007 |  | State_VA | 33.7297 | 4.9679 |
| State_VT | 32.5205 | 3.377 |  | State_VT | 31.6356 | 4.9082 |
| State_WA | 33.1464 | 3.4667 |  | State_WA | 33.3658 | 4.8965 |
| State_WI | 33.1774 | 3.4511 |  | State_WI | 33.2796 | 4.9188 |
| State_WV | 32.9836 | 3.4022 |  | State_WV | 32.5613 | 4.9068 |
| State_WY | 32.566 | 3.3502 |  | State_WY | 33.2934 | 6.6178 |
| t0 [LogDays] | -13.9999 | 1.532 |  | t0 [LogDays] | -14.034 | 2.4107 |
| t2 [LogDays^2] | 1.5822 | 0.1787 |  | t2 [LogDays^2] | 1.5365 | 0.2261 |
| b1 [L7.LogWeeklyDead] | 0.9204 | 0.0487 |  | s1 [LogDaysShelterInPlace] | 0.4676 | 0.5186 |
| h1 [L14.LogMaxTemp] | 0.2111 | 0.0498 |  | s2 [LogDaysReopen] | -0.0436 | 0.0255 |
| h2 [L18.LogMaxTemp] | -233.139 |  |  | b1 [L7.LogDailyDead] | 0.8455 | 0.0261 |
|  |  |  |  | w1 [L7.LogMaxTemp] | 0.3091 | 0.0454 |
| R-square | 0.9872 |  |  | w2 [L14.LogMaxTemp] | 0.2828 | 0.0477 |
| Root MSE | 38.9815 |  |  |  |  |  |
| Observations | 4552 |  |  | R-square | 0.9715 |  |
|  |  |  |  | Root MSE | 8.4736 |  |
|  |  |  |  | Observations | 4567 |  |

| **Model 9** |  |  |  | **Model 10** |  |  |
| --- | --- | --- | --- | --- | --- | --- |
| Variable | Coefficient | S.E. |  | Variable | Coefficient | S.E. |
| State_AK | 1.6964 | 0.3785 |  | State_AK | 26.5266 | 3.1055 |
| State_AL | 3.7798 | 0.4773 |  | State_AL | 28.8017 | 3.0899 |
| State_AR | 2.333 | 0.2021 |  | State_AR | 28.1594 | 3.0897 |
| State_AZ | 4.196 | 0.5488 |  | State_AZ | 29.1509 | 3.0648 |
| State_CA | 4.162 | 0.5675 |  | State_CA | 29.1089 | 3.0833 |
| State_CO | 3.7124 | 0.4649 |  | State_CO | 28.6904 | 3.0921 |
| State_CT | 3.8862 | 0.5139 |  | State_CT | 28.8466 | 3.094 |
| State_DC | 3.699 | 0.4296 |  | State_DC | 28.5473 | 3.1052 |
| State_DE | 3.7718 | 0.449 |  | State_DE | 28.5738 | 3.1075 |
| State_FL | 4.2104 | 0.5806 |  | State_FL | 29.2243 | 3.0675 |
| State_GA | 3.8712 | 0.4816 |  | State_GA | 28.9659 | 3.0915 |
| State_HI | 1.6367 | 0.3755 |  | State_HI | 26.4053 | 3.1029 |
| State_IA | 2.7742 | 0.2048 |  | State_IA | 28.683 | 3.1046 |
| State_ID | 3.1006 | 0.3894 |  | State_ID | 27.8497 | 3.0952 |
| State_IL | 4.0488 | 0.5567 |  | State_IL | 29.0402 | 3.095 |
| State_IN | 3.8451 | 0.4922 |  | State_IN | 28.8334 | 3.0985 |
| State_KS | 3.3612 | 0.408 |  | State_KS | 28.1942 | 3.0974 |
| State_KY | 3.4656 | 0.4569 |  | State_KY | 28.3432 | 3.0984 |
| State_LA | 3.901 | 0.4929 |  | State_LA | 28.8356 | 3.0905 |
| State_MA | 3.9241 | 0.5407 |  | State_MA | 28.9445 | 3.094 |
| State_MD | 3.9222 | 0.4966 |  | State_MD | 28.9345 | 3.098 |
| State_ME | 2.6877 | 0.3754 |  | State_ME | 27.5148 | 3.1046 |
| State_MI | 3.7847 | 0.5199 |  | State_MI | 28.7407 | 3.0878 |
| State_MN | 3.8679 | 0.4876 |  | State_MN | 28.8101 | 3.1019 |
| State_MO | 3.6926 | 0.4345 |  | State_MO | 28.6925 | 3.0982 |
| State_MS | 3.823 | 0.473 |  | State_MS | 28.8192 | 3.092 |
| State_MT | 2.4313 | 0.3739 |  | State_MT | 27.2822 | 3.1018 |
| State_NC | 3.872 | 0.481 |  | State_NC | 28.8363 | 3.0941 |
| State_ND | 1.9901 | 0.1592 |  | State_ND | 27.7409 | 3.1054 |
| State_NE | 2.4107 | 0.1918 |  | State_NE | 28.2476 | 3.0989 |
| State_NH | 3.5587 | 0.44 |  | State_NH | 28.4099 | 3.1039 |
| State_NJ | 3.8833 | 0.5696 |  | State_NJ | 28.9229 | 3.0845 |
| State_NM | 3.7611 | 0.4591 |  | State_NM | 28.6264 | 3.0939 |
| State_NV | 3.7639 | 0.4342 |  | State_NV | 28.6598 | 3.0854 |
| State_NY | 3.7346 | 0.5904 |  | State_NY | 28.8179 | 3.0806 |
| State_OH | 3.8967 | 0.5055 |  | State_OH | 28.8641 | 3.0987 |
| State_OK | 2.6241 | 0.1861 |  | State_OK | 28.4057 | 3.0994 |
| State_OR | 3.4807 | 0.4246 |  | State_OR | 28.1981 | 3.0956 |
| State_PA | 3.9123 | 0.5154 |  | State_PA | 28.9994 | 3.0937 |
| State_RI | 3.7388 | 0.4562 |  | State_RI | 28.6674 | 3.1052 |
| State_SC | 3.9502 | 0.4648 |  | State_SC | 28.9358 | 3.0844 |
| State_SD | 2.1792 | 0.167 |  | State_SD | 27.9572 | 3.1011 |
| State_TN | 3.6488 | 0.4671 |  | State_TN | 28.5801 | 3.0876 |
| State_TX | 4.381 | 0.5738 |  | State_TX | 29.3765 | 3.0669 |
| State_UT | 2.5463 | 0.1778 |  | State_UT | 28.2886 | 3.0892 |
| State_VA | 3.8788 | 0.4774 |  | State_VA | 28.8466 | 3.1002 |
| State_VT | 2.1979 | 0.3658 |  | State_VT | 26.8544 | 3.1042 |
| State_WA | 3.7884 | 0.4561 |  | State_WA | 28.5893 | 3.0939 |
| State_WI | 3.6155 | 0.4624 |  | State_WI | 28.4575 | 3.0997 |
| State_WV | 2.9435 | 0.4048 |  | State_WV | 27.7501 | 3.1072 |
| State_WY | 0.8501 | 0.1374 |  | State_WY | 26.5103 | 3.1044 |
| s1 [LogDaysShelterInPlace] | -0.2255 | 0.0943 |  | t0 [LogDays] | -10.3784 | 1.4517 |
| s2 [LogDaysReopen] | 0.0742 | 0.0159 |  | t2 [LogDays^2] | 1.1834 | 0.1686 |
| b1 [L7.LogDailyDead] | 0.9567 | 0.0497 |  | b1 [L7.LogDailyDead] | 0.8671 | 0.0276 |
| w1 [LogTemp1] | 0.2838 | 0.0494 |  | w1 [L7.LogMaxTemp] | 0.3171 | 0.0629 |
| w2 [LogTemp2] | 0.2082 | 0.075 |  | w2 [L14.LogMaxTemp] | 0.2985 | 0.0678 |
|  |  |  |  | a1 [L7.LogMaxAbsoluteHumidity] | -0.0115 | 0.0467 |
| R-square | 0.9667 |  |  | a2 [L14.LogMaxAbsoluteHumidity] | -0.0073 | 0.0449 |
| Root MSE | 9.1598 |  |  |  |  |  |
| Observations | 4567 |  |  | R-square | 0.9711 |  |
|  |  |  |  | Root MSE | 8.5446 |  |
|  |  |  |  | Observations | 4567 |  |

| **Model 11** |  |  |  | **Model 12** |  |  |
| --- | --- | --- | --- | --- | --- | --- |
| Variable | Coefficient | S.E. |  | Variable | Coefficient | S.E. |
| State_AK | 26.6169 | 3.2169 |  | State_AK | 23.3848 | 2.5747 |
| State_AL | 28.8918 | 3.2007 |  | State_AL | 25.9957 | 2.5950 |
| State_AR | 28.2515 | 3.2008 |  | State_AR | 25.3367 | 2.5905 |
| State_AZ | 29.2055 | 3.1650 |  | State_AZ | 26.3314 | 2.5762 |
| State_CA | 29.1995 | 3.1939 |  | State_CA | 26.3196 | 2.5921 |
| State_CO | 28.7754 | 3.2051 |  | State_CO | 25.8690 | 2.6028 |
| State_CT | 28.9373 | 3.2058 |  | State_CT | 25.9763 | 2.5951 |
| State_DC | 28.6365 | 3.2151 |  | State_DC | 25.6883 | 2.6041 |
| State_DE | 28.6637 | 3.2183 |  | State_DE | 25.7101 | 2.6057 |
| State_FL | 29.3147 | 3.1774 |  | State_FL | 26.4667 | 2.5793 |
| State_GA | 29.0541 | 3.2016 |  | State_GA | 26.1701 | 2.6009 |
| State_HI | 26.4927 | 3.2109 |  | State_HI | 23.6253 | 2.6109 |
| State_IA | 28.7743 | 3.2156 |  | State_IA | 25.8237 | 2.6019 |
| State_ID | 27.9337 | 3.2059 |  | State_ID | 24.9495 | 2.5918 |
| State_IL | 29.1290 | 3.2062 |  | State_IL | 26.1816 | 2.5966 |
| State_IN | 28.9249 | 3.2097 |  | State_IN | 25.9827 | 2.5999 |
| State_KS | 28.2824 | 3.2076 |  | State_KS | 25.3504 | 2.5996 |
| State_KY | 28.4345 | 3.2093 |  | State_KY | 25.4941 | 2.5974 |
| State_LA | 28.9256 | 3.1989 |  | State_LA | 26.0839 | 2.6071 |
| State_MA | 29.0352 | 3.2046 |  | State_MA | 26.0833 | 2.5958 |
| State_MD | 29.0235 | 3.2089 |  | State_MD | 26.0834 | 2.5999 |
| State_ME | 27.6052 | 3.2150 |  | State_ME | 24.6205 | 2.5975 |
| State_MI | 28.8309 | 3.1990 |  | State_MI | 25.8658 | 2.5891 |
| State_MN | 28.8969 | 3.2127 |  | State_MN | 25.9199 | 2.5979 |
| State_MO | 28.7805 | 3.2081 |  | State_MO | 25.8363 | 2.5992 |
| State_MS | 28.9108 | 3.2030 |  | State_MS | 26.0198 | 2.5979 |
| State_MT | 27.3655 | 3.2127 |  | State_MT | 24.3663 | 2.5955 |
| State_NC | 28.9269 | 3.2050 |  | State_NC | 26.0204 | 2.5983 |
| State_ND | 27.8313 | 3.2170 |  | State_ND | 24.8248 | 2.5950 |
| State_NE | 28.3377 | 3.2106 |  | State_NE | 25.3831 | 2.5958 |
| State_NH | 28.4981 | 3.2152 |  | State_NH | 25.5112 | 2.5981 |
| State_NJ | 29.0115 | 3.1945 |  | State_NJ | 26.0706 | 2.5901 |
| State_NM | 28.6891 | 3.2009 |  | State_NM | 25.8209 | 2.6080 |
| State_NV | 28.7147 | 3.1847 |  | State_NV | 25.8182 | 2.5947 |
| State_NY | 28.9107 | 3.1907 |  | State_NY | 25.9800 | 2.5874 |
| State_OH | 28.9557 | 3.2099 |  | State_OH | 26.0046 | 2.5983 |
| State_OK | 28.4974 | 3.2102 |  | State_OK | 25.5851 | 2.6053 |
| State_OR | 28.2874 | 3.2067 |  | State_OR | 25.2682 | 2.5853 |
| State_PA | 29.0869 | 3.2041 |  | State_PA | 26.1480 | 2.5974 |
| State_RI | 28.7591 | 3.2167 |  | State_RI | 25.7913 | 2.6021 |
| State_SC | 29.0278 | 3.1954 |  | State_SC | 26.1274 | 2.5879 |
| State_SD | 28.0474 | 3.2123 |  | State_SD | 25.0731 | 2.5937 |
| State_TN | 28.6700 | 3.1985 |  | State_TN | 25.7575 | 2.5899 |
| State_TX | 29.4680 | 3.1784 |  | State_TX | 26.5874 | 2.5751 |
| State_UT | 28.3670 | 3.1973 |  | State_UT | 25.4439 | 2.5926 |
| State_VA | 28.9362 | 3.2112 |  | State_VA | 25.9958 | 2.6018 |
| State_VT | 26.9456 | 3.2142 |  | State_VT | 23.9513 | 2.5951 |
| State_WA | 28.6790 | 3.2050 |  | State_WA | 25.6481 | 2.5848 |
| State_WI | 28.5475 | 3.2100 |  | State_WI | 25.5814 | 2.5961 |
| State_WV | 27.8425 | 3.2184 |  | State_WV | 24.8947 | 2.6049 |
| State_WY | 26.5997 | 3.2182 |  | State_WY | 23.6784 | 2.6046 |
| t0 [LogDays] | -10.3649 | 1.4688 |  | t0 [LogDays] | -9.0650 | 1.2291 |
| t2 [LogDays^2] | 1.1819 | 0.1707 |  | t2 [LogDays^2] | 1.0460 | 0.1461 |
| b1 [L7.LogDailyDead] | 0.8671 | 0.0278 |  | b1 [L7.LogDailyDead] | 0.8585 | 0.0280 |
| w1 [L7.LogMaxTemp] | 0.3066 | 0.0509 |  | w1 [LogMaxTemp] | 0.2010 | 0.0981 |
| w2 [L14.LogMaxTemp] | 0.2923 | 0.0555 |  | w2 [LogTemp2] | 0.2540 | 0.0568 |
| a1 [L7.LogMaxRelativeHumidity] | 0.0327 | 0.1632 |  | a1 [L7.LogUV] | 0.4099 | 0.1729 |
| a2 [L14.LogMaxRelativeHumidity] | 0.0789 | 0.1369 |  | a2 [L14.LogUV] | 0.2463 | 0.1256 |
|  |  |  |  |  |  |  |
| R-square | 0.9711 |  |  | R-square | 0.9714 |  |
| Root MSE | 8.5435 |  |  | Root MSE | 8.4871 |  |
| Observations | 4567 |  |  | Observations | 4567 |  |

| **Model 13** |  |  |  | **Model 14** |  |  |
| --- | --- | --- | --- | --- | --- | --- |
| Variable | Coefficient | S.E. |  | Variable | Coefficient | S.E. |
| State_AK | 16.969 | 3.4688 |  | State_AK | 13.0080 | 9.4448 |
| State_AL | 19.7433 | 3.4785 |  | State_AL | 15.5707 | 9.1048 |
| State_AR | 19.0774 | 3.4767 |  | State_AR | 14.8156 | 9.2406 |
| State_AZ | 20.0468 | 3.456 |  | State_AZ | 15.9942 | 9.0411 |
| State_CA | 20.0842 | 3.4681 |  | State_CA | 15.9084 | 9.1322 |
| State_CO | 19.627 | 3.4825 |  | State_CO | 15.6307 | 8.9448 |
| State_CT | 19.7071 | 3.47 |  | State_CT | 16.0504 | 8.7631 |
| State_DC | 19.4141 | 3.4901 |  | State_DC | 15.5470 | 8.8720 |
| State_DE | 19.4443 | 3.4912 |  | State_DE | 15.5108 | 8.9217 |
| State_FL | 20.2337 | 3.4527 |  | State_FL | 16.0985 | 9.0458 |
| State_GA | 19.9196 | 3.4821 |  | State_GA | 15.8475 | 9.0068 |
| State_HI | 17.4122 | 3.5025 |  | State_HI | 12.8610 | 9.5273 |
| State_IA | 19.5496 | 3.4877 |  | State_IA | 15.4477 | 9.1109 |
| State_ID | 18.666 | 3.4804 |  | State_ID | 14.5347 | 9.2003 |
| State_IL | 19.9 | 3.4734 |  | State_IL | 15.9992 | 8.9711 |
| State_IN | 19.7164 | 3.4803 |  | State_IN | 15.7524 | 8.9707 |
| State_KS | 19.0856 | 3.487 |  | State_KS | 14.9309 | 9.1410 |
| State_KY | 19.2339 | 3.4813 |  | State_KY | 15.0905 | 9.1460 |
| State_LA | 19.8514 | 3.4877 |  | State_LA | 15.9398 | 8.8111 |
| State_MA | 19.8296 | 3.468 |  | State_MA | 16.1128 | 8.8109 |
| State_MD | 19.8026 | 3.4805 |  | State_MD | 15.8827 | 8.9550 |
| State_ME | 18.3692 | 3.4832 |  | State_ME | 14.2255 | 9.1741 |
| State_MI | 19.5863 | 3.4614 |  | State_MI | 15.8606 | 8.8240 |
| State_MN | 19.6244 | 3.4821 |  | State_MN | 15.6084 | 9.0795 |
| State_MO | 19.5555 | 3.4836 |  | State_MO | 15.4978 | 9.0844 |
| State_MS | 19.7711 | 3.4825 |  | State_MS | 15.6846 | 8.9946 |
| State_MT | 18.069 | 3.4866 |  | State_MT | 13.7737 | 9.4442 |
| State_NC | 19.7613 | 3.482 |  | State_NC | 15.5685 | 9.1750 |
| State_ND | 18.5309 | 3.4859 |  | State_ND | 14.4049 | 9.1886 |
| State_NE | 19.101 | 3.4837 |  | State_NE | 14.9446 | 9.1772 |
| State_NH | 19.2279 | 3.4851 |  | State_NH | 15.2093 | 9.0472 |
| State_NJ | 19.7885 | 3.4598 |  | State_NJ | 16.3273 | 8.6619 |
| State_NM | 19.5795 | 3.4962 |  | State_NM | 15.3835 | 9.0973 |
| State_NV | 19.5342 | 3.4821 |  | State_NV | 15.4590 | 9.0525 |
| State_NY | 19.7127 | 3.4524 |  | State_NY | 16.1767 | 8.6987 |
| State_OH | 19.7397 | 3.4775 |  | State_OH | 15.7123 | 9.0548 |
| State_OK | 19.3444 | 3.4909 |  | State_OK | 15.1625 | 9.1357 |
| State_OR | 18.9496 | 3.474 |  | State_OR | 14.8419 | 9.2576 |
| State_PA | 19.8696 | 3.4729 |  | State_PA | 16.0094 | 8.9240 |
| State_RI | 19.5293 | 3.4844 |  | State_RI | 15.7015 | 8.8582 |
| State_SC | 19.8761 | 3.4709 |  | State_SC | 15.6887 | 9.1117 |
| State_SD | 18.788 | 3.4841 |  | State_SD | 14.6261 | 9.1816 |
| State_TN | 19.4982 | 3.4739 |  | State_TN | 15.2758 | 9.2160 |
| State_TX | 20.3344 | 3.4498 |  | State_TX | 16.1276 | 9.1790 |
| State_UT | 19.1961 | 3.4797 |  | State_UT | 14.8950 | 9.3015 |
| State_VA | 19.721 | 3.4835 |  | State_VA | 15.6862 | 9.0582 |
| State_VT | 17.7011 | 3.4817 |  | State_VT | 13.5623 | 9.0439 |
| State_WA | 19.3149 | 3.4685 |  | State_WA | 15.4422 | 9.0164 |
| State_WI | 19.3263 | 3.4778 |  | State_WI | 15.2179 | 9.1390 |
| State_WV | 18.6385 | 3.4938 |  | State_WV | 14.3606 | 9.3349 |
| State_WY | 17.4851 | 3.4926 |  | State_WY | 13.0400 | 9.3944 |
| t0 [LogDays] | -7.519 | 1.6319 |  | t0 [LogDays] | -5.8845 | 3.7750 |
| t2 [LogDays^2] | 0.8758 | 0.1917 |  | t2 [LogDays^2] | 0.7115 | 0.3968 |
| b1 [L7.LogDailyDead] | 0.8625 | 0.0286 |  | c1 [L7.LogTotalDead] | -0.4484 | 0.2737 |
| a1 [L7.LogUV] | 0.5808 | 0.0818 |  | c2 [L.7LogTotalDead^2] | 0.0153 | 0.0332 |
| a2 [L14.LogUV] | 0.4513 | 0.0703 |  | b1 [L7.LogDailyDead] | 0.8486 | 0.0263 |
|  |  |  |  | w1 [L7.LogMaxTemp] | 0.3072 | 0.0439 |
| R-square | 0.9707 |  |  | w2 [L14.LogMaxTemp] | 0.3008 | 0.0445 |
| Root MSE | 8.589 |  |  |  |  |  |
| Observations | 4567 |  |  | R-square | 0.9720 |  |
|  |  |  |  | RMSE | 8.4007 |  |
|  |  |  |  | Observations | 4567 |  |
| **Model 15** |  |  |  | **Model 16** |  |  |
| Variable | Coefficient | S.E. |  | Variable | Coefficient | S.E. |
| State_AK | 7.9889 | 8.4007 |  | State_AK | 16.3166 | 4.4703 |
| State_AL | 10.2548 | 8.1560 |  | State_AL | 18.6537 | 4.4968 |
| State_AR | 9.4507 | 8.2510 |  | State_AR | 17.6139 | 4.4878 |
| State_AZ | 10.7131 | 8.1163 |  | State_AZ | 19.6201 | 4.4545 |
| State_CA | 10.5192 | 8.1838 |  | State_CA | 19.9896 | 4.447 |
| State_CO | 10.4215 | 8.0175 |  | State_CO | 19.2243 | 4.5283 |
| State_CT | 10.9961 | 7.8718 |  | State_CT | 19.4508 | 4.507 |
| State_DC | 10.4747 | 7.9604 |  | State_DC | 18.1874 | 4.5401 |
| State_DE | 10.4000 | 8.0016 |  | State_DE | 18.2435 | 4.5343 |
| State_FL | 10.7744 | 8.1230 |  | State_FL | 20.0075 | 4.4341 |
| State_GA | 10.5814 | 8.0761 |  | State_GA | 19.3797 | 4.482 |
| State_HI | 7.9147 | 8.4609 |  | State_HI | 16.7115 | 4.5421 |
| State_IA | 10.1815 | 8.1528 |  | State_IA | 18.4268 | 4.5156 |
| State_ID | 9.2760 | 8.2214 |  | State_ID | 17.3865 | 4.5149 |
| State_IL | 10.7600 | 8.0522 |  | State_IL | 19.8017 | 4.4941 |
| State_IN | 10.5332 | 8.0432 |  | State_IN | 19.4925 | 4.5182 |
| State_KS | 9.6364 | 8.1730 |  | State_KS | 17.785 | 4.5217 |
| State_KY | 9.7601 | 8.1802 |  | State_KY | 18.438 | 4.5187 |
| State_LA | 10.8675 | 7.9156 |  | State_LA | 18.5493 | 4.4693 |
| State_MA | 10.9996 | 7.9145 |  | State_MA | 19.5946 | 4.4873 |
| State_MD | 10.6815 | 8.0346 |  | State_MD | 19.2539 | 4.5057 |
| State_ME | 8.9362 | 8.1923 |  | State_ME | 17.7007 | 4.5445 |
| State_MI | 10.7409 | 7.9237 |  | State_MI | 19.9065 | 4.5004 |
| State_MN | 10.3287 | 8.1326 |  | State_MN | 19.0708 | 4.5029 |
| State_MO | 10.2049 | 8.1314 |  | State_MO | 18.9333 | 4.4889 |
| State_MS | 10.4798 | 8.0690 |  | State_MS | 18.6953 | 4.4983 |
| State_MT | 8.7047 | 8.4003 |  | State_MT | 17.598 | 4.4906 |
| State_NC | 10.2238 | 8.2032 |  | State_NC | 19.0158 | 4.4756 |
| State_ND | 9.1377 | 8.2022 |  | State_ND | 16.896 | 4.5264 |
| State_NE | 9.6376 | 8.1981 |  | State_NE | 17.6159 | 4.5258 |
| State_NH | 10.0030 | 8.1024 |  | State_NH | 18.4673 | 4.5476 |
| State_NJ | 11.2725 | 7.7877 |  | State_NJ | 19.8937 | 4.4926 |
| State_NM | 10.1470 | 8.1389 |  | State_NM | 18.2939 | 4.5044 |
| State_NV | 10.1908 | 8.1085 |  | State_NV | 18.3879 | 4.4819 |
| State_NY | 11.0965 | 7.8236 |  | State_NY | 19.7594 | 4.4618 |
| State_OH | 10.4155 | 8.1122 |  | State_OH | 19.6928 | 4.5082 |
| State_OK | 9.8572 | 8.1683 |  | State_OK | 17.5467 | 4.5171 |
| State_OR | 9.5405 | 8.2662 |  | State_OR | 17.9365 | 4.5116 |
| State_PA | 10.7952 | 8.0097 |  | State_PA | 20.0858 | 4.5052 |
| State_RI | 10.6304 | 7.9481 |  | State_RI | 18.1985 | 4.5154 |
| State_SC | 10.3711 | 8.1616 |  | State_SC | 18.9101 | 4.4698 |
| State_SD | 9.3521 | 8.1945 |  | State_SD | 16.9805 | 4.5396 |
| State_TN | 9.9225 | 8.2399 |  | State_TN | 18.0694 | 4.4724 |
| State_TX | 10.6993 | 8.2230 |  | State_TX | 20.1334 | 4.4528 |
| State_UT | 9.6144 | 8.2824 |  | State_UT | 17.2871 | 4.4865 |
| State_VA | 10.3939 | 8.1130 |  | State_VA | 19.0325 | 4.503 |
| State_VT | 8.3048 | 8.0904 |  | State_VT | 16.9392 | 4.5418 |
| State_WA | 10.1814 | 8.0777 |  | State_WA | 18.5552 | 4.4964 |
| State_WI | 9.9084 | 8.1748 |  | State_WI | 18.4741 | 4.4965 |
| State_WV | 8.9806 | 8.2816 |  | State_WV | 17.5575 | 4.516 |
| State_WY | 7.7949 | 8.3436 |  | State_WY | 15.7526 | 4.545 |
| t0 [LogDays] | -4.1101 | 3.2902 |  | t0 [LogDays] | -6.407 | 2.2067 |
| t2 [LogDays^2] | 0.5338 | 0.3469 |  | t2 [LogDays^2] | 0.4771 | 0.2705 |
| c1 [L7.LogTotalDead] | -0.4186 | 0.2449 |  | n1 [L7.LogNewTests] | -0.2831 | 0.082 |
| c2 [L7.LogTotalDead^2] | -0.0408 | 0.1230 |  | pc1 [L7.LogPerCapitaTests] | 0.6806 | 0.1918 |
| c3 [L7.LogTotalDead^3] | -0.0178 | 0.0522 |  | bp [L7.LogNewPositives] | 0.7149 | 0.0511 |
| c4 [L7.LogTotalDead^4] | -0.0049 | 0.0072 |  | w1 [L7.LogMaxTemp] | 0.2564 | 0.0436 |
| b1 [L7.LogDailyDead] | 0.8985 | 0.0318 |  |  |  |  |
| w1 [L7.LogMaxTemp] | 0.2967 | 0.0548 |  | R-square | 0.9494 |  |
| w2 [L14.LogMaxTemp] | 0.2812 | 0.0362 |  | Root MSE | 11.301 |  |
|  |  |  |  | Observations | 4567 |  |
| R-square | 0.9734 |  |  |  |  |  |
| RMSE | 8.1932 |  |  |  |  |  |
| Observations | 4567 |  |  |  |  |  |

| **Model 17** |  |  |  | **Model 18** |  |  |
| --- | --- | --- | --- | --- | --- | --- |
| Variable | Coefficient | S.E. |  | Variable | Coefficient | S.E. |
| State_AK | 44.3754 | 7.822 |  | L7.ReportedDailyDead | 0.6986 | 0.0069 |
| State_AL | 45.0719 | 7.7439 |  | _cons | 5.0046 | 0.421 |
| State_AR | 45.0296 | 7.7613 |  |  |  |  |
| State_AZ | 45.4897 | 7.7481 |  | R-square | 0.6893 |  |
| State_CA | 45.2471 | 7.8135 |  | Root MSE | 26.5453 |  |
| State_CO | 44.7237 | 7.7036 |  | Observations | 4639 |  |
| State_CT | 44.971 | 7.8123 |  |  |  |  |
| State_DC | 44.848 | 7.7886 |  |  |  |  |
| State_DE | 44.8834 | 7.7766 |  | **Model 19** |  |  |
| State_FL | 45.7236 | 7.8081 |  | Variable | Coefficient | S.E. |
| State_GA | 45.2116 | 7.7504 |  | L7.ReportedDailyDead | 0.6663 | 0.0063 |
| State_HI | 43.536 | 7.5748 |  | _cons | 6.4614 | 0.3873 |
| State_IA | 44.9209 | 7.7604 |  |  |  |  |
| State_ID | 44.4862 | 7.6268 |  | R-square | 0.7046 |  |
| State_IL | 45.2043 | 7.832 |  | Root MSE | 24.4192 |  |
| State_IN | 44.7918 | 7.7378 |  | Observations | 4639 |  |
| State_KS | 44.8676 | 7.6937 |  |  |  |  |
| State_KY | 44.4355 | 7.7336 |  |  |  |  |
| State_LA | 46.0003 | 7.9379 |  | **Model 20** |  |  |
| State_MA | 45.0429 | 7.8516 |  | Variable | Coefficient | S.E. |
| State_MD | 45.1639 | 7.8065 |  | L7.DailyDead | 0.7434 | 0.0063 |
| State_ME | 43.7347 | 7.6623 |  | _cons | 3.3406 | 0.382 |
| State_MI | 44.6835 | 7.7889 |  |  |  |  |
| State_MN | 44.8802 | 7.7795 |  | R-square | 0.7485 |  |
| State_MO | 44.942 | 7.7386 |  | Root MSE | 23.8845 |  |
| State_MS | 45.1696 | 7.7728 |  | Observations | 4639 |  |
| State_MT | 44.2548 | 7.649 |  |  |  |  |
| State_NC | 45.0362 | 7.7549 |  |  |  |  |
| State_ND | 44.4879 | 7.7784 |  | **Model 21** |  |  |
| State_NE | 44.8758 | 7.759 |  | Variable | Coefficient | S.E. |
| State_NH | 44.2717 | 7.7343 |  | L7.DailyDead | 0.7798 | 0.0033 |
| State_NJ | 45.2459 | 7.8555 |  | _cons | 3.1815 | 0.1965 |
| State_NM | 45.0833 | 7.8327 |  |  |  |  |
| State_NV | 45.1552 | 7.7471 |  | R-square | 0.9252 |  |
| State_NY | 45.2907 | 7.8897 |  | Root MSE | 12.2862 |  |
| State_OH | 44.6637 | 7.7159 |  | Observations | 4639 |  |
| State_OK | 44.7409 | 7.7293 |  |  |  |  |
| State_OR | 43.9776 | 7.6495 |  |  |  |  |
| State_PA | 44.8583 | 7.7437 |  |  |  |  |
| State_RI | 44.848 | 7.8233 |  |  |  |  |
| State_SC | 45.0818 | 7.7231 |  |  |  |  |
| State_SD | 44.4404 | 7.7294 |  |  |  |  |
| State_TN | 45.3233 | 7.8145 |  |  |  |  |
| State_TX | 45.3508 | 7.7418 |  |  |  |  |
| State_UT | 44.9916 | 7.7777 |  |  |  |  |
| State_VA | 45.0741 | 7.7745 |  |  |  |  |
| State_VT | 43.1094 | 7.6813 |  |  |  |  |
| State_WA | 44.5409 | 7.7176 |  |  |  |  |
| State_WI | 44.7343 | 7.754 |  |  |  |  |
| State_WV | 44.1364 | 7.709 |  |  |  |  |
| State_WY | 43.719 | 7.6626 |  |  |  |  |
| nt0 [LogNewTests] | 0.3855 | 0.0833 |  |  |  |  |
| mt [Monday] | -0.0801 | 0.0329 |  |  |  |  |
| t1 [LogDays] | -19.5476 | 3.7152 |  |  |  |  |
| t2 [LogDays^2] | 2.3839 | 0.4551 |  |  |  |  |
| nt7 [L7.LogNewTests] | -0.1655 | 0.0584 |  |  |  |  |
| b1 [L7.LogNewPositives] | 0.5856 | 0.0469 |  |  |  |  |
| pc1 [L7.PerCapitaTests] | -0.0096 | 0.0031 |  |  |  |  |
| w1 [L2.LogmaxTemp] | 0.5435 | 0.1101 |  |  |  |  |
| w2 [L7.LogMaxTemp] | 0.6873 | 0.0460 |  |  |  |  |
|  |  |  |  |  |  |  |
| R-square | 0.9507 |  |  |  |  |  |
| Root MSE | 303.503 |  |  |  |  |  |
| Observations | 4592 |  |  |  |  |  |

| **Model 22** |  |  |  | **Model 23** |  |  |
| --- | --- | --- | --- | --- | --- | --- |
| Variable | Coefficient |  |  | Variable | Coefficient | S.E. |
| State_AK | 0.1584 |  |  | LogPopulation | 0.3763 | 0.0665 |
| State_AL | 1.5428 |  |  | PercentBlack | 0.1208 | 0.0531 |
| State_AR | 0.8118 |  |  | PercentHispanic | 0.1621 | 0.0483 |
| State_AZ | 2.1743 |  |  | _cons | -2.3671 | 0.4478 |
| State_CA | 2.0985 |  |  |  |  |  |
| State_CO | 1.3752 |  |  | R-square | 0.6649 |  |
| State_CT | 1.6126 |  |  | Root MSE | 0.3949 |  |
| State_DC | 1.1958 |  |  | Observations | 51 |  |
| State_DE | 1.2278 |  |  |  |  |  |
| State_FL | 2.3569 |  |  |  |  |  |
| State_GA | 1.8174 |  |  |  |  |  |
| State_HI | 0.1405 |  |  |  |  |  |
| State_IA | 1.3702 |  |  |  |  |  |
| State_ID | 0.5933 |  |  |  |  |  |
| State_IL | 1.9568 |  |  |  |  |  |
| State_IN | 1.5924 |  |  |  |  |  |
| State_KS | 0.8393 |  |  |  |  |  |
| State_KY | 0.9752 |  |  |  |  |  |
| State_LA | 1.5976 |  |  |  |  |  |
| State_MA | 1.7793 |  |  |  |  |  |
| State_MD | 1.7611 |  |  |  |  |  |
| State_ME | 0.4258 |  |  |  |  |  |
| State_MI | 1.4504 |  |  |  |  |  |
| State_MN | 1.5538 |  |  |  |  |  |
| State_MO | 1.3823 |  |  |  |  |  |
| State_MS | 1.5707 |  |  |  |  |  |
| State_MT | 0.3364 |  |  |  |  |  |
| State_NC | 1.5973 |  |  |  |  |  |
| State_ND | 0.5336 |  |  |  |  |  |
| State_NE | 0.8859 |  |  |  |  |  |
| State_NH | 1.0413 |  |  |  |  |  |
| State_NJ | 1.7409 |  |  |  |  |  |
| State_NM | 1.2851 |  |  |  |  |  |
| State_NV | 1.3291 |  |  |  |  |  |
| State_NY | 1.5696 |  |  |  |  |  |
| State_OH | 1.6420 |  |  |  |  |  |
| State_OK | 1.0381 |  |  |  |  |  |
| State_OR | 0.8428 |  |  |  |  |  |
| State_PA | 1.8788 |  |  |  |  |  |
| State_RI | 1.3487 |  |  |  |  |  |
| State_SC | 1.7655 |  |  |  |  |  |
| State_SD | 0.6810 |  |  |  |  |  |
| State_TN | 1.2358 |  |  |  |  |  |
| State_TX | 2.7429 |  |  |  |  |  |
| State_UT | 0.9198 |  |  |  |  |  |
| State_VA | 1.6127 |  |  |  |  |  |
| State_VT | 0.2200 |  |  |  |  |  |
| State_WA | 3.3883 |  |  |  |  |  |
| State_WI | 1.0930 |  |  |  |  |  |
| State_WV | 0.5390 |  |  |  |  |  |
| State_WY | 0.1556 |  |  |  |  |  |
|  |  |  |  |  |  |  |
|  |  |  |  |  |  |  |
|  |  |  |  |  |  |  |
|  |  |  |  |  |  |  |
|  |  |  |  |  |  |  |
|  |  |  |  |  |  |  |
|  |  |  |  |  |  |  |
|  |  |  |  |  |  |  |
|  |  |  |  |  |  |  |

| **Model 24** |  |  |  | **Model 25** |  |  |
| --- | --- | --- | --- | --- | --- | --- |
| Variable | Coefficient |  |  | Variable | Coefficient |  |
| State_AK | 24.3633 |  |  | State_AK | 0.1305 |  |
| State_AL | 237.3606 |  |  | State_AL | 1.2711 |  |
| State_AR | 124.896 |  |  | State_AR | 0.6688 |  |
| State_AZ | 334.5145 |  |  | State_AZ | 1.7914 |  |
| State_CA | 322.8477 |  |  | State_CA | 1.7289 |  |
| State_CO | 211.5753 |  |  | State_CO | 1.1330 |  |
| State_CT | 248.0875 |  |  | State_CT | 1.3285 |  |
| State_DC | 183.9717 |  |  | State_DC | 0.9852 |  |
| State_DE | 188.8931 |  |  | State_DE | 1.0115 |  |
| State_FL | 362.593 |  |  | State_FL | 1.9417 |  |
| State_GA | 279.6056 |  |  | State_GA | 1.4973 |  |
| State_HI | 21.6148 |  |  | State_HI | 0.1158 |  |
| State_IA | 210.7938 |  |  | State_IA | 1.1288 |  |
| State_ID | 91.2754 |  |  | State_ID | 0.4888 |  |
| State_IL | 301.0511 |  |  | State_IL | 1.6122 |  |
| State_IN | 244.9812 |  |  | State_IN | 1.3119 |  |
| State_KS | 129.1251 |  |  | State_KS | 0.6915 |  |
| State_KY | 150.0368 |  |  | State_KY | 0.8035 |  |
| State_LA | 245.7909 |  |  | State_LA | 1.3162 |  |
| State_MA | 273.7408 |  |  | State_MA | 1.4659 |  |
| State_MD | 270.9353 |  |  | State_MD | 1.4509 |  |
| State_ME | 65.5151 |  |  | State_ME | 0.3508 |  |
| State_MI | 223.1358 |  |  | State_MI | 1.1949 |  |
| State_MN | 239.0522 |  |  | State_MN | 1.2802 |  |
| State_MO | 212.6571 |  |  | State_MO | 1.1388 |  |
| State_MS | 241.6478 |  |  | State_MS | 1.2941 |  |
| State_MT | 51.758 |  |  | State_MT | 0.2772 |  |
| State_NC | 245.7417 |  |  | State_NC | 1.3160 |  |
| State_ND | 82.0954 |  |  | State_ND | 0.4396 |  |
| State_NE | 136.2894 |  |  | State_NE | 0.7298 |  |
| State_NH | 160.1932 |  |  | State_NH | 0.8579 |  |
| State_NJ | 267.8376 |  |  | State_NJ | 1.4343 |  |
| State_NM | 197.7059 |  |  | State_NM | 1.0587 |  |
| State_NV | 204.4823 |  |  | State_NV | 1.0950 |  |
| State_NY | 241.4787 |  |  | State_NY | 1.2932 |  |
| State_OH | 252.6185 |  |  | State_OH | 1.3528 |  |
| State_OK | 159.7133 |  |  | State_OK | 0.8553 |  |
| State_OR | 129.6555 |  |  | State_OR | 0.6943 |  |
| State_PA | 289.0446 |  |  | State_PA | 1.5479 |  |
| State_RI | 207.4894 |  |  | State_RI | 1.1111 |  |
| State_SC | 271.6136 |  |  | State_SC | 1.4545 |  |
| State_SD | 104.7713 |  |  | State_SD | 0.5611 |  |
| State_TN | 190.125 |  |  | State_TN | 1.0181 |  |
| State_TX | 421.9893 |  |  | State_TX | 2.2598 |  |
| State_UT | 141.5114 |  |  | State_UT | 0.7578 |  |
| State_VA | 248.1121 |  |  | State_VA | 1.3287 |  |
| State_VT | 33.8447 |  |  | State_VT | 0.1812 |  |
| State_WA | 521.2761 |  |  | State_WA | 2.7915 |  |
| State_WI | 168.1541 |  |  | State_WI | 0.9005 |  |
| State_WV | 82.9287 |  |  | State_WV | 0.4441 |  |
| State_WY | 23.936 |  |  | State_WY | 0.1282 |  |

| **Model 26** |  |  |  | **Model 27** |  |  |
| --- | --- | --- | --- | --- | --- | --- |
| Variable | Coefficient | S.E. |  | Variable | Coefficient | S.E. |
| State_AK | 15.6790 | 6.4956 |  | State_AK | 23.1846 | 3.1259 |
| State_AL | 19.7249 | 6.4204 |  | State_AL | 25.4739 | 3.1045 |
| State_AR | 18.8049 | 6.4715 |  | State_AR | 24.8402 | 3.1056 |
| State_AZ | 20.4408 | 6.4018 |  | State_AZ | 25.8599 | 3.0868 |
| State_CA | 20.6975 | 6.3185 |  | State_CA | 25.8176 | 3.0984 |
| State_CO | 19.8122 | 6.3610 |  | State_CO | 25.3862 | 3.1116 |
| State_CT | 20.2207 | 6.3032 |  | State_CT | 25.5396 | 3.1095 |
| State_DC | 19.1795 | 6.4386 |  | State_DC | 25.3363 | 3.1291 |
| State_DE | 19.1347 | 6.4339 |  | State_DE | 25.2623 | 3.1247 |
| State_FL | 20.5722 | 6.3694 |  | State_FL | 25.9208 | 3.0801 |
| State_GA | 20.2027 | 6.3739 |  | State_GA | 25.6486 | 3.1062 |
| State_HI | 16.3756 | 6.5578 |  | State_HI | 23.1358 | 3.1229 |
| State_IA | 19.3850 | 6.4226 |  | State_IA | 25.3599 | 3.1205 |
| State_ID | 17.5900 | 6.4901 |  | State_ID | 24.5220 | 3.1161 |
| State_IL | 20.6240 | 6.2976 |  | State_IL | 25.7243 | 3.1100 |
| State_IN | 20.0560 | 6.3520 |  | State_IN | 25.5083 | 3.1131 |
| State_KS | 18.6333 | 6.4643 |  | State_KS | 24.8764 | 3.1149 |
| State_KY | 19.0667 | 6.4266 |  | State_KY | 25.0219 | 3.1148 |
| State_LA | 20.1784 | 6.3796 |  | State_LA | 25.5226 | 3.1029 |
| State_MA | 20.5175 | 6.2648 |  | State_MA | 25.6417 | 3.1092 |
| State_MD | 20.1825 | 6.3528 |  | State_MD | 25.6264 | 3.1136 |
| State_ME | 17.7800 | 6.4614 |  | State_ME | 24.1932 | 3.1233 |
| State_MI | 20.2474 | 6.2910 |  | State_MI | 25.4264 | 3.1023 |
| State_MN | 19.8153 | 6.3778 |  | State_MN | 25.4916 | 3.1192 |
| State_MO | 19.5278 | 6.4086 |  | State_MO | 25.3725 | 3.1141 |
| State_MS | 19.7692 | 6.4197 |  | State_MS | 25.4946 | 3.1064 |
| State_MT | 17.0798 | 6.5162 |  | State_MT | 23.9490 | 3.1228 |
| State_NC | 19.9077 | 6.4025 |  | State_NC | 25.5173 | 3.1091 |
| State_ND | 17.8617 | 6.4868 |  | State_ND | 24.4207 | 3.1245 |
| State_NE | 18.7804 | 6.4646 |  | State_NE | 24.9285 | 3.1161 |
| State_NH | 18.9226 | 6.4403 |  | State_NH | 25.0913 | 3.1225 |
| State_NJ | 20.9630 | 6.2521 |  | State_NJ | 25.6228 | 3.0983 |
| State_NM | 19.2552 | 6.4637 |  | State_NM | 25.3113 | 3.1179 |
| State_NV | 19.1947 | 6.4700 |  | State_NV | 25.3822 | 3.1105 |
| State_NY | 21.0080 | 6.2125 |  | State_NY | 25.5172 | 3.0922 |
| State_OH | 20.1500 | 6.3364 |  | State_OH | 25.5376 | 3.1135 |
| State_OK | 18.8991 | 6.4555 |  | State_OK | 25.0813 | 3.1151 |
| State_OR | 18.3544 | 6.4661 |  | State_OR | 24.8886 | 3.1143 |
| State_PA | 20.6973 | 6.2977 |  | State_PA | 25.6853 | 3.1083 |
| State_RI | 19.3915 | 6.3891 |  | State_RI | 25.3627 | 3.1225 |
| State_SC | 19.6460 | 6.4231 |  | State_SC | 25.6056 | 3.0982 |
| State_SD | 18.1443 | 6.4931 |  | State_SD | 24.6300 | 3.1185 |
| State_TN | 19.3073 | 6.4345 |  | State_TN | 25.2591 | 3.1035 |
| State_TX | 20.3483 | 6.3769 |  | State_TX | 26.0688 | 3.0804 |
| State_UT | 18.5798 | 6.4782 |  | State_UT | 24.9763 | 3.1110 |
| State_VA | 19.8965 | 6.3755 |  | State_VA | 25.5300 | 3.1158 |
| State_VT | 17.1740 | 6.4818 |  | State_VT | 23.5550 | 3.1235 |
| State_WA | 19.3864 | 6.3950 |  | State_WA | 25.2778 | 3.1108 |
| State_WI | 19.0798 | 6.3917 |  | State_WI | 25.1346 | 3.1162 |
| State_WV | 17.9549 | 6.5009 |  | State_WV | 24.4299 | 3.1247 |
| State_WY | 16.4030 | 6.4989 |  | State_WY | 23.1723 | 3.1252 |
| t0 [LogDays] | -5.9709 | 3.0966 |  | t0 [LogDays] | -9.9776 | 1.4544 |
| t2 [LogDays^2] | 0.5970 | 0.3740 |  | f1 [LogFracBase] | 0.1709 | 0.1155 |
| b1 [L7.LogDailyDead] | 0.4261 | 0.0798 |  | t2 [LogDays^2] | 1.1335 | 0.1683 |
| w1 [L7.LogMaxTemp] | 0.3841 | 0.0990 |  | b1 [L7.LogDailyDead] | 0.8716 | 0.0296 |
| w2 [L14.LogMaxTemp] | 0.4615 | 0.0812 |  | w1 [L7.LogMaxTemp] | 0.3263 | 0.0441 |
|  |  |  |  | w2 [L14.LogMaxTemp] | 0.3122 | 0.0529 |
| R-square | 0.6395 |  |  |  |  |  |
| Root MSE | 35.5107 |  |  | R-square | 0.9712 |  |
| Observations | 4567 |  |  | Root MSE | 8.5217 |  |
|  |  |  |  | Observations | 4565 |  |

| **Model 28** |  |  |  |
| --- | --- | --- | --- |
| Variable | Coefficient | S.E. |  |
| State_AK | 22.8014 | 3.6301 |  |
| State_AL | 25.0305 | 3.6241 |  |
| State_AR | 24.3929 | 3.6230 |  |
| State_AZ | 25.3532 | 3.6086 |  |
| State_CA | 25.3838 | 3.6185 |  |
| State_CO | 24.8946 | 3.6273 |  |
| State_CT | 25.0881 | 3.6210 |  |
| State_DC | 24.7892 | 3.6367 |  |
| State_DE | 24.8232 | 3.6379 |  |
| State_FL | 25.4345 | 3.6020 |  |
| State_GA | 25.1962 | 3.6266 |  |
| State_HI | 22.6422 | 3.6361 |  |
| State_IA | 24.9158 | 3.6367 |  |
| State_ID | 24.0270 | 3.6253 |  |
| State_IL | 25.2666 | 3.6274 |  |
| State_IN | 25.0734 | 3.6284 |  |
| State_KS | 24.4281 | 3.6302 |  |
| State_KY | 24.6016 | 3.6292 |  |
| State_LA | 25.0493 | 3.6256 |  |
| State_MA | 25.2137 | 3.6205 |  |
| State_MD | 25.1684 | 3.6305 |  |
| State_ME | 23.7875 | 3.6289 |  |
| State_MI | 24.9994 | 3.6144 |  |
| State_MN | 25.0566 | 3.6347 |  |
| State_MO | 24.9280 | 3.6307 |  |
| State_MS | 25.0477 | 3.6270 |  |
| State_MT | 23.4948 | 3.6320 |  |
| State_NC | 25.0630 | 3.6281 |  |
| State_ND | 24.0245 | 3.6367 |  |
| State_NE | 24.4875 | 3.6324 |  |
| State_NH | 24.6537 | 3.6344 |  |
| State_NJ | 25.1534 | 3.6132 |  |
| State_NM | 24.8402 | 3.6368 |  |
| State_NV | 24.8654 | 3.6276 |  |
| State_NY | 25.0551 | 3.6058 |  |
| State_OH | 25.1222 | 3.6276 |  |
| State_OK | 24.6560 | 3.6319 |  |
| State_OR | 24.4467 | 3.6261 |  |
| State_PA | 25.2259 | 3.6240 |  |
| State_RI | 24.9235 | 3.6326 |  |
| State_SC | 25.1675 | 3.6177 |  |
| State_SD | 24.2066 | 3.6324 |  |
| State_TN | 24.8118 | 3.6207 |  |
| State_TX | 25.5980 | 3.6010 |  |
| State_UT | 24.5284 | 3.6246 |  |
| State_VA | 25.1033 | 3.6334 |  |
| State_VT | 23.3076 | 3.6287 |  |
| State_WA | 24.8037 | 3.6215 |  |
| State_WI | 24.7188 | 3.6271 |  |
| State_WV | 23.9990 | 3.6367 |  |
| State_WY | 22.7585 | 3.6357 |  |
| t0 [LogDays] | -9.9198 | 1.6788 |  |
| t2 [LogDays^2] | 1.1308 | 0.1946 |  |
| b1 [L7.LogDailyDead] | 0.8743 | 0.0262 |  |
| w1 [L7.LogMaxTemp] | 0.2750 | 0.0255 |  |
| w2 [L14.LogMaxTemp] | 0.2326 | 0.0496 |  |
|  |  |  |  |
| R-square | 0.9707 |  |  |
| Root MSE | 8.5918 |  |  |
| Observations | 4567 |  |  |
